# Impact of the Cultivation Technique on the Production of Secondary Metabolites by *Chrysosporium lobatum* TM-237-S5, Isolated from the Sponge *Acanthella cavernosa*

**DOI:** 10.3390/md17120678

**Published:** 2019-11-30

**Authors:** Géraldine Le Goff, Philippe Lopes, Guillaume Arcile, Pinelopi Vlachou, Elsa Van Elslande, Pascal Retailleau, Jean-François Gallard, Michal Weis, Yehuda Benayahu, Nikolas Fokialakis, Jamal Ouazzani

**Affiliations:** 1 Institut de Chimie des Substances Naturelles ICSN, Centre National de la Recherche Scientifique CNRS, Avenue de la Terrasse, 91198 Gif-sur-Yvette, France; Philippe.lopes@cnrs.fr (P.L.); Guillaume.arcile@cnrs.fr (G.A.); elsa.van-elslande@cnrs.fr (E.V.E.); Pascal.retailleau@cnrs.fr (P.R.); jean-francois.gallard@cnrs.fr (J.-F.G.); jamal.ouazzani@cnrs.fr (J.O.); 2 Division of Pharmacognosy and Chemistry of Natural Products, Department of Pharmacy, National and Kapodistrian University of Athens, 15771 Athens, Greece; pvlachou@pharm.uoa.gr (P.V.); fokialakis@pharm.uoa.gr (N.F.); 3 School of Zoology, George S. Wise Faculty of Life Sciences, Tel Aviv University, Ramat Aviv, Tel Aviv 69978, Israel; mich9@tauex.tau.ac.il (M.W.); yehudab@tauex.tau.ac.il (Y.B.)

**Keywords:** solid-state fermentation, solid-state extraction, *Chrysosporium lobatum*, marine fungi, phenalenone derivatives

## Abstract

The fungi *Chrysosporium lobatum* TM-237-S5 was isolated from the sponge *Acanthella cavernosa*, collected from the mesophotic coral ecosystem of the Red Sea. The strain was cultivated on a potato dextrose agar (PDA) medium, coupling solid-state fermentation and solid-state extraction (SSF/SSE) with a neutral macroreticular polymeric adsorbent XAD Amberlite resin (AMBERLITE XAD1600N). The SSF/SSE lead to high chemodiversity and productivity compared to classical submerged cultivation. Ten phenalenone related compounds were isolated and fully characterized by one-dimensional and two-dimensional NMR and HRMS. Among them, four were found to be new compounds corresponding to isoconiolactone, (-)-peniciphenalenin F, (+)-8-hydroxyscleroderodin, and (+)-8-hydroxysclerodin. It is concluded that SSF/SSE is a powerful strategy, opening a new era for the exploitation of microbial secondary metabolites.

## 1. Introduction

The symbiosis between marine sponges and microorganisms is of considerable interest, both biologically and chemically [1,2]. Sponges are benthic organisms that have been colonizing different marine ecosystems, including coral reefs, for 600 million years [3,4]. Their survival under drastically changing conditions requires a variety of adaptations, including the evolving strategy of symbiosis with beneficial microorganisms, which has been taking place since the Precambrian Age [5]. The mutualism between marine sponges and microbial symbionts is mainly related to nutrition and defense [1,6], under the control of dedicated enzymes and active secondary metabolites [2]. Although sponges are the main source of bioactive molecules isolated from marine organisms, a certain amount of evidence indicates that they are biosynthesized by microbial symbionts [7,8]. This has also been corroborated by the massive presence of microorganisms in the mesophyl matrix of the sponges, representing around 50% of their biomass [9,10,11].

Fungi in the marine environment, and especially those associated with marine invertebrates, have been extensively investigated and reviewed [12,13]. A 2019 collaborative review highlighted the present state of knowledge and raised a multitude of open questions regarding the diversity and function of fungi in marine ecosystems [13]. 

The symbiont assemblages inside the sponge are well organized in biofilms or dense colonies and are stabilized in the skeleton network over time [14,15]. This certainly impacts their development steps and the expression of biosynthetic clusters of secondary metabolites because it is now well documented that, in fungi, secondary metabolism and life cycle among fungi are co-regulated at the genomic level [16,17,18].

This idea drives us to compare the metabolic profile of fungi cultivated on a gar slants and in liquid state. The result is that solid-state cultivation often leads to larger molecular diversity than classical liquid state fermentation LSF [19,20,21]. The major obstacle that stands against agar cultivation is the scale-up. In order to overcome such a challenge, we have developed specific innovative technologies, namely Platotex [22,23] and, more recently, Unifertex [24]. As we systematically coupled the culture of microorganisms with in-situ solid phase extraction (SPE), we also developed a specific SPE procedure for agar cultivation, termed solid-solid extraction (SSE) [25].

In the present study, we report the impact of agar-supported cultivation on the production of secondary metabolites by the marine fungi *Chrysosporium lobatum* TM-237-S5, isolated from the Red Sea sponge *Acanthella cavernosa*. *Chrysosporium lobatum* was previously reported in the literature as a mosquito pathogenic fungus [26]. However, a very limited number of secondary metabolites have been reported in the literature for the genus *Chrysosporium*. Thus, the strain *Chrysosporium queenslandicum* IFM produced naphthaquinone-type altersolanols A, B, and C, the antifungal queenslandon, a representative of the zearalenone family of mycotoxin, and the antibacterial dihydronaphthaquinones chrysoqueen and chrysolandol [27]. The diterpenoid derivative RPR113228, a farnesyl transferase inhibitor, was also attributed to *Chrysosporium lobatum*, yet the identification of the strain was only based on morphological analysis [28]. Furthermore, curvularin and dehydroculvilarin were isolated from *Chrysosporium lobatum* BK-3 [29]. 

## 2. Results and Discussion

### 2.1. The Context of This Work

**Figure 1:**
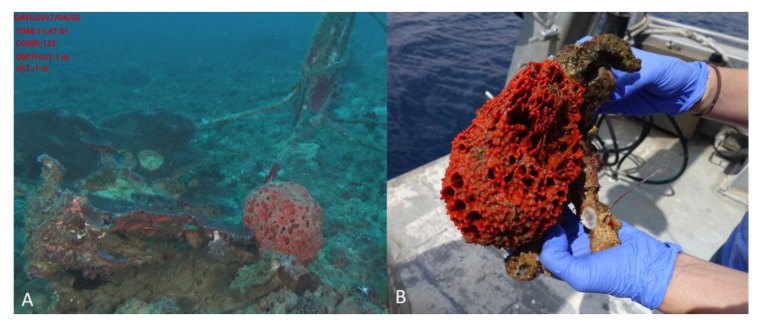
*Acanthella cavernosa* was collected at 51 m depth in Eilat, Gulf of Aqaba (Israel). (**A**) The sponge in its natural environment, (**B**) The sponge was collected by the remote operating vehicle (ROV) arm, introduced in-situ to the collection basket, and brought to the boat for immediate processing. Two representative pieces were recovered, one for taxonomic identification and the other for symbiont isolation. Both samples were immediately frozen on the boat and shipped in dry ice.

The TASCMAR project (Tools And Strategies to access to original bioactive compounds from Cultivation of MARine invertebrates and associated symbionts), funded by the European Union in the frame of the Horizon 2020 framework program, offered the opportunity to investigate the molecules produced by marine invertebrates and their symbionts from mesophotic coral ecosystems (MCEs) (30 to 150 m depth). Among the invertebrates investigated, the sponge *Acanthella cavernosa* was collected on the upper mesophotic reef of Eilat at Dekel Beach (51 m depth), in the Gulf of Aqaba (Israel, 2 April 2017, 29°32′12.48″N; 34°56′55.656″E). The area is characterized by a moderate slope covered with dense patches of hard substrate, mostly calcareous, and is also inhabited by other invertebrates such as octocorals, stony corals, black corals, and sea anemones (Figure 1).

**Figure 2:**
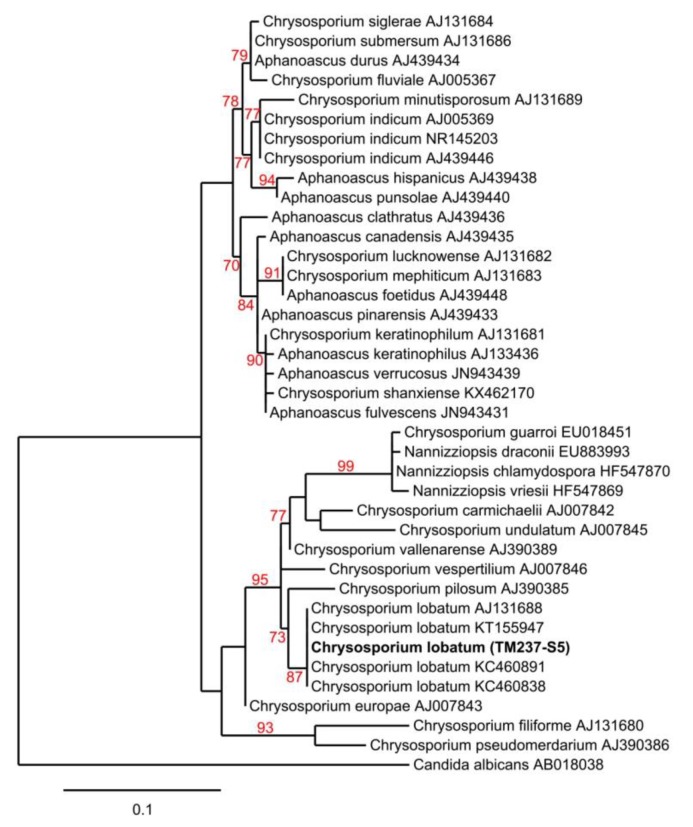
Maximum-likelihood tree obtained from ITS rDNA sequence alignment of the strain TM237-S5 and *Chrysosporium* spp. Reliability of the internal branch is represented in red. *Candida albicans* was used as the outgroup. Numbers are Genbank accessions. Th estrain in bold font is the one described in this study. Scale represents substitutions per site.

The strain *Chrysosporium lobatum* TM-237-S5 (Figure 2) was among the strains isolated and identified based on its ITS rDNA sequence (Nuclear ribosomal internal transcribed spacer).

The strain was cultivated on potato dextrose broth (PDB), potato dextrose agar (PDA), marine broth (MB), and marine agar (MA). Solid phase extraction (SPE) with XAD resin (AMBERLITE™ *XAD*™*16HP* N) was applied in-situ to both liquid (LSF/SPE) and agar-supported cultures (solid-state fermentation and solid-state extraction (SSF/SSE)). It has been previously reported that in-situ XAD extraction coupled to agar-supported cultivation prevents the diffusion of target compounds to the agar layer and traps the target compounds on the resin beads [25].

**Figure 3:**
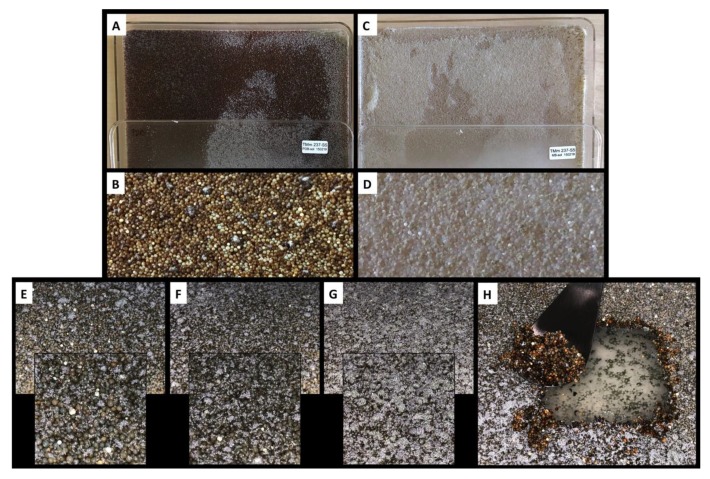
10 days culture of *Chrysosporium lobatum* TM-237-S5 on potato dextrose agar (PDA) (**A**,**B**) and marine agar (MA) (**C**,**D**) coupled to solid-solid extraction (SSE) with XAD resin (AMBERLITE™ *XAD*™*16HP* N). The resin beads remained white to light beige on the marine broth (MB) (**D**), while they turned dark brown on the PDA (**B**), showing that the resin beads trapped the colored compounds secreted by the strain. (**E**–**G**) present the coverage of the resin beads by the mycelium at four, seven, and 10 days. (**H**) depicts the easy recovery of the resin biofilm layers; the mycelium is not incrusted and no compounds flow to the agar. The resin beads, as revealed by the dark brown color, trapped all the produced compounds.

On day four of incubation (Figure 3E), the resin beads became colored, but were not yet covered by the mycelium (white filaments). On day seven (Figure 3F), the resin beads became darker and the mycelium surface increased. On day 10 (Figure 3G), the recovery time, the resin beads were totally covered by the mycelium. We previously reported that such phenomenon is probably due to the lack of oxygen in the viscous resin layer, which pushes the mycelium to reach the surface to access more oxygen. However, the mycelia remained in contact with the agar to access nutriments, as shown on the agar layer, following the recovery of the resin beads (Figure 3H).

**Figure 4:**
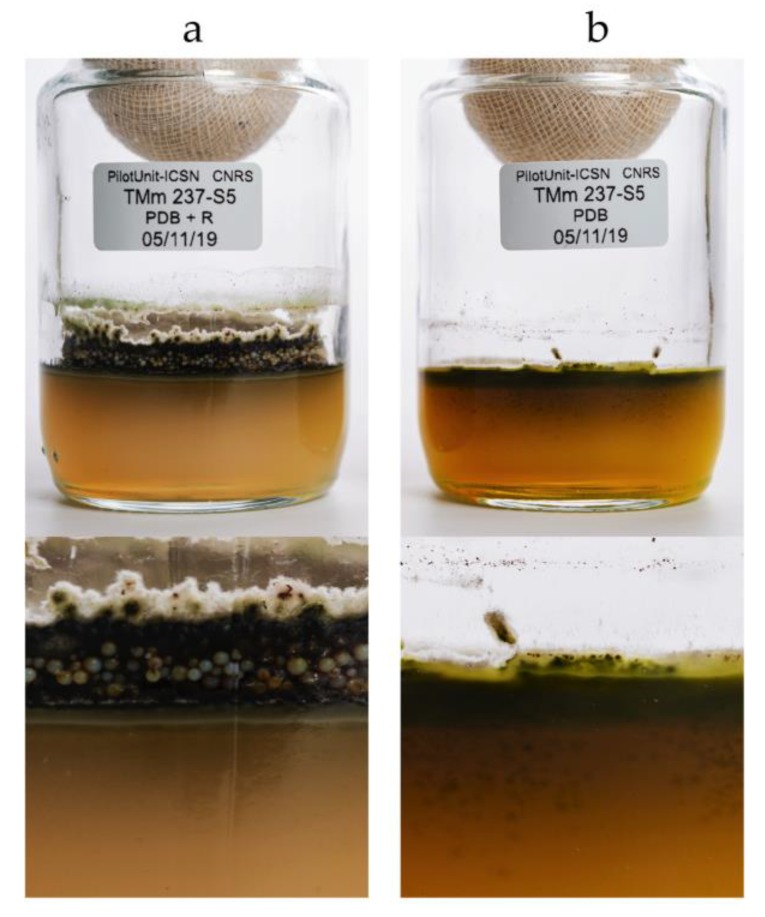
10 days culture of *Chrysosporium lobatum* TM-237-S5 on PDA coupled to SSE with *XAD*™*16HP* N (**a**) and the control culture on PDA without resin (**b**). Without the resin (**b**), the colored compounds were spread in the agar, and their extraction was difficult. With the resin (**a**), the agar remained clear as the resin beads trapped all the colored target compounds.

After 10 days of incubation, the resin beads were recovered by filtration from the liquid cultures (10 L), and by scraping the surface of the agar cultures (10 × 625 cm^2^ petri plates), and washed extensively with water to remove medium residues and any compounds not trapped by the XAD. Resin beads from the PDB, MB, and MA cultures had a light beige color, while the PDA culture was dark brown; most of the color being trapped by the resin beads (Figure 3A–D and Figure 4).

**Figure 5:**
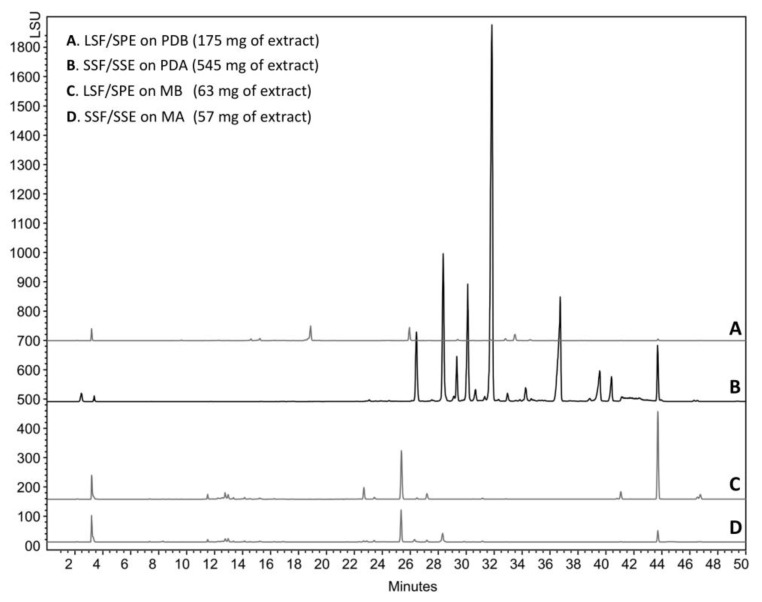
HPLC analysis of the ethyl acetate extract of *C. lobatum* TM-237-S5 cultivated on different media and support.

**Figure 6:**
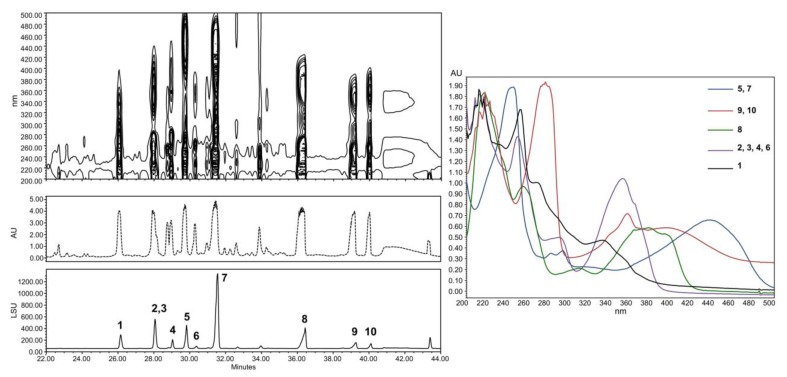
HPLC analysis with LSD and one-dimensional/two-dimensional PDA detections (right). Absorbance spectrum of the compounds investigated.

The compounds trapped in the XAD were eluted with ethyl acetate and analyzed by HPLC coupled to photodiode array PDA, light-scattering LSD, and mass spectrometry MS detectors (Figure 5). According to the recovered quantities of extracts and the diversity of metabolites observed in the chromatograms, the current study focused on the extract from agar-supported cultivation coupled to in-situ solid-state extraction (SSF/SSE, Figure 5B). This SSF/SSE on PDA lead to an overall extract yield of 872 mg/m^2^ of cultivation surface, corresponding to 2 L of medium (200 mL per plate). HPLC analysis revealed 10 peaks with specific UV absorption spectra (Figure 6), which were totally absent in the liquid culture (LSF/SPE) (Figure 5C). 

### 2.2. Structural Identification of Compounds **1** to **10**

**Figure 7:**
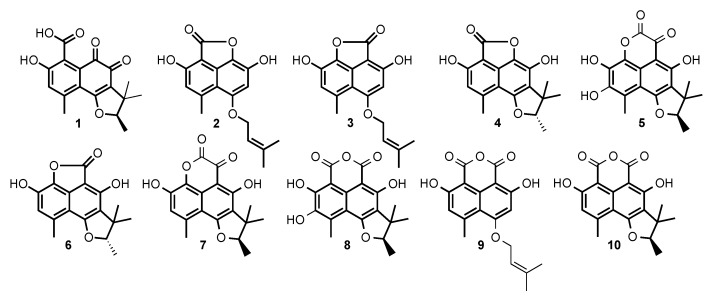
Structures of the compounds produced by *Chrysosporium lobatum* TM-237-S5, cultivated for 10 days on PDA medium coupling solid-state fermentation with solid-state extraction (SSF/SSE).

Compounds **1** to **10** in Figure 7 were purified and submitted to one-dimensional and two-dimensional NMR and HRMS analysis. Six compounds were unambiguously identified as peniciphenalenin D (**1**), isolated from *Pebnicillium* sp. ZZ901 [30], coniolactone (**3**), (-)-7,8-Dihydro-3,6-dihydroxy-1,7,7,8-tetramethyl-5H-furo-[2′,3′:5,6] naphtho[1,8-bc]furan-5-one (**6**), coniosclerodin (**9**), isolated from *Coniothyrium cereale* [30,31], (+)-scleroderolide (**7**), isolated from *Gremmeniella abietina* [32,33], and (+)-sclerodin (**10**), isolated from *Aspergillus silvaticus* [34]. 

Compounds **2**, **4**, **5**, and **8** were submitted to dereplication based on the Antibase database of microbial compounds (Wiley–VCH) and the natural compounds Reaxys database (Elsevier). Spectroscopic data of these compounds did not match the previously reported compounds or present significant differences, and were submitted to de-novo structural elucidation. Their ^1^H and ^13^C NMR data are shown in Table 1 and Table 2.

**Figure 8:**
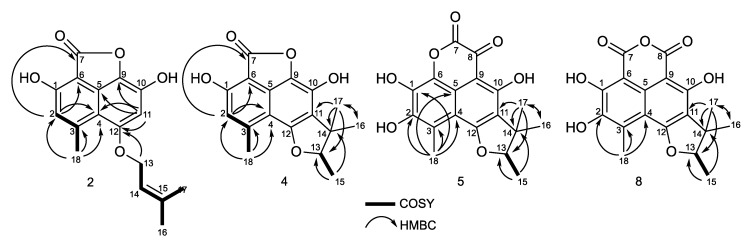
COSY and key HMBC correlations for compounds **2**, **4**, **5**, and **8**.

The peak at 28 min exhibits a molecular formula of C_17_H_16_O_5_, determined by HRESIMS (*m*/*z* 301.1076 [M + H]^+^). A careful ^1^H and ^13^C NMR analysis of the peak at 28 min revealed a mixture of two compounds with indistinguishable HRMS. De-replication and comparison with published results showed that one of the constituents was unambiguously coniolactone (**3**). As well as **3**, HMBC correlations showed that compound **2** differs only at the ring C configuration. A key HMBC correlation from H-2 (δ_H_ 6.70, s) to the carbonyl C-7 (δ_C_ 168.1) indicated that, in **2**, the carbonyl at C-7 is connected to C-6 rather than to C-9 in coniolactone (**3**) (Figure 8). So far, all our attempts to separate **2** and **3** by different chromatographic techniques have failed. Compound **2** was named isoconiolactone.

Compound **4** has a molecular formula C_17_H_16_O_5_, deduced from HRESIMS and NMR data (Table 1 and Table 2). According to NMR and MS data, **4** has the same planar structure as the already known fungal metabolite peniciphenalenin F [30]. However, compound **4** and peniciphenalenin F have an opposite optical rotation; negative for **4** (−36.10° (c 0.10, MeOH)) and positive for the reported peniciphenalenin F (+16.50° (c 0.50, MeOH)). Subsequently, **4** was named (-)-peniciphenalenin F.

Compound **5** had a molecular formula of C_18_H_16_O_7_, deduced from HRESIMS and NMR data (Table 1 and Table 2). The NRM data of **5** indicates the presence of two carbonyls, eight aromatic carbons, one oxymethine, one quaternary carbon, and four methyls. NMR comparison with previously reported phenalenone derivatives has shown similarities with the isolated (+)-scleroderolide (**7**) [32,33], except in the C-2 position. Indeed, the aromatic proton, H-2, of scleroderolide is substituted in **5** by a hydroxyl group in C-2 (δ_C_ 144.9). This finding is also supported by key HMBC correlations from H-18 (2.61, 3H, s) to C-2 (δ_C_ 144.9), C-3 (δ_C_ 120.5), and C-4 (δ_C_ 109.3). Accordingly, compound **5** has been identified as a new phenalenone derivative and was named (+)-8-hydroxyscleroderolide.

Compound **8** has a molecular formula C_18_H_16_O_7_, deduced from the HRESIMS and NMR data (Table 1 and Table 2). Here again, the NRM data of **8** showed the presence of two carbonyl, eight aromatic carbons, one oxymethine, one quaternary carbon, and four methyls. The NMR data of **8** closely resemble those of the previously described and isolated (+)-sclerodin (**10**) [34], except in the C-2 position. The aromatic proton, H-2, of the sclerodin structure is substituted in **8** by a hydroxyl group in C-2 (δ_C_ 140.4). This finding is supported by key HMBC correlations from H-18 (2.79, 3H, s) to C-2 (δ_C_ 140.4), C-3 (δ_C_ 130.3), and C-4 (δ_C_ 108.9). 

**Figure 9:**
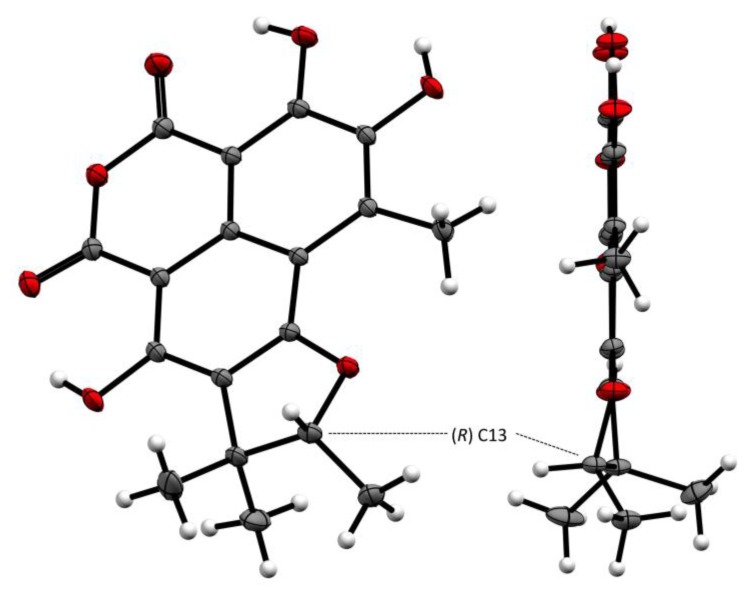
The Oak Ridge Thermal Ellipsoid Plot ORTEP diagram of **8**.

The structure of **8**, including the absolute configuration (13*R*), is secured by a single crystal X-ray crystallographic analyses using anomalous scattering of Mo then CuK α radiation through Bijvoet analysis [35], combining maximum likelihood estimation and Bayesian statistics (Figure 9). Therefore, compound **8** was named (+)-8-hydroxyslerodin.

The current study presents the breakthrough advantage of solid-state fermentation coupled with in-situ solid phase extraction (SSF/SSE). Indeed, SSF/SSE leads to a large chemical diversity and higher yields. In addition, the concentration of the target compounds on the resin beads represents an economic and ecofriendly recovery process involving limited quantities of water for medium preparation, limited use of power for static incubation, a limited amount of solvents for the elution of compounds, and reduced waste. We also solved the issue of scale-up on the Platotex technology, offering a reusable 2 m^2^ cultivation surface, and more recently on the Unifertex technology.

## 3. Materials and Methods 

### 3.1. General Experimental Procedures

Optical rotations, [α]_D_, were measured using an Anton Paar MCP-300 polarimeter at 589 nm (Anton Paar, Les Ulis, France). NMR experiments were performed using a Bruker Avance III 600 MHz spectrometer equipped with a TCi cryo-probe head for compounds **8**, **9**, and **10**, and a Bruker Avance 500 MHz spectrometer for compounds **1** to **7** (Bruker, Vienna, Austria). The spectra were acquired in CD_3_OD (δ_H_ 3.31 ppm and δ_C_ 49.15 ppm), in CD_2_Cl_2_ (δ_H_ 5.32 ppm and δ_C_ 53.10 ppm) or in Acetone-*d*_6_ (δ_H_ 2.04 ppm and δ_C_ 29.8 ppm and 206.5 ppm) at 300K. High-resolution mass spectra were obtained on a Waters LCT Premier XE spectrometer equipped with an electrospray-time of flight (ESI-TOF) by direct infusion of the purified compounds (Waters SAS, Saint-Quentin-en-Yvelines, France). Pre-packed silica gel Redisep columns were used for flash chromatography using a Combiflash-Companion chromatogram (Serlabo, Entraigues-sur-la-Sorgue, France). All other chemicals and solvents were purchased from SDS (SDS, Peypen, France).

The analytical HPLC system consisted of an Alliance Waters 2695 controller coupled with a PhotoDiode Array Waters 2996, an evaporative light-scattering detector ELSD Waters 2424 detector and a mass detector Waters QDa (Waters SAS, Saint-Quentin-en-Yvelines, France). A Sunfire C_18_ column (4.6 × 150 mm, 3.5 µm) was used with a flow rate of 0.7 mL/min. The elution gradient consisted of a linear gradient from 100% solvent A to 100% solvent B in 40 min, then 10 min at 100% B (Solvent A: H_2_O + 0,1 HCOOH, Solvent B: ACN + 0,1% HCOOH). Preparative HPLC was performed on a semi-preparative Sunfire C_18_ column (10 × 250 mm, 5 µm) using a Waters autosampler 717, a pump 600, a photodiode array detector 2996, and an ELSD detector 2420 (Waters SAS, Saint-Quentin-en-Yvelines, France). XAD resin (AMBERLITE™ *XAD*™*16HP* N) was purchased from DOW (DOW France SAS, Saint-Denis, France).

### 3.2. Invertebrate Collection

The sponge, *Acanthella cavernosa*, was collected by a ROV H800 (ECA, Lannion, France) on the upper mesophotic reef of Eilat at Dekel Beach at 51 m depth in the Gulf of Aqaba, Israel (2 April 2017, 29°32′12.48″N; 34°56′55.656″E). The sponge was identified by Dr. Nicole J. de Voogd from Naturalis, Biodiversity Research Center, Leiden, the Netherlands. Collection of animals complied with a permit issued by the Israel Nature and National Parks Protection Authority.

### 3.3. Strain Isolation and Identification

*Chrysosporium lobatum* TM-237-S5 was isolated from a 1 cm^3^ sample of *Acanthella cavernosa*. The invertebrate was immediately stored after collection and conserved at −20 °C until lab work processing. Part of the invertebrate (1 cm^3^) was ground in sterile sea water and heated at 50 °C for 1 h. The suspension was serially diluted, plated on selective isolation media, and incubated at 28 °C for at least 6 weeks. The strain was isolated from marine agar medium. The colony was purified on PDA and MA media and preserved in 10% glycerol solution.

Genomic DNA of the strain TM237-S5 was isolated using a DNeasy Plant Mini Kit (Qiagen), according to the manufacturer’s instructions. The ITS region was amplified with primers ITS1F (5′-CTTGGTCATTTAGAGGAAGTAA -3′) and ITS4 (5′-TCCTCCGCTTATTGATATGC) using described polymerase chain reaction (PCR) conditions [36]. Amplicons were sequenced by Sanger sequencing (GATC, Eurofins genomics), and the sequences were aligned against the non-redundant database of the NCBI using the BLASTn program. Then, phylogeny inference was performed on the Phylogeny.fr platform and comprised the following steps [37]: Sequences from TM237-S5 and representative *Chrysosporium* and *Chrysosporium*-related sequences described by Gurung et al. (2018) were aligned with MUSCLE (v3.8.31) [38]. After alignment, ambiguous regions were removed with Gblocks (v0.91b) [39]. The phylogenetic tree was reconstructed using the maximum likelihood method implemented in the PhyML program (v3.0 aLRT) [40], and reliability for internal branch was assessed using the aLRT test [41]. Graphical representation and edition of the phylogenetic tree were performed with TreeDyn (v198.3) [42]. The strain *Chrysosporium lobatum* TM237-S5 was assigned the GenBank number MN080876.

### 3.4. Microbial Cultivation

*Chrysosporium lobatum* TM-237-S5 spores were conserved at −20 °C in 10% glycerol. Before cultivation, the strain was revived for 5 days on a 15 cm petri plate containing potato dextrose agar (PDA). Sterile water (4 × 10 mL) was poured on the plate surface, and the spores were recovered from the plates by gentle scratching of the surface with a scalpel. Three plates offer 100 mL of concentrated spore suspension. Ten bottles were filled with 30 g of XAD Resin (AMBERLITE™ XAD™16HP N) and sterilized. 10 mL of Water and 10 mL of spore suspension were introduced in each resin containing bottle. The mixture was stirred, poured on a 25 × 25 cm petri plate containing PDA medium, homogeneously spread on the surface of the plate, and incubated at 27 °C. Preparative cultures were engaged on 10 25 × 25 cm petri plates.

### 3.5. Extraction/Purification Procedures

On day 10 of incubation, the resin/mycelium layer was recovered from the surface of the plates and re-suspended three times in 500 mL of Ethyl Acetate. Traces of water were removed on anhydrous sodium sulfate, and the solvent evaporated under reduced pressure. 550 mg of dry extract was recovered and submitted for analytical and structural analysis. The extract was fractionated by flash chromatography using a Combiflash-companion chromatogram, and the compounds were further purified by preparative HPLC to offer 6 to 56 mg of pure compound.

### 3.6. Structural Elucidation

All the compounds were submitted to one-dimensional and two-dimensional NMR analysis, high resolution mass spectrometry, and, when appropriate, to crystallography.

Peniciphenalenin D (**1**): Red amorphous solid (31 mg); [α]_D_^25^: +119.70° (*c* 0.1, MeOH); UV (MeOH) λ_max_ (log ε) 217 (3.83), 279 (3.85), 369 (2.91), 515 (2.90) nm; ^1^H and ^13^C NMR data, see Supplementary Information; HRESIMS *m*/*z* 317.1005 [M + H]^+^ (calcd. for C_17_H_17_O_6_, 317.1025).

Isoconiolactone (**2**) + Coniolactone (**3**): Orange amorphous powder (40 mg); UV (MeOH) λ_max_ (log ε) 220 (3.52), 254 (2.54), 346 (1.45) nm; ^1^H and ^13^C NMR data, Table 1; Table 2; HRESIMS *m*/*z* 301.1067 [M + H]^+^ (calcd. for C_17_H_17_O_5_, 301.1076)

(−)-Peniciphenalenin F (**4**): Yellowish amorphous powder (15 mg); [α]_D_: −36.10° (c 0.1, MeOH); UV (MeOH) λ_max_ (log ε) 221 (4.22), 274 (4.21), 357 (3.51), 393 (3.33) nm; ^1^H and ^13^C NMR data, Table 1; Table 2; HRESIMS *m*/*z* 301.1053 [M + H]^+^ (calcd. for C_17_H_17_O_5_, 301.1049)

(+)-8-Hydroxyscleroderolide (**5**): Pale yellowish amorphous powder (36 mg); [α]_D_: +65.01° (c 0.1, MeOH); UV (MeOH) λ_max_ (log ε) 244 (3.56), 448 (1.71) nm; ^1^H and ^13^C NMR data, Table 1; Table 2; HRESIMS *m*/*z* 345.0967 [M + H]^+^ (calcd. for C_18_H_17_O_7_, 345.0974).

(−)-7,8-Dihydro-3,6-dihydroxy-1,7,7,8-tetramethyl-5H-furo-[2′,3′:5,6]naphtho[1,8-bc]furan-5-one (**6**): Yellowish amorphous powder (5 mg); [α]_D_: −36.80° (c 0.1, MeOD); UV (MeOH) λ_max_ (log ε) 226 (1.22), 263 (3.65), 359 (3.52) nm; ^1^H and ^13^C NMR data, see Supplementary Information; HRESIMS *m*/*z* 301.1064 [M + H]^+^ (calcd. for C_17_H_17_O_5_, 301.1076).

(+)-Scleroderolide (**7**): Yellow powder (57 mg); [α]_D_: +73.0° (c 0.1, MeOD); UV (MeOH) λ_max_ (log ε) 226 (3.88), 250 (2.87), 294 (1.22), 439 (2.12) nm; ^1^H and ^13^C NMR data, see Supplementary Information; HRESIMS *m*/*z* 329.1033 [M + H]^+^ (calcd. for C_18_H_17_O_6_, 329.1039).

(+)-8-Hydroxysclerodin (**8**): Pale yellow crystal (38 mg); [α]_D_: +66.01° (c 0.1, MeOH); UV (MeOH) λ_max_ (log ε) 223 (3.38), 308 (1.14), 358 (2.52) nm; ^1^H and ^13^C NMR data, Table 1; Table 2; HRESIMS *m*/*z* 345.0960 [M + H]^+^ (calcd. for C_18_H_17_O_7_, 345.0961).

Coniosclerodin (**9**): Pale yellow powder (8 mg); UV (MeOH) λ_max_ (log ε) 250 (4.12), 289 (1.14), 351 (3.52) nm; ^1^H and ^13^C NMR data, see Supplementary Information; HRESIMS *m*/*z* 329.1001 [M + H]^+^ (calcd. for C_18_H_17_O_6_, 329.1025).

(+)-Sclerodin (**10**): Yellowish powder (7 mg); [α]_D_: +20.01° (c 0.10, CH_2_Cl_2_); UV (MeOH) λ_max_ (log ε) 216 (3.25), 256 (4.13), 295 (1.24), 359 (3.98) nm; ^1^H and ^13^C NMR data, see Supplementary Information; HRESIMS *m*/*z* 329.1015 [M + H]^+^ (calcd. for C_18_H_17_O_6_, 329.1025).

### 3.7. X-ray Crystal Structure Analysis 

High-resolution crystallographic data for compound **8** were collected using redundant ω scans on a Rigaku XtaLabPro single-crystal diffractometer using microfocus Mo K*α* radiation and a HPAD PILATUS3 R 200K detector. Its structure was readily solved by intrinsic phasing methods (*SHELXT*) and by full-matrix least-squares methods on F2 using *SHELX-L* [43,44]. The non-hydrogen atoms were refined anisotropically, and hydrogen atoms, all identified in difference maps, were positioned geometrically and refined with U_iso_ set to xU_eq_ of the parent atom (x = 1.5 for methyl carbons or hydroxy oxygens and 1.2 for all others). Despite extremely weak anomalous signal and ambiguous Flack parameter [45] obtained with that radiation, the Bijvoet analysis using likelihood methods showed strong probabilities that this characterized enantiopure natural product is (*R*)-2,3,7-trihydroxy-1,8,8,9-tetramethyl-8,9-dihydro-4*H*,6*H*-benzo[*de*]furo[2,3-*g*] isochromene-4,6-dione. Duplicated measurements using a Rigaku mm007 rotating anode consolidated our statement (data not deposited).

Crystallographic data for this structure, **8**, have been deposited in the Cambridge Crystallographic Data Centre database (CCDC) (deposition number CCDC 1963851). Copies of the data can be obtained free of charge from the CCDC at www.ccdc.cam.ac.uk. 

Crystal data for **8**: C18H16O7, *M* = 344.31, Orthorhombic, a = 6.6249(2) Å, b = 10.0615(3) Å, c = 22.5473(6) Å a = b = g = 90°, V = 1502.92(8) Å3, T = 293(2) K, space group *P*212121, Z = 4, *μ*(Mo K*α*) = 0.118 mm−1, 47,173 reflections measured, 4370 independent reflections (Rint = 0.0456). The final R1 values were 0.0389 (I > 2σ(I)). The final *wR* (F2) values were 0.1093 (all data). The goodness of fit on F2 was 1.072. Flack parameter = −0.2 (2). Bijvoet Pairs = 1854 (100% coverage): P2 (true) = 1.000. P3 (true) = 0.987, P3 (rac-twin) = 0.013, P3 (false) = 0.8.10^−6^. Hooft parameter = −0.1 (2) [35].

## Figures and Tables

**Table 1:** ^13^C NMR (125 and 150 MHz) of compounds **2**, **4**, **5**, and **8**.

Position	δ_C_, Type			
	2 ^a^	4 ^a^	5 ^a^	8 ^b^
1	160.2, C	160.2, C	138.9, C	160.0, C
2	118.9, CH	118.5, C	144.9, C	140.4, C
3	149.1, C	147.8, C	120.5, C	130.3, C
4	112.5, C	108.2, C	109.3, C	108.9, C
5	140.2, C	132.9, C	132.9, C	131.9, C
6	100.0, C	100.1, C	117.4, C	92.9, C
7	168.1, C	168.7, C	170.8, C	165.7, C
8	-	-	171.2, C	165.7, C
9	126.5, C	127.4, C	108.1, C	91.8, C
10	139.5, C	139.3, C	166.7, C	162.8, C
11	99.8, CH	122.1, C	120.5, C	119.8, C
12	156.4, C	153.8, C	158.5, C	153.9, C
13	66.8, CH_2_	91.4, CH	93.7, CH	91.7, CH
14	120.8, CH	46.0, C	44.3, C	43.4, C
15	139.2, C	14.6, CH_3_	14.9, CH_3_	14.1, CH_3_
16	25.8, CH_3_	21.3, CH_3_	21.2, CH_3_	20.3, CH_3_
17	18.3, CH_3_	26.3, CH_3_	25.9, CH_3_	25.2, CH_3_
18	23.0, CH_3_	21.4, CH_3_	13.7, CH_3_	13.9, CH_3_

^a,b^; the spectra were recorded in MeOD and CD_2_Cl_2_, respectively.

**Table 2:** ^1^H NMR (500 and 600 MHz) of compounds **2**, **4**, **5** and **8**.

Position	δ_H_, Mult. (*J* in Hz)			
	2 ^a^	4 ^a^	5 ^a^	8 ^b^
1	-	-	-	
2	6.70, s	6.70, s	-	
3	-	-	-	
4	-	-	-	
5	-	-	-	
6	-	-	-	
7	-	-	-	
8	-	-	-	
9	-	-	-	
10	-	-	-	
11	6.35,s	-	-	
12	-	-	-	
13	4.60, d (*6.4*)	4.50, q (*6.5*)	4.78, q (*6.6*)	4.71, q (*6.6*)
14	5.57, br m	-	-	-
15	-	1.43, d (*6.5*)	1.52, d (*6.7*)	1.50, d (*6.6*)
16	1.83, s	1.23, s	1.31, s	1.30, s
17	1.80, s	1.50, s	1.56, s	1.54, s
18	2.76, s	2.73, s	2.61, s	2.79, s
OH-10				11.36, s

^a,b^; the spectra were recorded in MeOD and CD_2_Cl_2_, respectively.

## Supplementary Materials

The following are available online at https://www.mdpi.com/1660-3397/17/12/678/s1, S1 to S4, spectroscopic characterization of compound **1**; S5 to S11, spectroscopic characterization of compounds **2** + **3**; S12 to S18, spectroscopic characterization of compound **4**; S19 to S25, spectroscopic characterization of compound **5**; S26 to S29, spectroscopic characterization of compound **6**, S30 to S34, spectroscopic characterization of compound **7**; S35 to S42, spectroscopic characterization of compound **8**; S43 to S46, spectroscopic characterization of compound **9**; S47 to S50, spectroscopic characterization of compound **10**; S51 to S57, crystallographic data for compound **8**; S58-S72, crystallographic data for compounds **7** and **10**.
