# Supplementary material for: Impact of the Cultivation Technique on the Production of Secondary Metabolites by Chrysosporium lobatum TM-237-S5, Isolated from the Sponge Acanthella cavernosa
Source: Mar Drugs. 2019 Nov 30;17(12):678. doi: 10.3390/md17120678 (PMC6950079; doi:10.3390/md17120678)
Supplement: Supplementary file 1 [file marinedrugs-17-00678-s001.pdf]

Supplementary Materials

Impact of the cultivation technique on the production of secondary metabolites by *Chrysosporium lobatum* TM-237-S5, isolated from the sponge *Acanthella cavernosa*.

Géraldine Le Goff [1,*], Philippe Lopes [1], Guillaume Arcile [1], Pinelopi Vlachou [2], Elsa Van Elslande [1], Pascal Retailleau [1], Jean-François Gallard [1], Michal Weis [3], Yehuda Benayahu [3], Nikolas Fokialakis [2] and Jamal Ouazzani [1].

[1] Institut de Chimie des Substances Naturelles ICSN, Centre National de la Recherche Scientifique CNRS, Avenue de la Terrasse 91198, Gif-sur-Yvette, France; geraldine.legoff@cnrs.fr (G.L.G); Philippe.lopes@cnrs.fr (P.L); Guillaume.arcile@cnrs.fr (G.A); elsa.van-elslande@cnrs.fr (E.V-E); Pascal.retailleau@cnrs.fr (P.R); jean-francois.gallard@cnrs.fr (J-F. G); jamal.ouazzani@cnrs.fr (J.O)
[2] Department of Pharmacognosy & Natural Products Chemistry, Faculty of Pharmacy, National and Kapodistrian University of Athens, Athens 15771 Greece; pvlachou@pharm.uoa.gr (P.V); fokialakis@pharm.uoa.gr (N.F)
[3] Department of Zoology, George S. Wise Faculty of Life Sciences, Tel Aviv University, Tel Aviv, Israel, mich9@tauex.tau.ac.il (M.W); yehudab@tauex.tau.ac.il (Y.B)

S1. ^{1}H NMR spectrum (500MHz, Acetone-d_6) of **1**
S2. ^{13}C NMR spectrum (125MHz, Acetone-d6) of **1**
S3. HRESIMS $[M+H]^+$ of **1**
S4. HRESIMS $[M-H]^-$ of **1**
S5. ^{1}H NMR spectrum (500MHz, MeOD) of **2** + **3**
S6. ^{13}C NMR spectrum (125MHz, MeOD) of **2** + **3**
S7. ^{1}H-^{1}H COSY NMR spectrum (500MHz, MeOD) of **2** + **3**
S8. ^{1}H-^{13}C HSQC spectrum (500 MHz, MeOH) of **2** + **3**
S9. ^{1}H-^{13}C HMBC spectrum (500 MHz, MeOH) of **2** + **3**
S10. HRESIMS $[M+H]^+$ of **2** + **3**
S11. HRESIMS $[M-H]^-$ of **2** + **3**
S12. ^{1}H NMR spectrum (500MHz, MeOD) of **4**
S13. ^{13}C NMR spectrum (125MHz, MeOD) of **4**
S14. ^{1}H-^{1}H COSY NMR spectrum (500MHz, MeOD) of **4**
S15. ^{1}H-^{13}C HSQC spectrum (500 MHz, MeOH) of **4**
S16. ^{1}H-^{13}C HMBC spectrum (500 MHz, MeOH) of **4**
S17. HRESIMS $[M+H]^+$ of **4**
S18. HRESIMS $[M-H]^-$ of **4**
S19. ^{1}H NMR spectrum (500MHz, MeOD) of **5**
S20. ^{13}C NMR spectrum (125MHz, MeOD) of **5**
S21. ^{1}H-^{1}H COSY NMR spectrum (500MHz, MeOD) of **5**
S22. ^{1}H-^{13}C HSQC spectrum (500 MHz, MeOH) of **5**
S23. ^{1}H-^{13}C HMBC spectrum (500 MHz, MeOH) of **5**
S24. HRESIMS $[M+H]^+$ of **5**
S25. HRESIMS $[M-H]^-$ of **5**
S26. ^{1}H NMR spectrum (500MHz, Acetone-d_6) of **6**
S27. ^{13}C NMR spectrum (125MHz, Acetone-d6) of **6**
S28. HRESIMS $[M+H]^+$ of **6**
S29. HRESIMS $[M-H]^-$ of **6**
S30. X-ray crystallographic data of compound **7**
S31. ^{1}H NMR spectrum (500MHz, Acetone-d_6) of **7**
S32. ^{13}C NMR spectrum (125MHz, Acetone-d6) of **7**
S33. HRESIMS $[M+H]^+$ of **7**
S34. HRESIMS $[M-H]^-$ of **7**
S35. X-ray crystallographic data of compound **8**
S36. ^{1}H NMR spectrum (500MHz, CH_2Cl_2) of **8**
S37. ^{13}C NMR spectrum (125MHz, CH_2Cl_2) of **8**
S38. ^{1}H-^{1}H COSY NMR spectrum (500MHz, CH_2Cl_2) of **8**
S39. ^{1}H-^{13}C HSQC spectrum (500 MHz, CH_2Cl_2) of **8**
S40. ^{1}H-^{13}C HMBC spectrum (500 MHz, CH_2Cl_2) of **8**
S41. HRESIMS $[M+H]^+$ of **8**
S42. HRESIMS $[M-H]^-$ of **8**
S43. ^{1}H NMR spectrum (500MHz, CD_2Cl_2) of **9**
S44. ^{13}C NMR spectrum (125MHz, CD_2Cl_2) of **9**
S45. HRESIMS $[M+H]^+$ of **9**
S46. HRESIMS $[M-H]^-$ of **9**
S47. ^{1}H NMR spectrum (500MHz, CD_2Cl_2) of **10**
S48. ^{13}C NMR spectrum (125MHz, CD_2Cl_2) of **10**
S49. HRESIMS $[M+H]^+$ of **10**
S50. HRESIMS $[M-H]^-$ of **10**
S51. Crystal data and structure refinement for Compound **8**
S52. Atomic coordinates (x 104) and equivalent isotropic displacement parameters (Å2x 103) for Compound **8** . U(eq) is defined as one third of the trace of the orthogonalized Uij tensor.
S53. Bond lengths [Å] and angles [°] for Compound **8**
S54. Anisotropic displacement parameters (Å2x 103) for Compound **8** . The anisotropic displacement factor exponent takes the form: -2p2[h2 a*2U11 + ... + 2 h k a* b* U12]
S55. Hydrogen coordinates (x 104) and isotropic displacement parameters (Å2x 10 3) for Compound **8**
S56. Torsion angles [°] for Compound **8**
S57. Hydrogen bonds for Compound **8** [Å and °].
S58-S72 Crystallographic data for compounds **7** and **10**

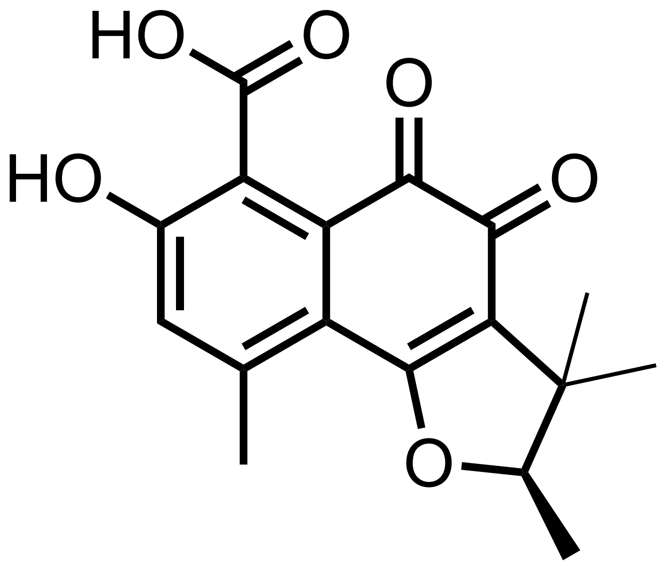

Peniciphenalenin D (**1**)

$[\alpha]_D$: +119.70° (c 0.10, MeOH)

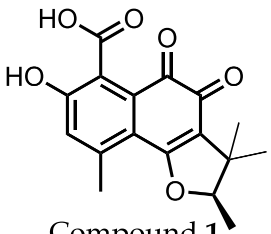

Compound **1**

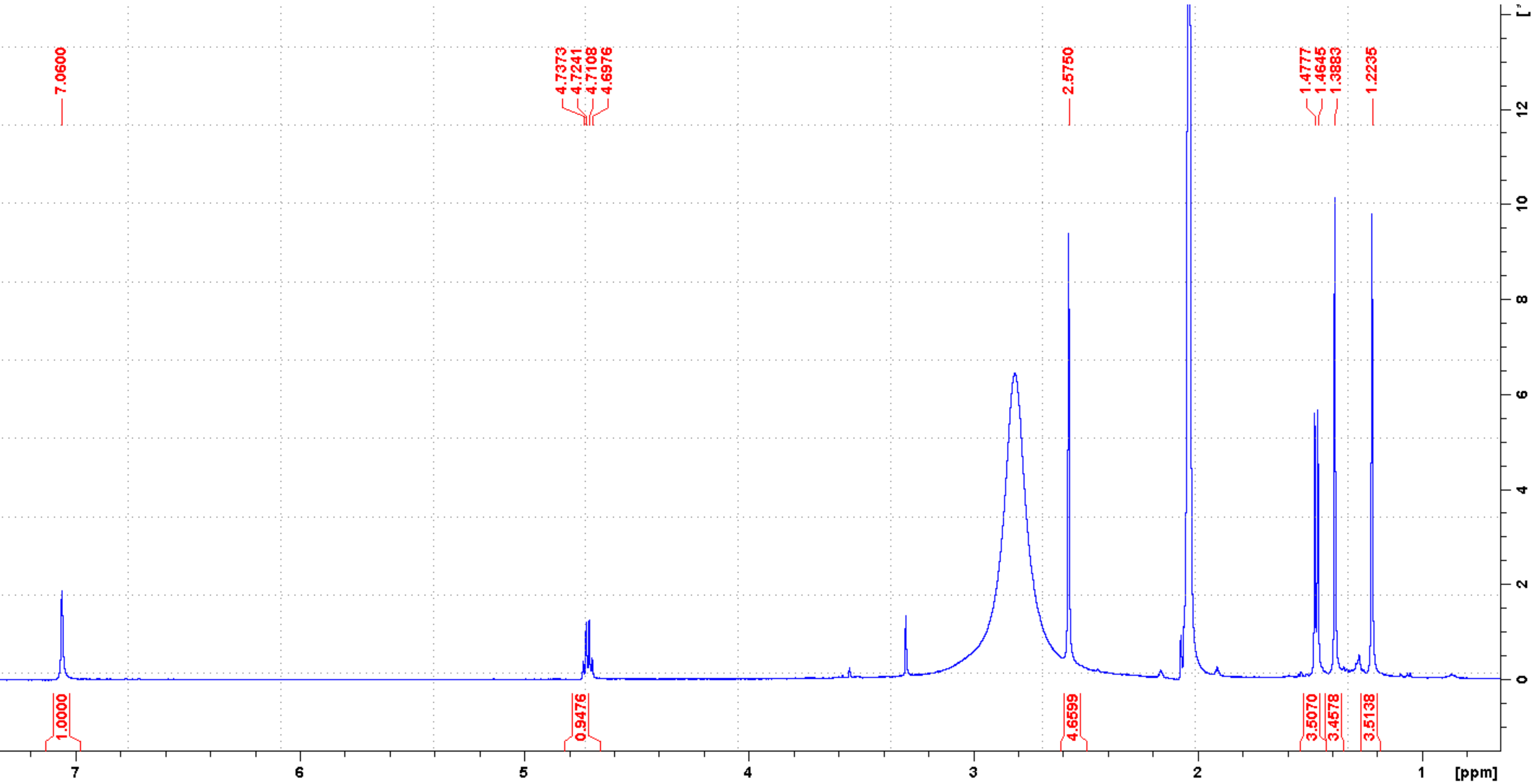

S1. ^{1}H NMR spectrum (500MHz, Acetone-d_6) of **1**

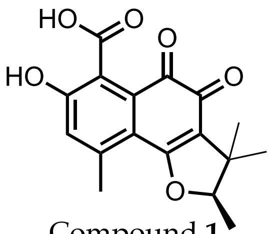

Compound **1**

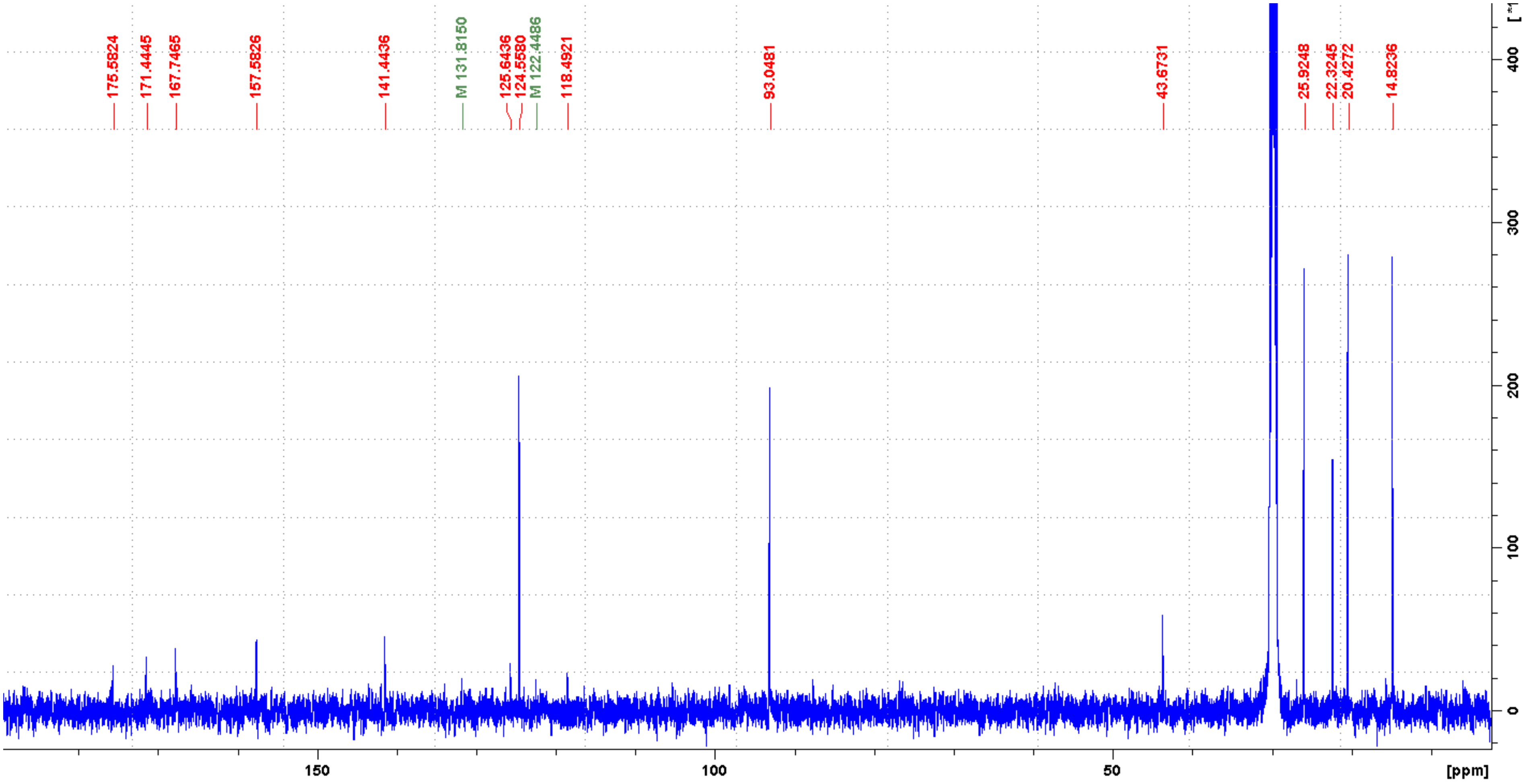

S2. ^{13}C NMR spectrum (125MHz, Acetone-d6) of **1**

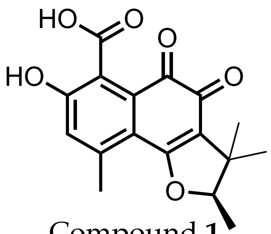

Compound **1**

Elemental Composition Report

Page 1

Single Mass Analysis

Tolerance = 10.0 PPM / DBE: min = -1.5, max = 100.0
Element prediction: Off
Number of isotope peaks used for i-FIT = 9

Monoisotopic Mass, Even Electron Ions
58 formula(e) evaluated with 1 results within limits (all results (up to 1000) for each mass)
Elements Used:
C: 0-50 H: 0-100 O: 0-20

08-Aug-2019 4::2::6 LCT Premier OUAZZANI_arcile173-1 21 (0.573) Cm (18:24-30:70x2.000)
1: TOF MS ES+

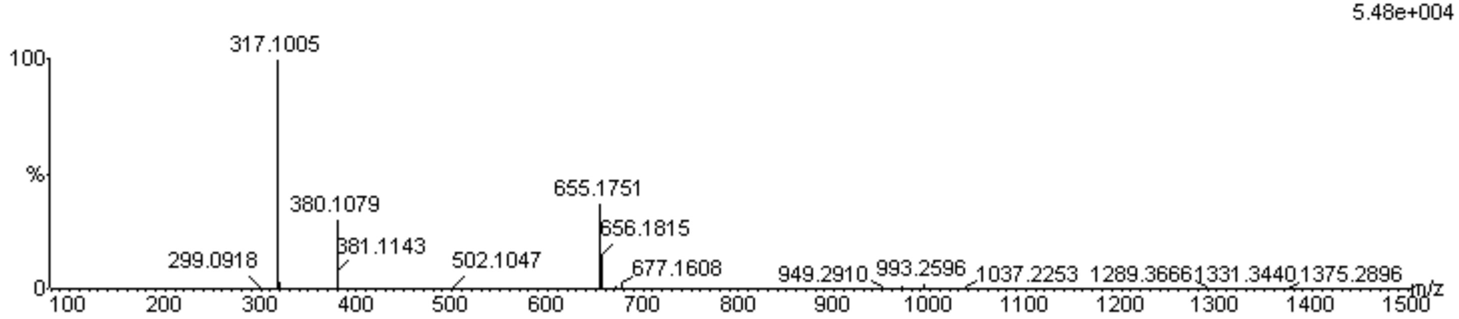

Minimum:				-1.5			
Maximum:		5.0	10.0	100.0			
Mass	Calc. Mass	mDa	PPM	DBE	i-FIT	i-FIT (Norm)	Formula
317.1005	317.1025	-2.0	-6.3	9.5	573.5	0.0	C17 H17 O6

S3. HRESIMS $[M+H]^+$ of **1**

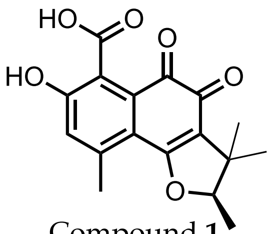

Compound **1**

Elemental Composition Report

Page 1

Single Mass Analysis

Tolerance = 10.0 PPM / DBE: min = -1.5, max = 100.0
Element prediction: Off
Number of isotope peaks used for i-FIT = 9

Monoisotopic Mass, Even Electron Ions
61 formula(e) evaluated with 1 results within limits (all results (up to 1000) for each mass)
Elements Used:
C: 0-50 H: 0-100 O: 0-20

08-Aug-2019 4::2::6 LCT Premier OUAZZANI_arcile173-1 19 (0.526) Cm (18:23-33:68x2.000)
2: TOF MS ES-

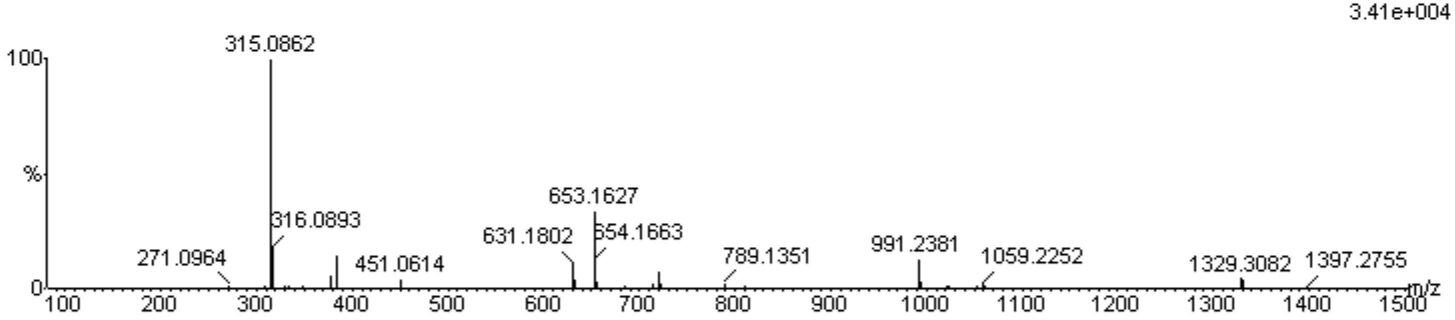

Minimum:				-1.5			
Maximum:		5.0	10.0	100.0			
Mass	Calc. Mass	mDa	PPM	DBE	i-FIT	i-FIT (Norm)	Formula
315.0862	315.0869	-0.7	-2.2	10.5	321.2	0.0	C17 H15 O6

S4. HRESIMS [M-H]- of **1**

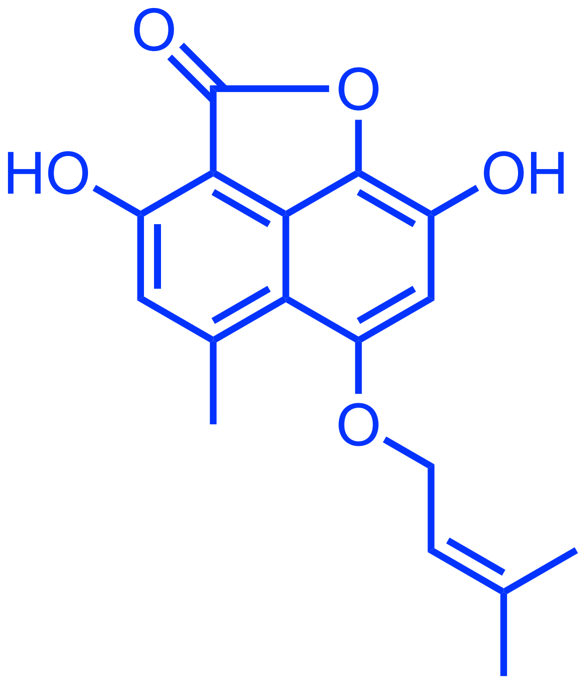

Compound **2** (New)

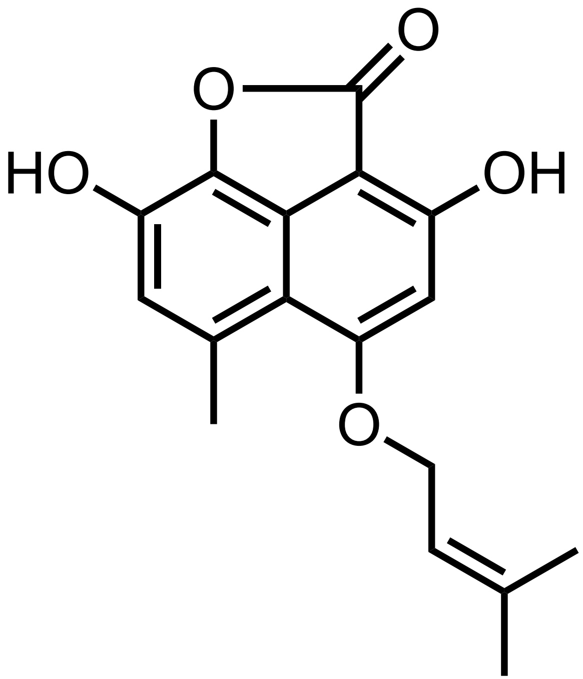

Coniolactone (**3**)

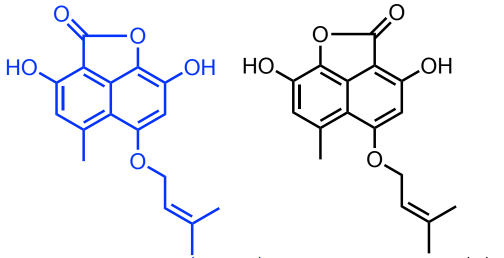

Compound **2** (New) Coniolactone (**3**)

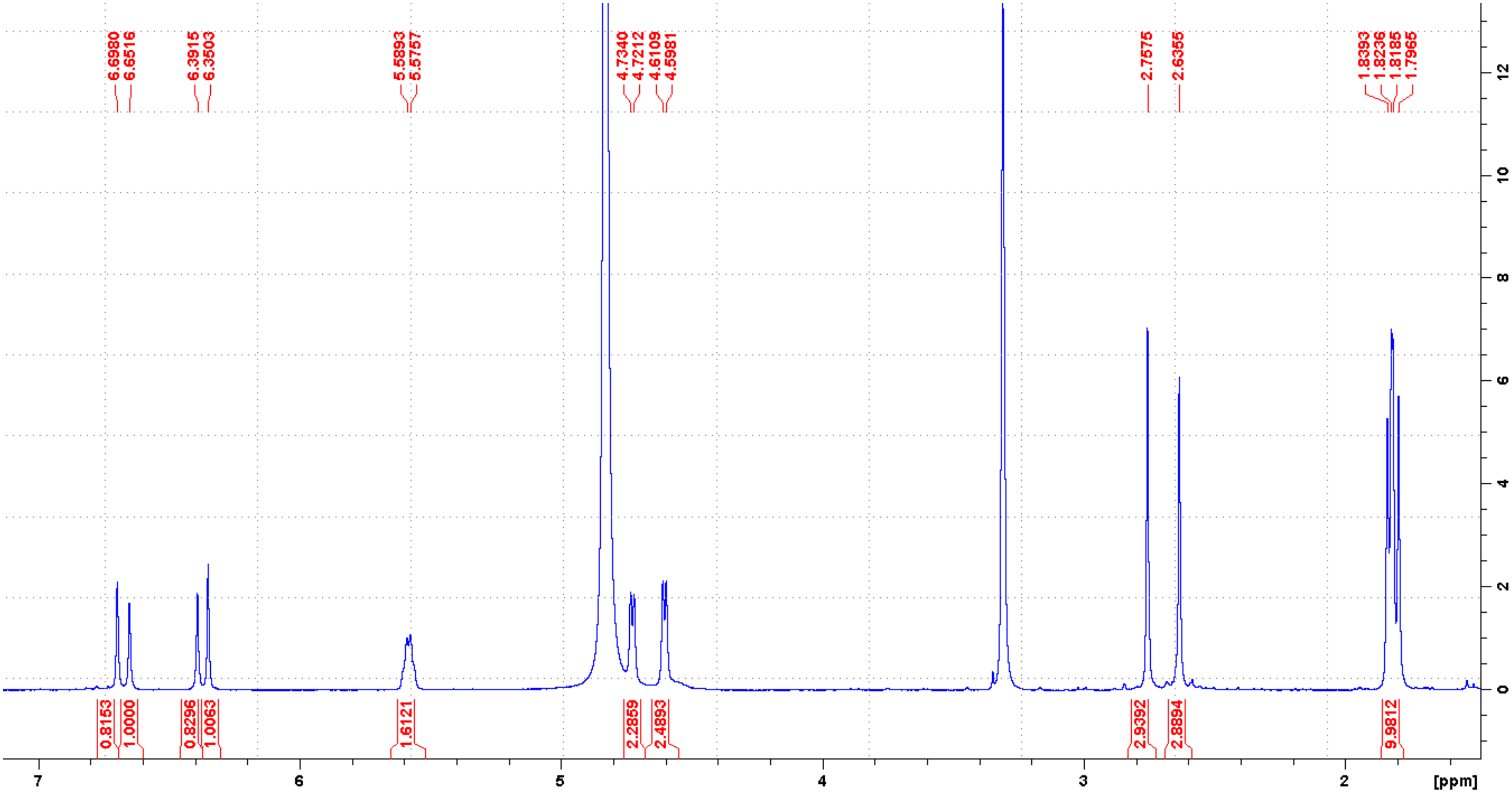

S5. ^{1}H NMR spectrum (500MHz, MeOD) of **2** + **3**

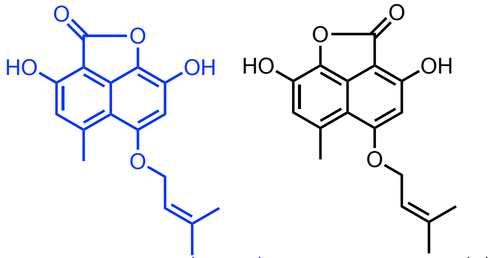

Compound **2** (New) Coniolactone (**3**)

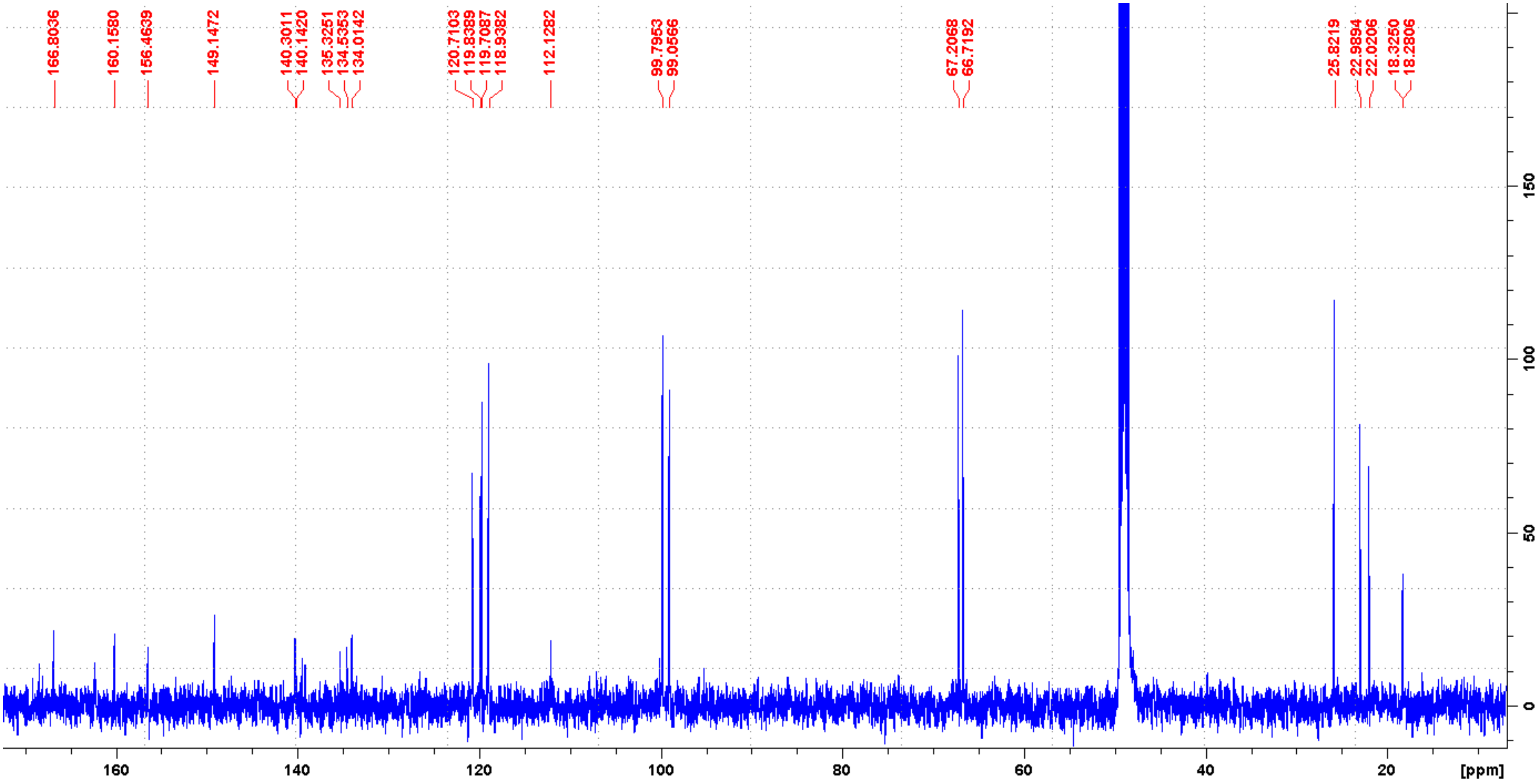

S6. ^{13}C NMR spectrum (125MHz, MeOD) of **2** + **3**

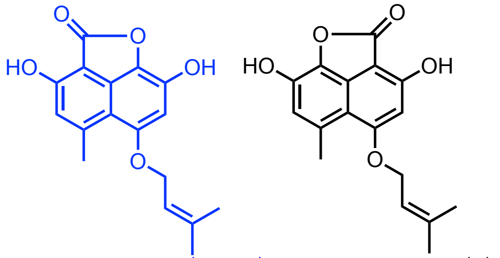

Compound **2** (New) Coniolactone (**3**)

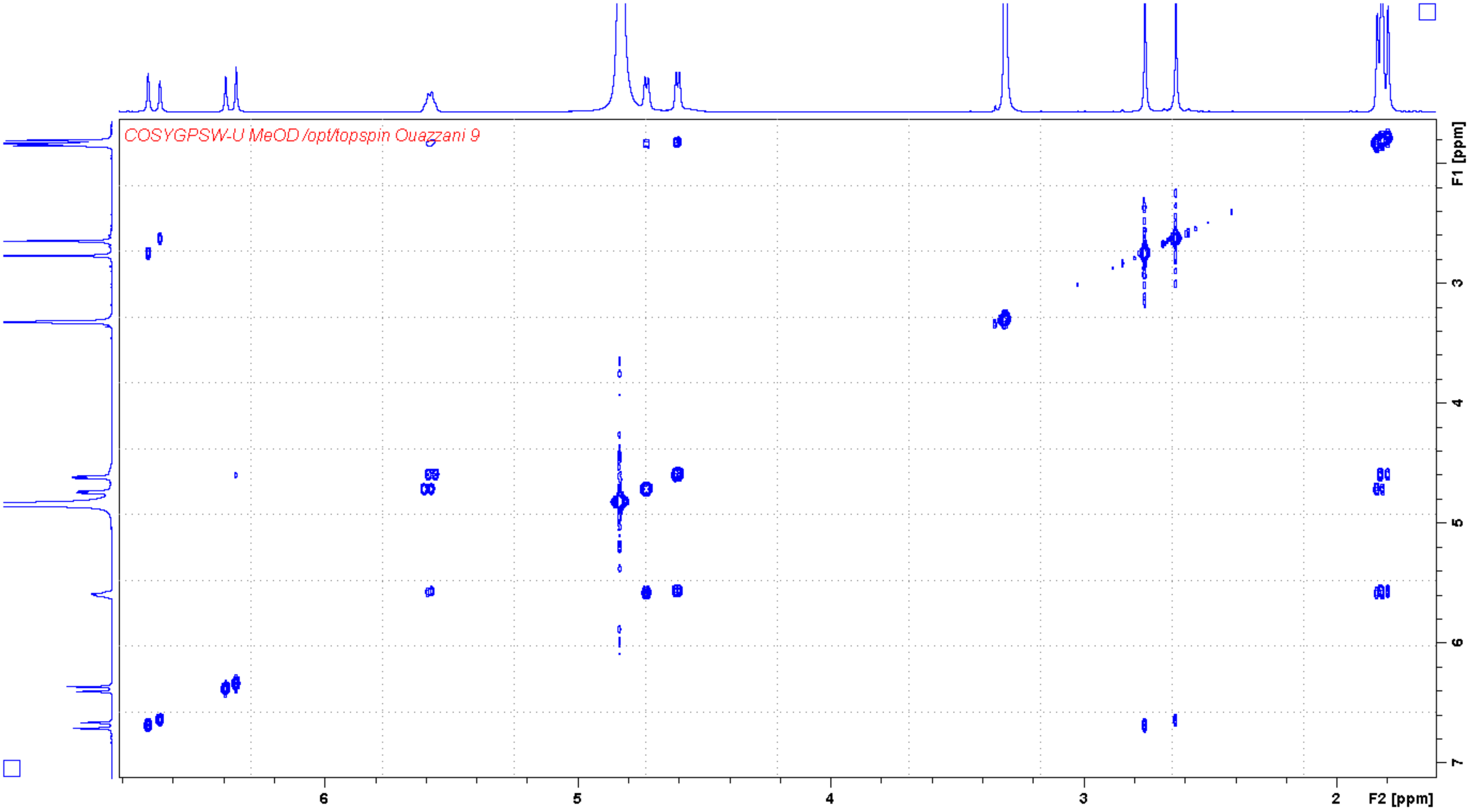

S7. ^{1}H-^{1}H COSY NMR spectrum (500MHz, MeOD) of **2** + **3**

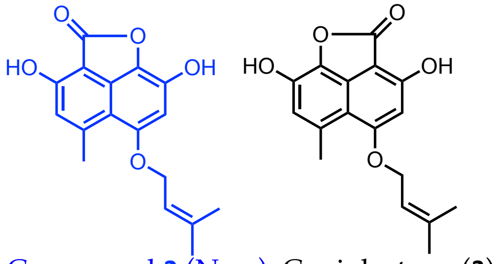

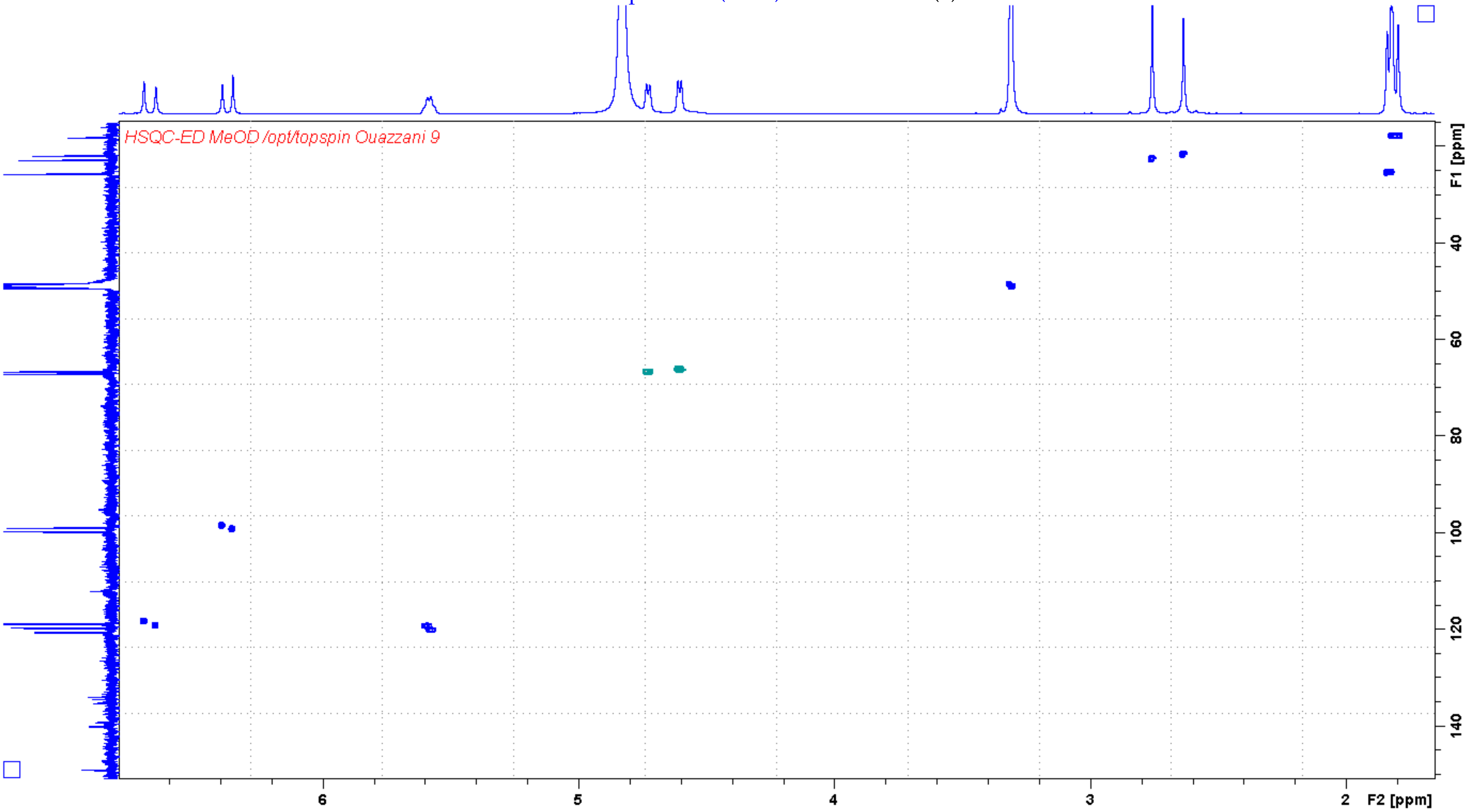

S8. ^{1}H-^{13}C HSQC spectrum (500 MHz, MeOH) of **2** + **3**

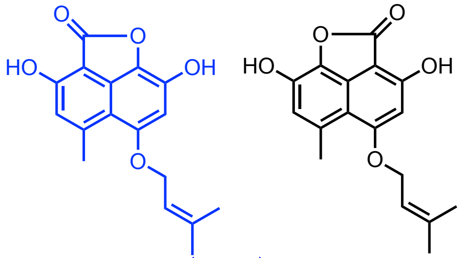

Compound **2** (New) Coniolactone (**3**)

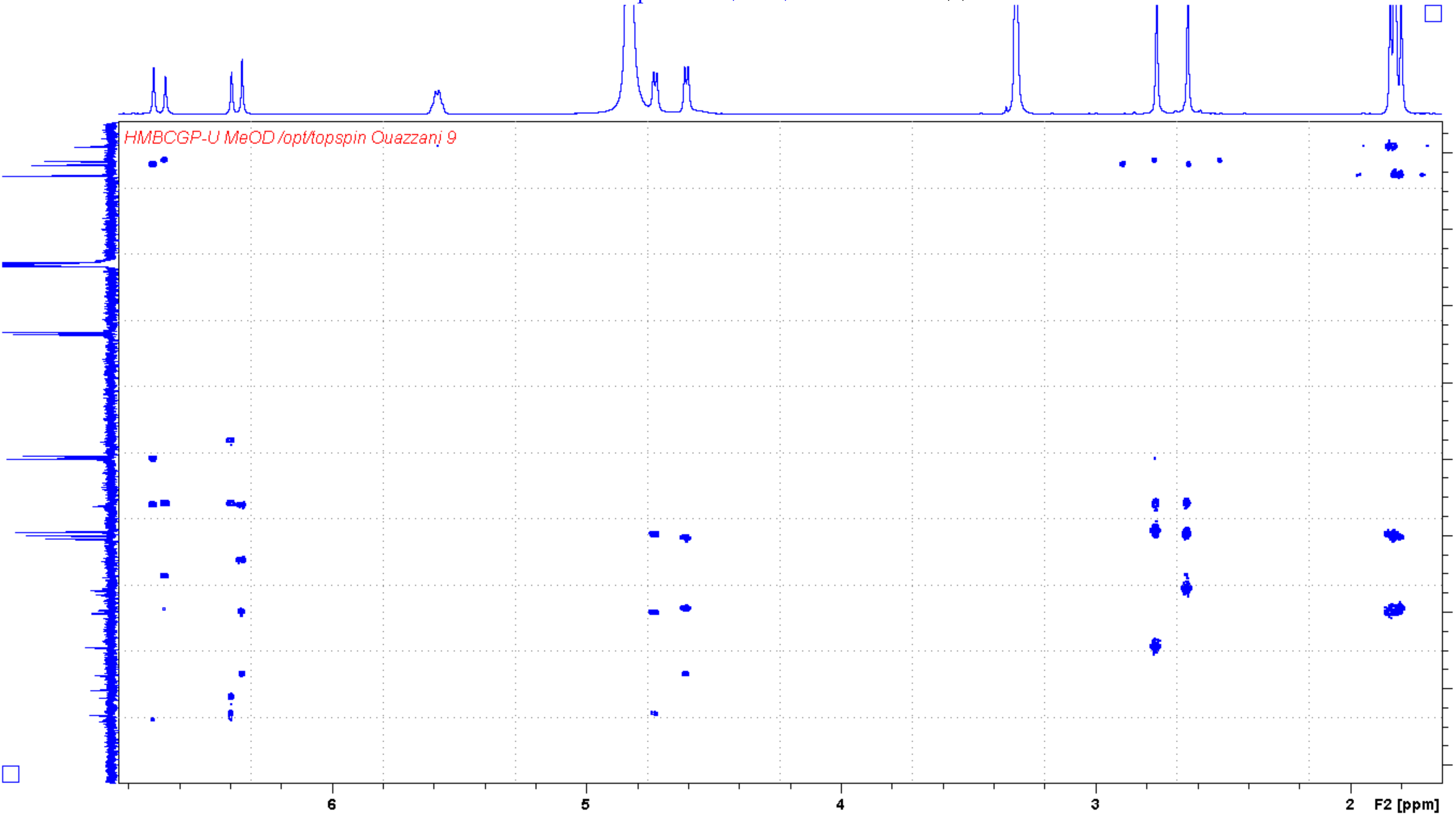

S9. ^{1}H-^{13}C HMBC spectrum (500 MHz, MeOH) of **2** + **3**

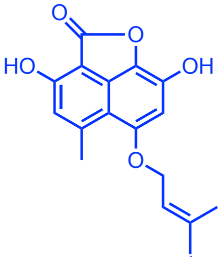

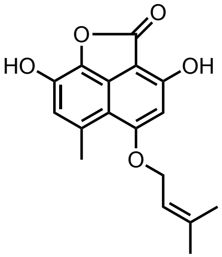

Compound **2** (New) Coniolactone (**3**)

Elemental Composition Report **Page 1**

Single Mass Analysis
Tolerance = 10.0 PPM / DBE: min = -1.5, max = 100.0
Element prediction: Off
Number of isotope peaks used for i-FIT = 9

Monoisotopic Mass, Even Electron Ions
463 formula(e) evaluated with 3 results within limits (all results (up to 1000) for each mass)
Elements Used:
C: 0-50 H: 0-100 N: 0-10 O: 0-20

06-May-2019 16:10:57 LCT Premier OUAZZANI_glegoff108-1 20 (0.534) Cm (17:24-(30:64+3:12)x2.000)
1: TOF MS ES+

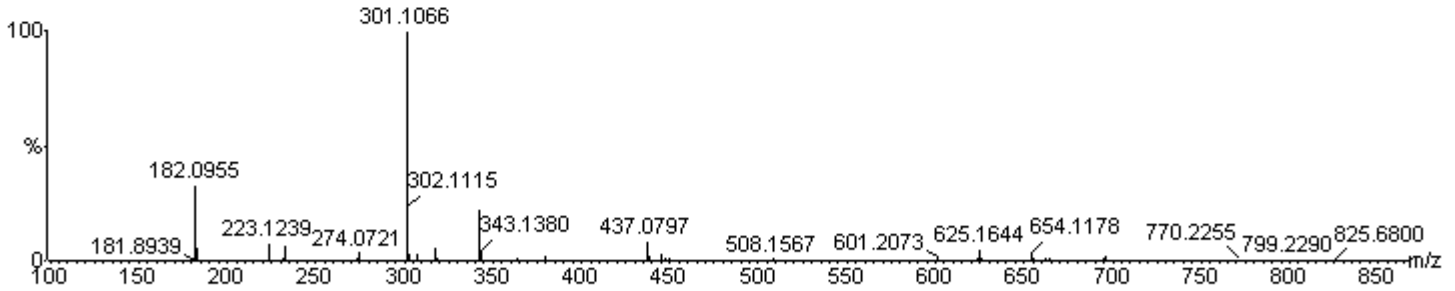

Minimum:				-1.5			
Maximum:		5.0	10.0	100.0			
Mass	Calc. Mass	mDa	PPM	DBE	i-FIT	i-FIT (Norm)	Formula
301.1066	301.1076	-1.0	-3.3	9.5	422.8	1.7	C17 H17 O5
	301.1049	1.7	5.6	10.5	423.6	2.5	C13 H13 N6 O3
	301.1089	-2.3	-7.6	14.5	421.4	0.3	C18 H13 N4 O

S10. HRESIMS $[M+H]^+$ of **2** + **3**

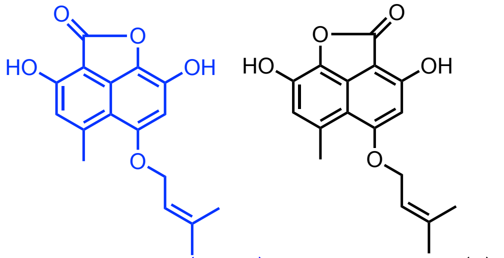

Compound 2 (New) Coniolactone (**3**)

Elemental Composition Report

Page 1

Single Mass Analysis

Tolerance = 10.0 PPM / DBE: min = -1.5, max = 100.0
Element prediction: Off
Number of isotope peaks used for i-FIT = 9

Monoisotopic Mass, Even Electron Ions
458 formula(e) evaluated with 4 results within limits (all results (up to 1000) for each mass)
Elements Used:
C: 0-50 H: 0-100 N: 0-10 O: 0-20

06-May-2019 16:10:57 LCT Premier OUAZZANI_glegoff108-1 22 (0.598) Cm (17:24-(3:10+34:70)x2.000)
2: TOF MS ES-

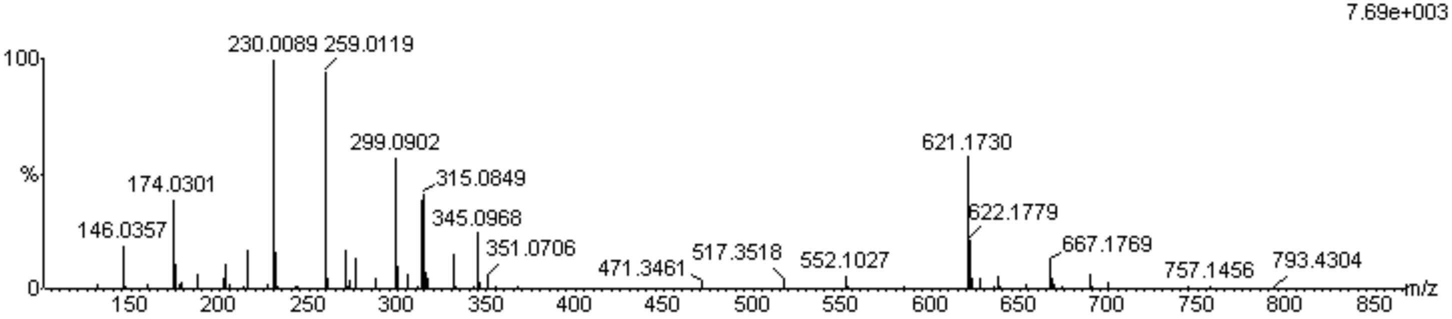

Minimum:				-1.5			
Maximum:		5.0	10.0	100.0			
Mass	Calc. Mass	mDa	PPM	DBE	i-FIT	i-FIT (Norm)	Formula
299.0902	299.0893	0.9	3.0	11.5	41.4	0.0	C13 H11 N6 O3
	299.0911	-0.9	-3.0	-1.5	51.2	9.8	C H15 N8 O10
	299.0919	-1.7	-5.7	10.5	46.2	4.8	C17 H15 O5
	299.0879	2.3	7.7	6.5	45.3	3.9	C12 H15 N2 O7

S11. HRESIMS [M-H]$^-$ of **2** + **3**

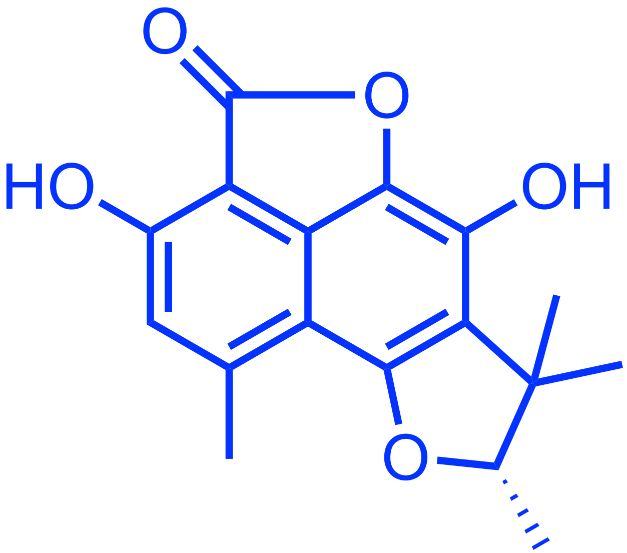

(-) Peniciphenalenin F (**4**)

$[\alpha]_D$: -36.10° (c 0.10, MeOH)

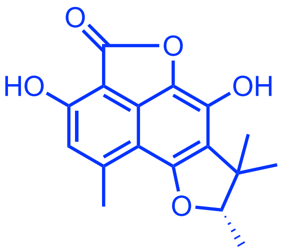

(-) Peniciphenalenin F (4)

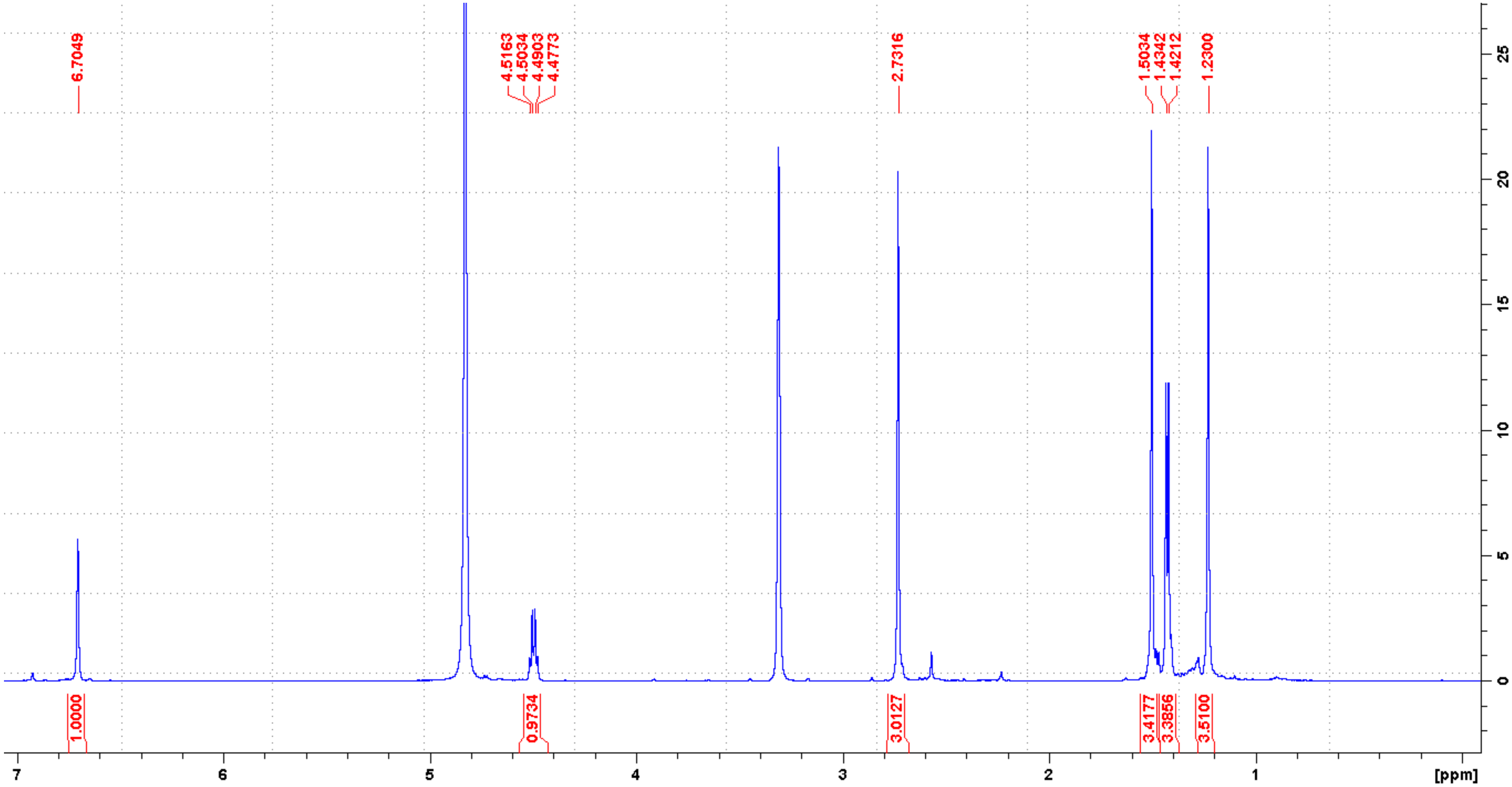

S12. ^{1}H NMR spectrum (500MHz, MeOD) of **4**

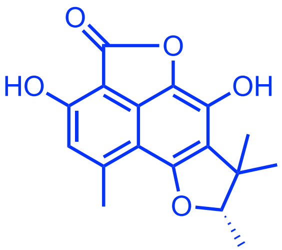

(-) Peniciphenalenin F (**4**)

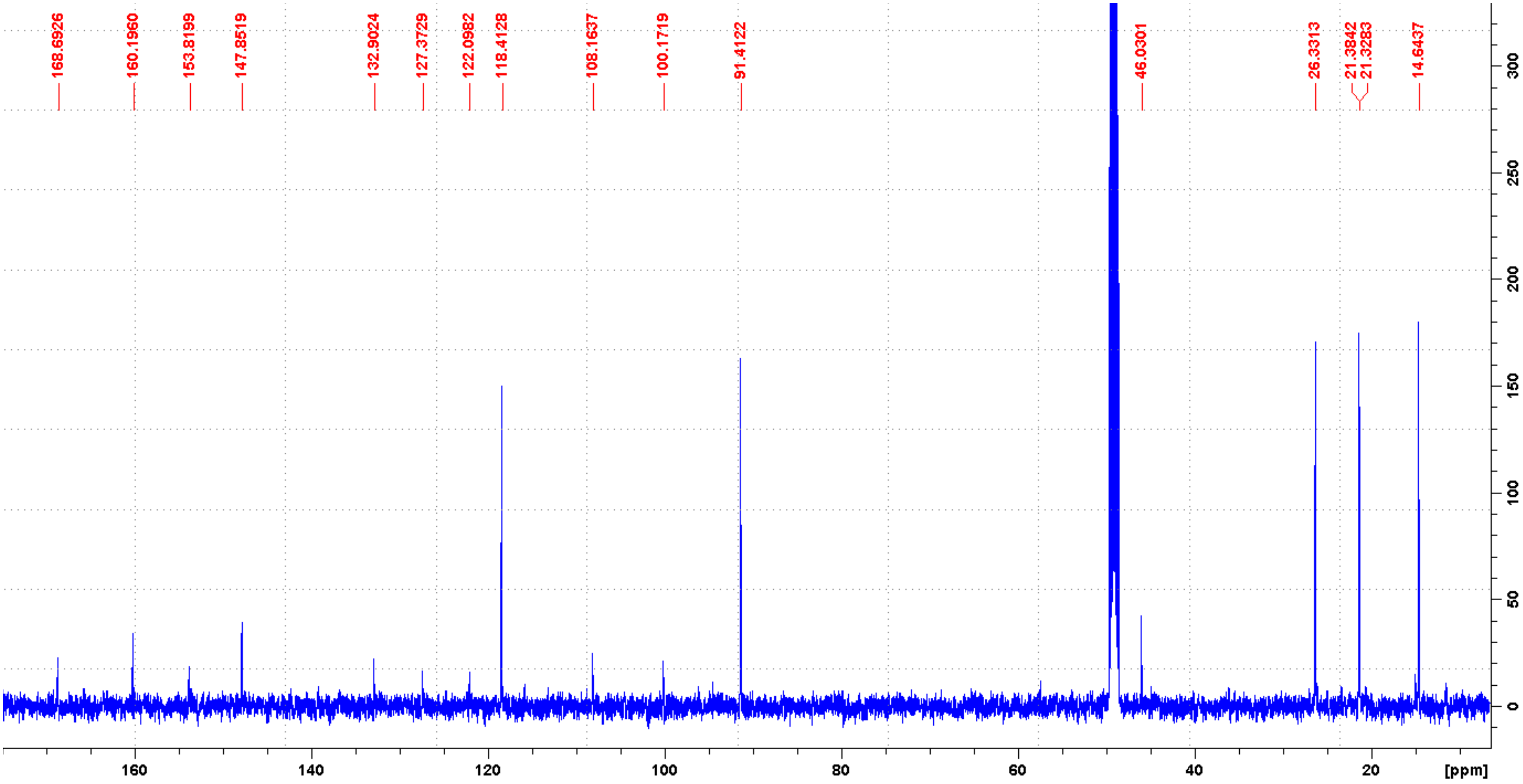

S13. ^{13}C NMR spectrum (125MHz, MeOD) of **4**

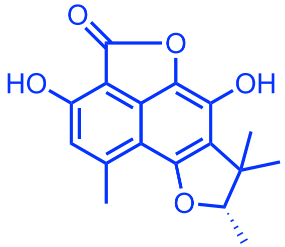

(-) Peniciphenalenin F (4)

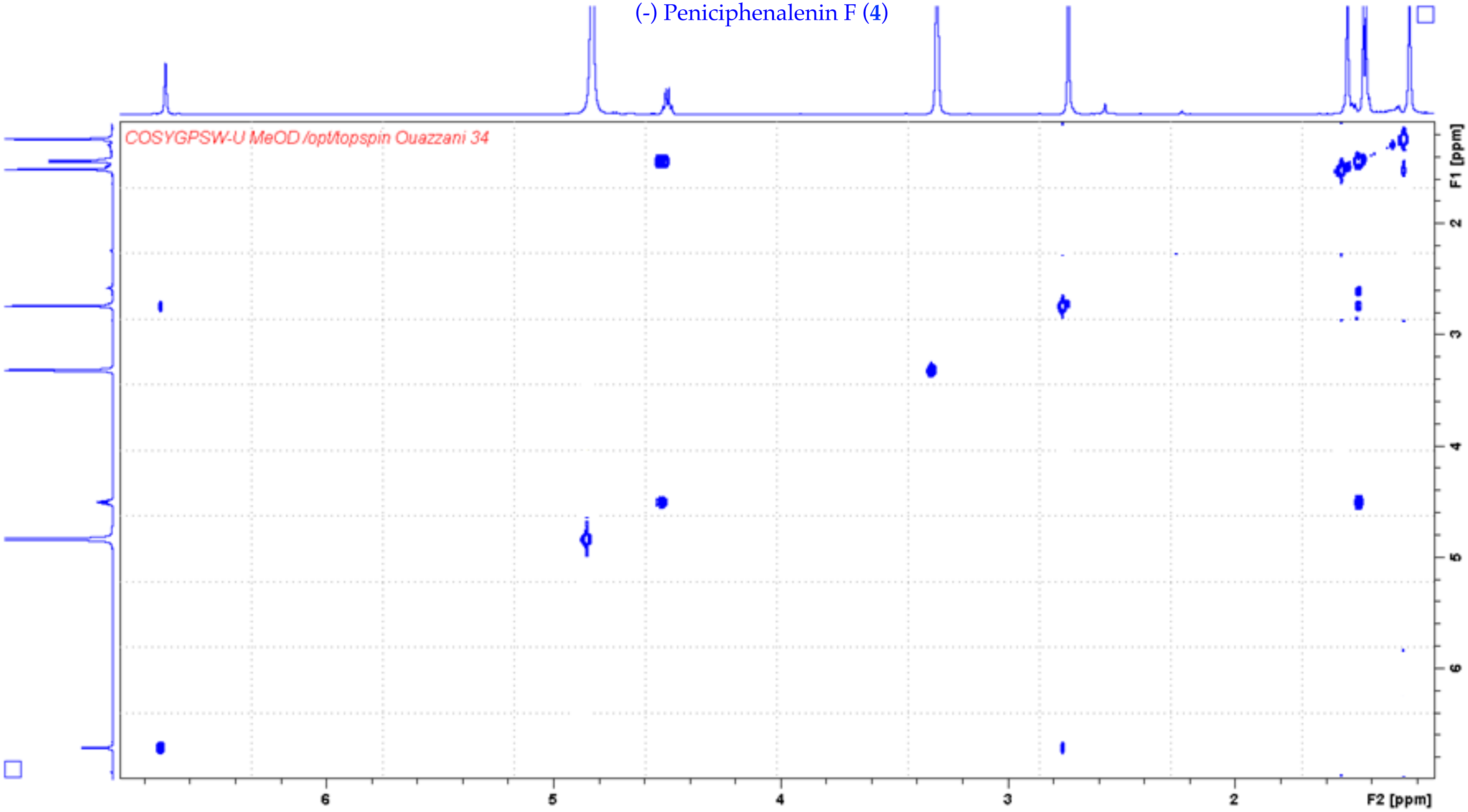

S14. ${}^1H$-${}^1H$ COSY NMR spectrum (500MHz, MeOD) of **4**

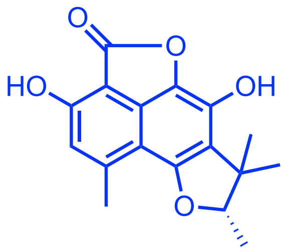

(-) Peniciphenalenin F (4)

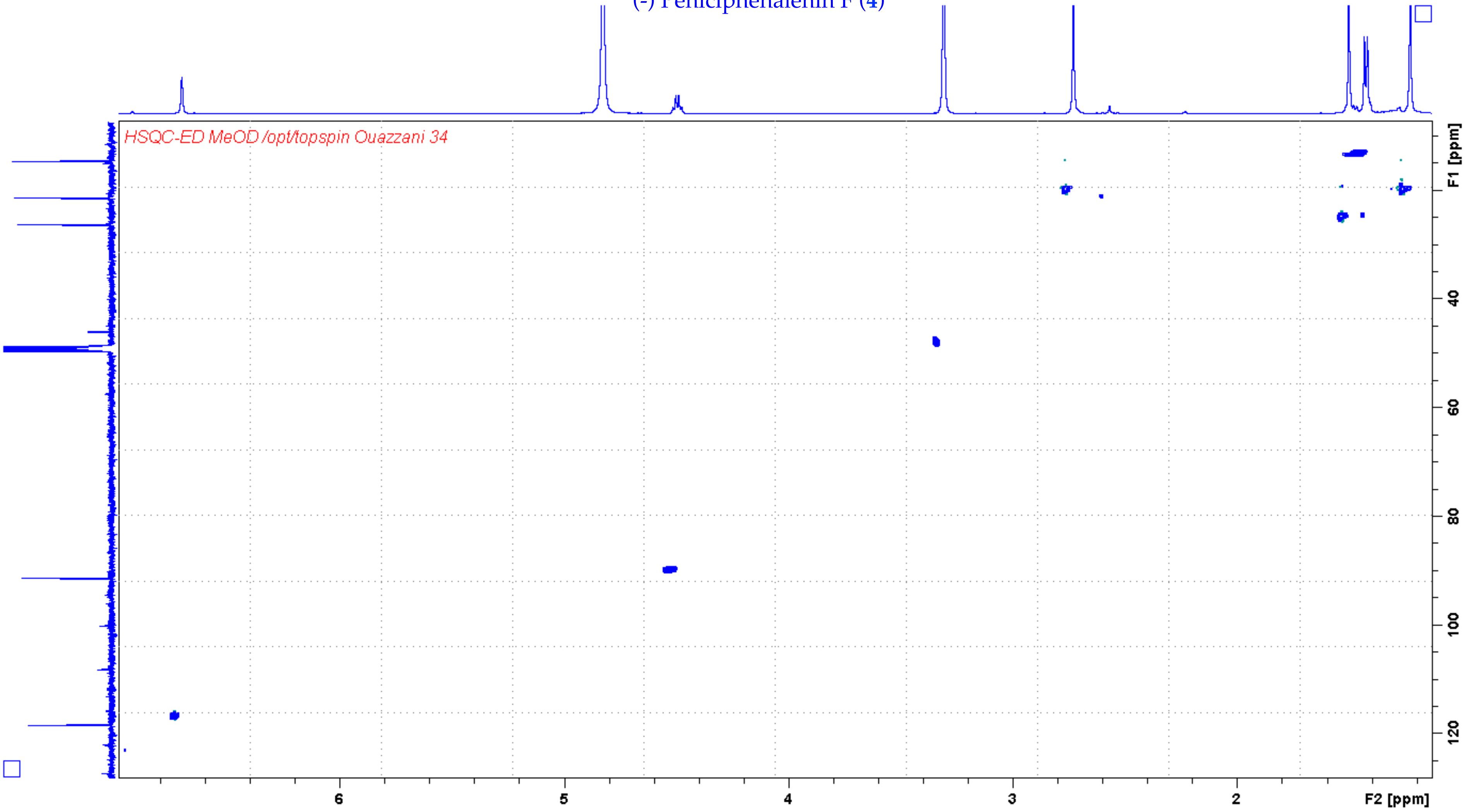

S15. ^{1}H-^{13}C HSQC spectrum (500 MHz, MeOH) of **4**

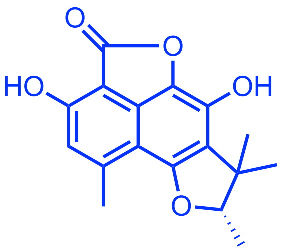

(-) Peniciphenalenin F (4)

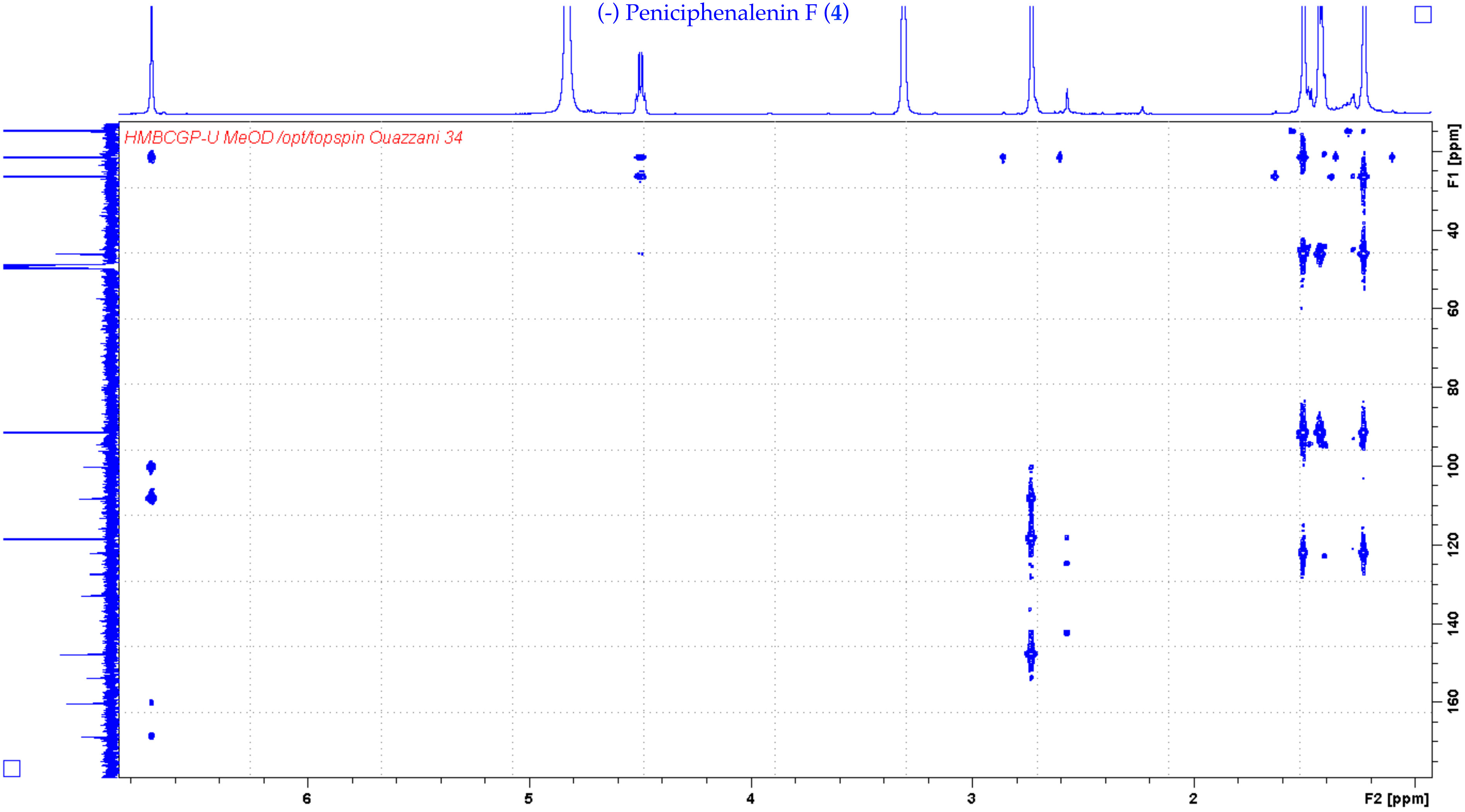

S16. ^{1}H-^{13}C HMBC spectrum (500 MHz, MeOH) of **4**

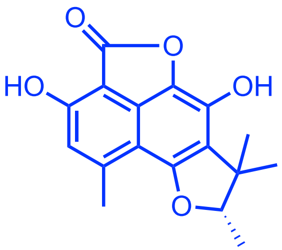

(-) Peniciphenalenin F (4)

Elemental Composition Report **Page 1**

Single Mass Analysis
Tolerance = 10.0 PPM / DBE: min = -1.5, max = 100.0
Element prediction: Off
Number of isotope peaks used for i-FIT = 9

Monoisotopic Mass, Even Electron Ions
463 formula(e) evaluated with 3 results within limits (all results (up to 1000) for each mass)
Elements Used:
C: 0-50 H: 0-100 N: 0-10 O: 0-20

06-May-2019 16:15:00 LCT Premier OUAZZANI_glegoff108-2 20 (0.532) Cm (17:24-33:70x2.000)
1: TOF MS ES+

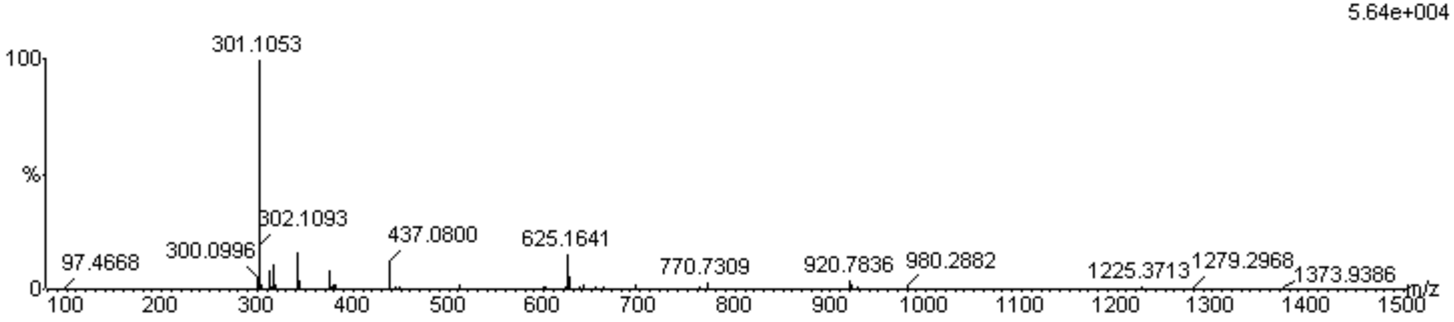

		mDa	PPM	DBE			
Minimum:				-1.5			
Maximum:		5.0	10.0	100.0			
Mass	Calc. Mass	mDa	PPM	DBE	i-FIT	i-FIT (Norm)	Formula
301.1053	301.1049	0.4	1.3	10.5	675.7	4.5	C13 H13 N6 O3
	301.1036	1.7	5.6	5.5	681.5	10.3	C12 H17 N2 O7
	301.1076	-2.3	-7.6	9.5	671.2	0.0	C17 H17 O5

S17. HRESIMS $[M+H]^+$ of **4**

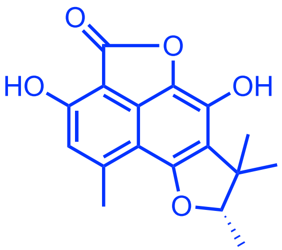

(-) Peniciphenalenin F (**4**)

Elemental Composition Report **Page 1**

Single Mass Analysis
Tolerance = 10.0 PPM / DBE: min = -1.5, max = 100.0
Element prediction: Off
Number of isotope peaks used for i-FIT = 9

Monoisotopic Mass, Even Electron Ions
458 formula(e) evaluated with 4 results within limits (all results (up to 1000) for each mass)
Elements Used:
C: 0-50 H: 0-100 N: 0-10 O: 0-20
06-May-2019 16:15:00 LCT Premier OUAZZANI_glegoff108-2 22 (0.596) Cm (17:24-37:68x2.000)
2: TOF MS ES-

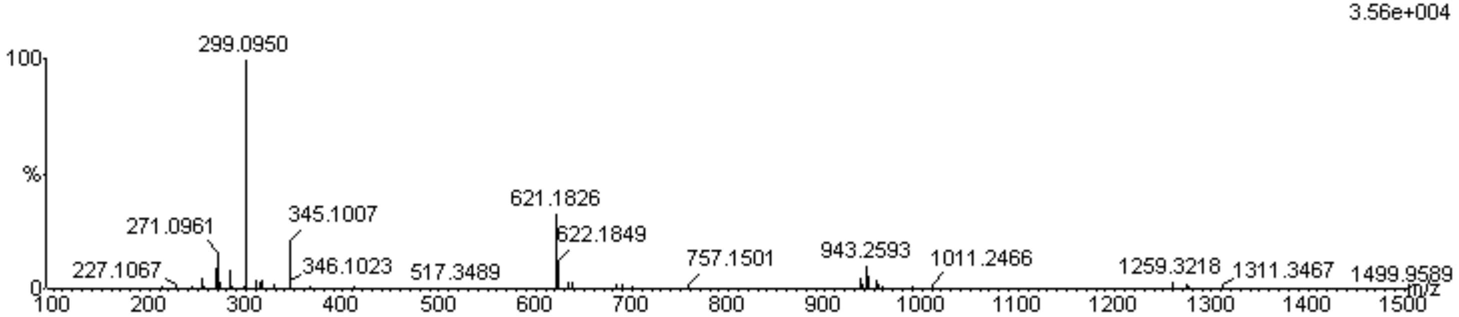

Minimum:				-1.5			
Maximum:		5.0	10.0	100.0			
Mass	Calc. Mass	mDa	PPM	DBE	i-FIT	i-FIT (Norm)	Formula
299.0950	299.0951	-0.1	-0.3	2.5	490.9	6.8	C6 H15 N6 O8
	299.0965	-1.5	-5.0	7.5	489.8	5.7	C7 H11 N10 O4
	299.0933	1.7	5.7	15.5	484.1	0.0	C18 H11 N4 O
	299.0978	-2.8	-9.4	1.5	488.9	4.8	C10 H19 O10

S18. HRESIMS [M-H]$^{-}$ of **4**

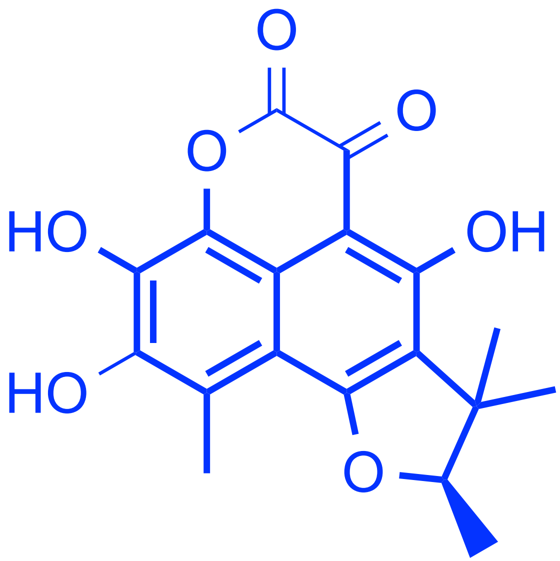

(+)-8-hydroxyscleroderolide (**5**)

$[\alpha]_D$: +65.01° (c 0.10, MeOH)

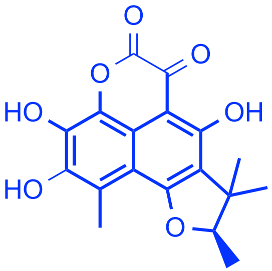

(+)-8-hydroxyscleroderolide (**5**)

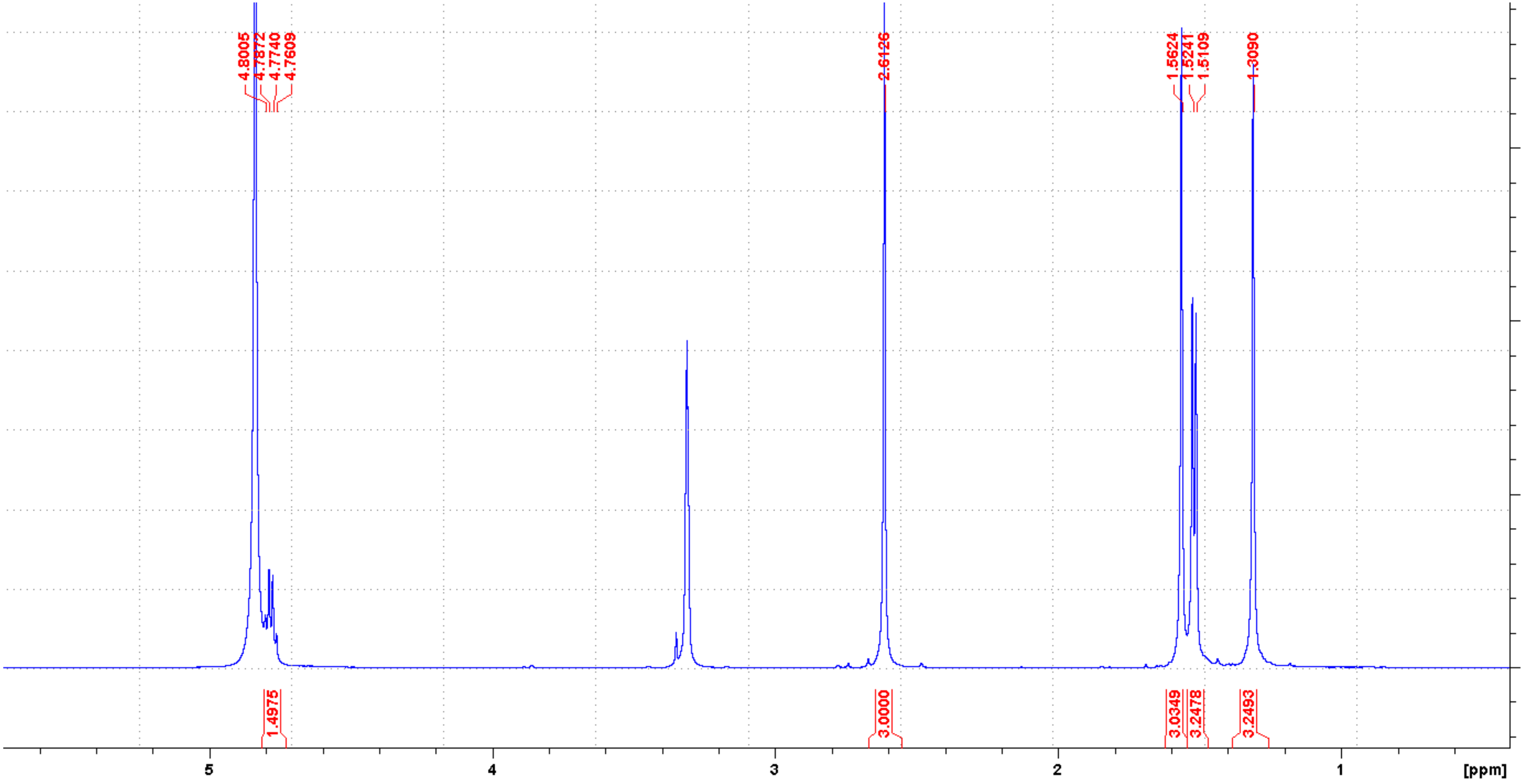

S19. ^{1}H NMR spectrum (500MHz, MeOD) of **5**

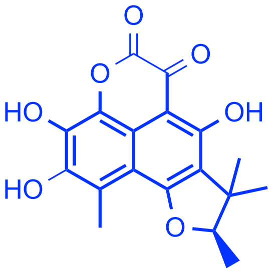

(+)-8-hydroxyscleroderolide (**5**)

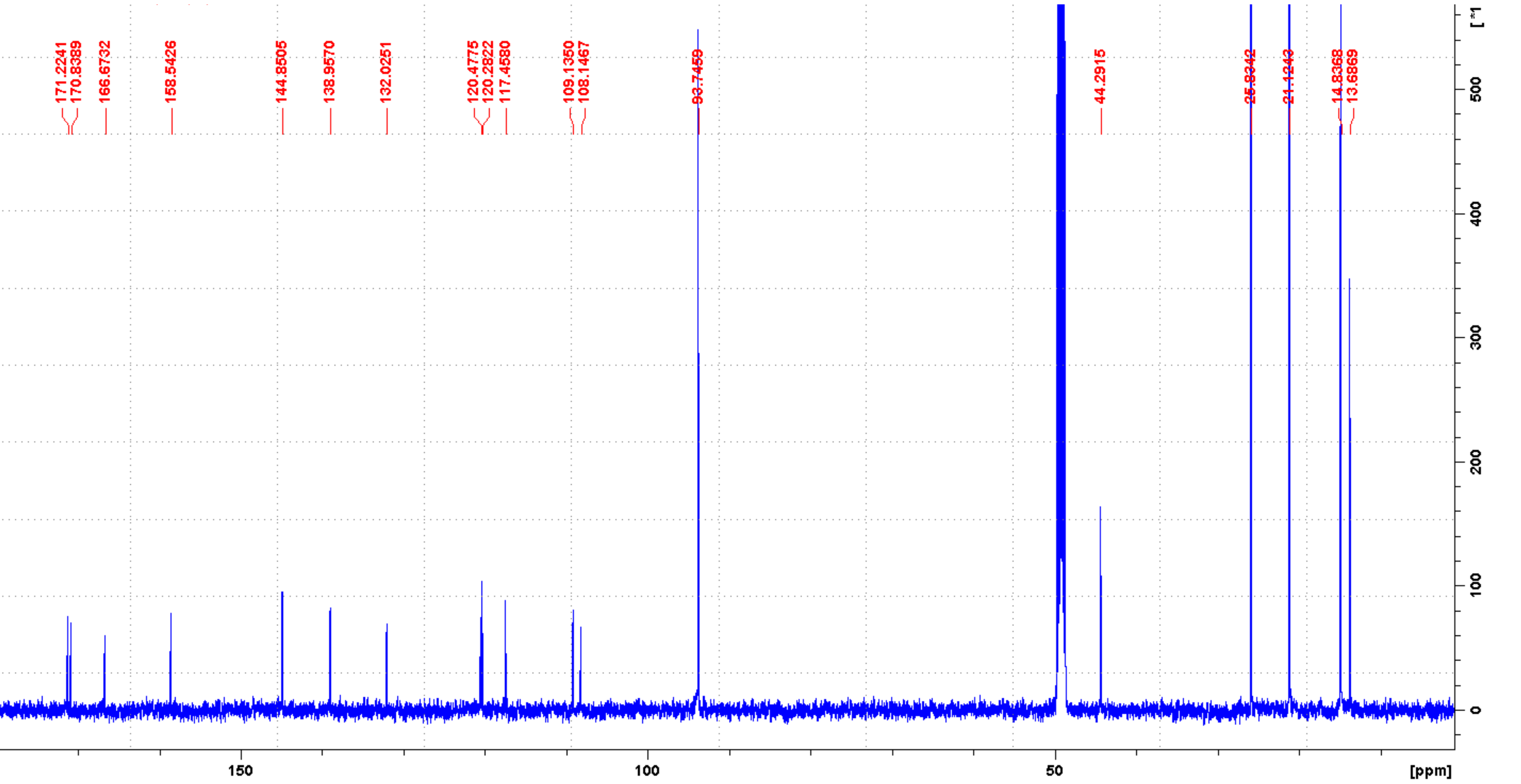

S20. ^{13}C NMR spectrum (125MHz, MeOD) of **5**

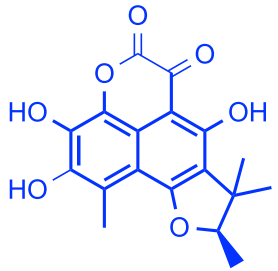

(+)-8-hydroxyscleroderolide (**5**)

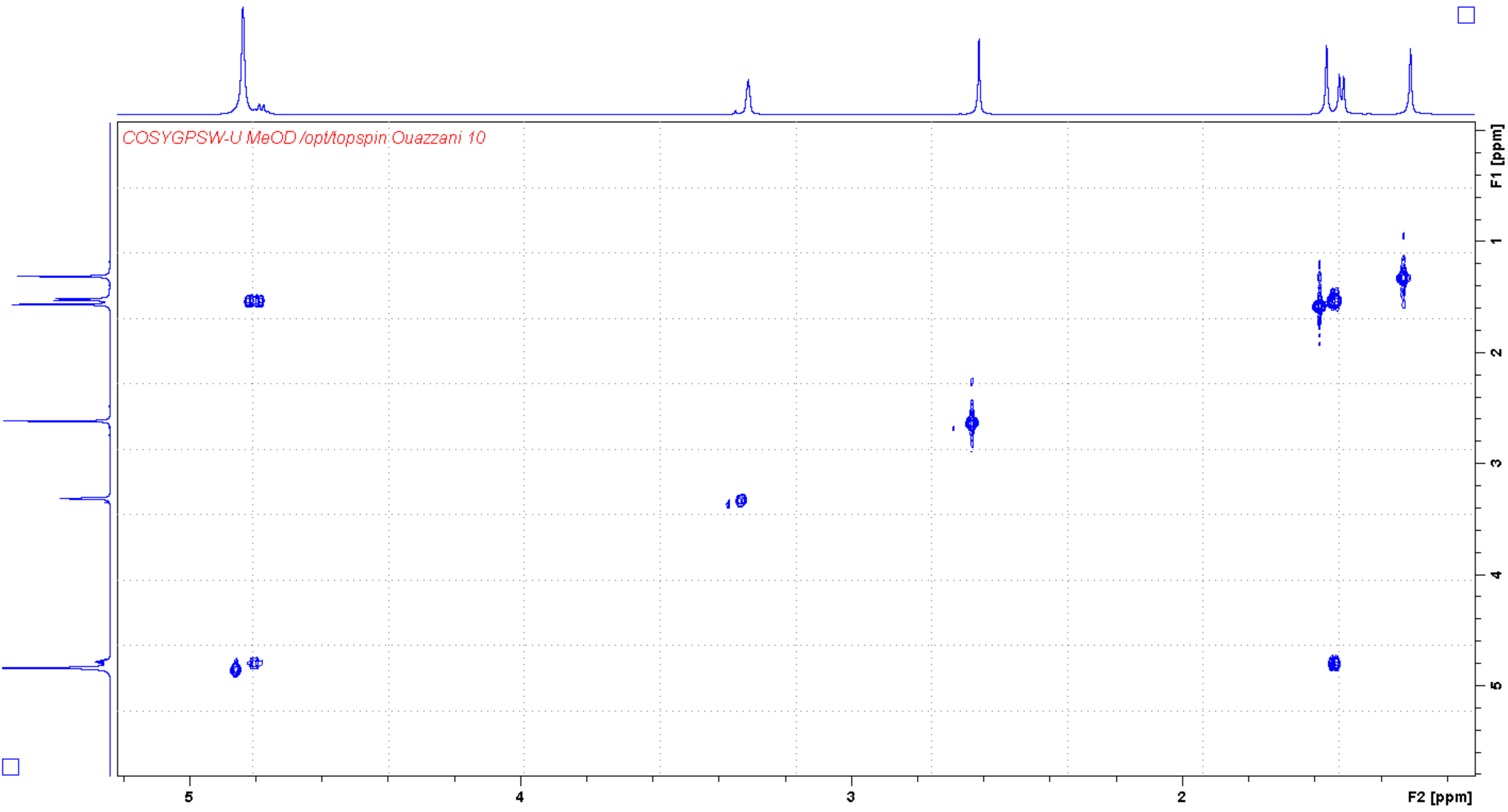

S21. ^1H-^1H COSY NMR spectrum (500MHz, MeOD) of **5**

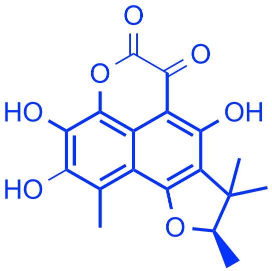

(+)-8-hydroxyscleroderolide (**5**)

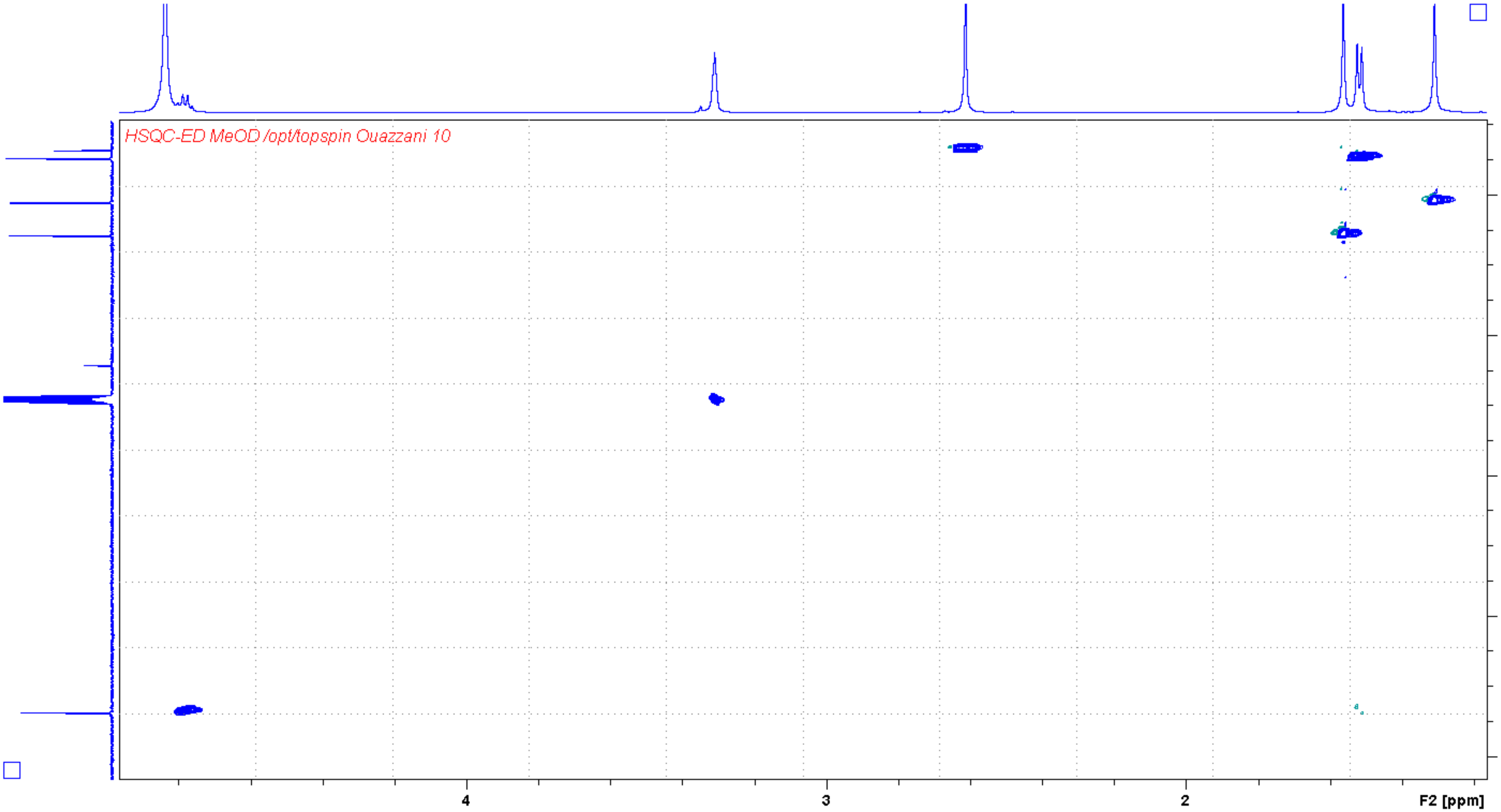

S22. ^{1}H-^{13}C HSQC spectrum (500 MHz, MeOH) of **5**

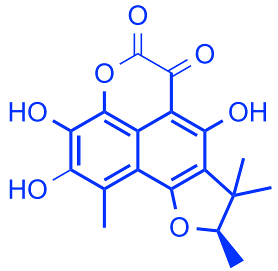

(+)-8-hydroxyscleroderolide (**5**)

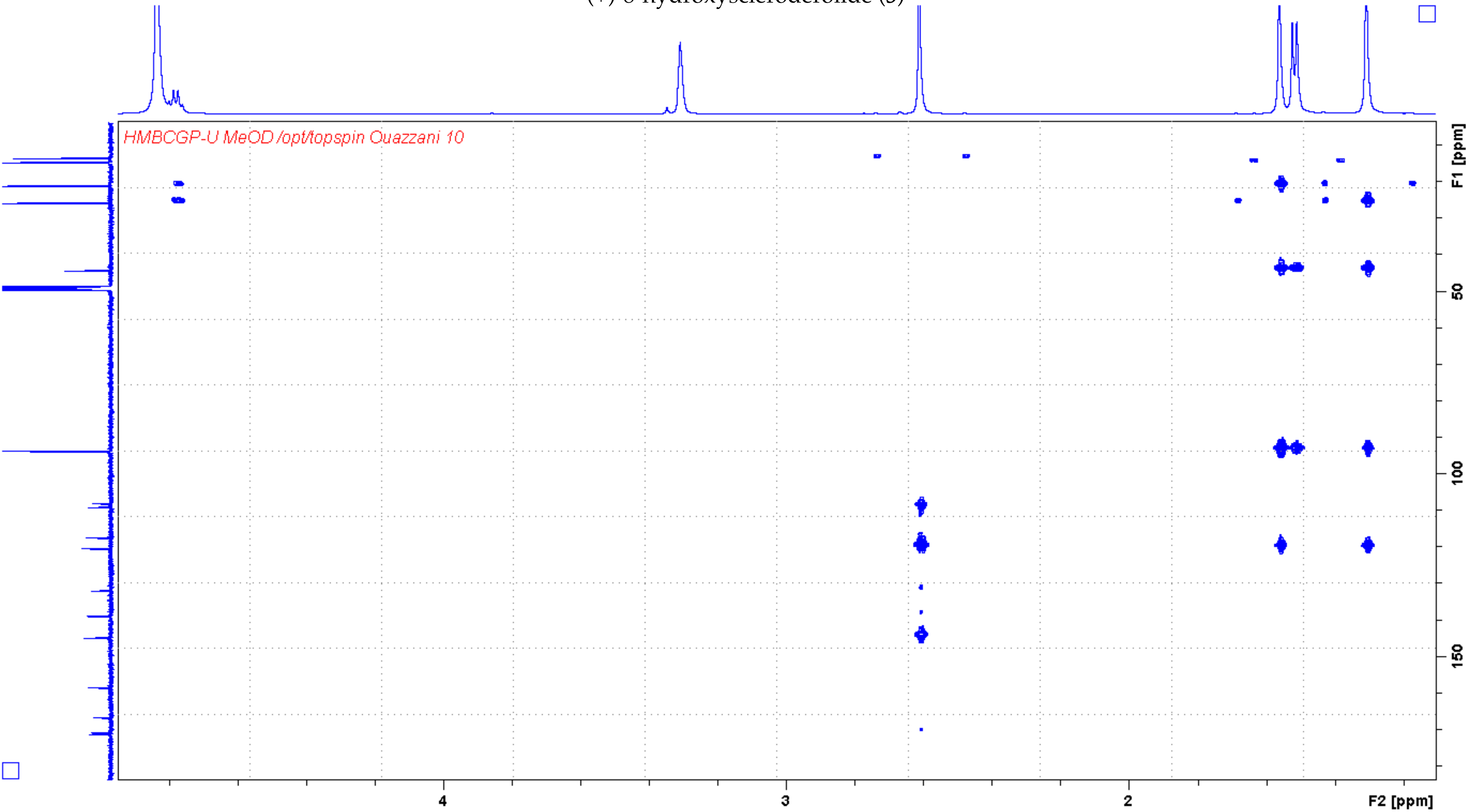

S23. ^{1}H-^{13}C HMBC spectrum (500 MHz, MeOH) of **5**

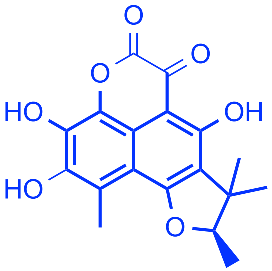

(+)-8-hydroxyscleroderolide (**5**)

Elemental Composition Report

Page 1

Single Mass Analysis

Tolerance = 14.0 PPM / DBE: min = -1.5, max = 100.0
Element prediction: Off
Number of isotope peaks used for i-FIT = 9

Monoisotopic Mass, Even Electron Ions
67 formula(e) evaluated with 1 results within limits (all results (up to 1000) for each mass)
Elements Used:
C: 0-70 H: 0-100 O: 0-50

13-Jun-2019 11:11:24 LCT Premier OUAZZANI_arcile166-2 22 (0.588) Cm (18:24)
1: TOF MS ES+

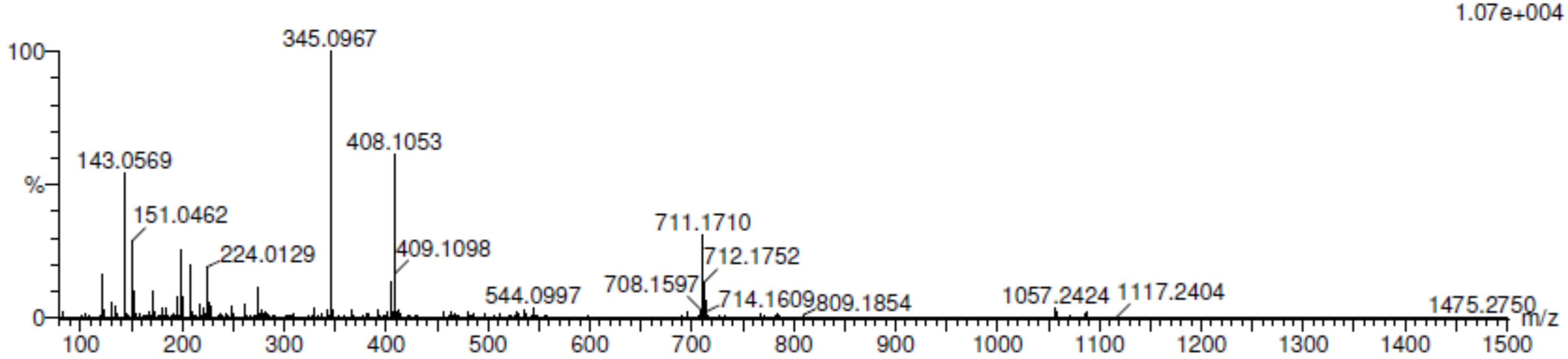

Minimum:				-1.5			
Maximum:		5.0	14.0	100.0			
Mass	Calc. Mass	mDa	PPM	DBE	i-FIT	i-FIT (Norm)	Formula
345.0967	345.0974	-0.7	-2.0	10.5	848.2	0.0	C18 H17 O7

S24. HRESIMS $[M+H]^+$ of **5**

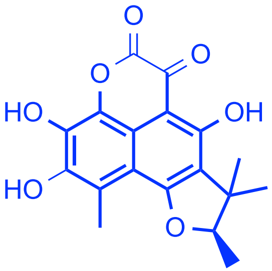

(+)-8-hydroxyscleroderolide (**5**)

Elemental Composition Report

Single Mass Analysis

Tolerance = 14.0 PPM / DBE: min = -1.5, max = 100.0
Element prediction: Off
Number of isotope peaks used for i-FIT = 9

Monoisotopic Mass, Even Electron Ions
70 formula(e) evaluated with 1 results within limits (all results (up to 1000) for each mass)
Elements Used:
C: 0-70 H: 0-100 O: 0-50

13-Jun-2019 11:11:24 LCT Premier OUAZZANI_arcile166-2 19 (0.524) Cm (16:25)
2: TOF MS ES-

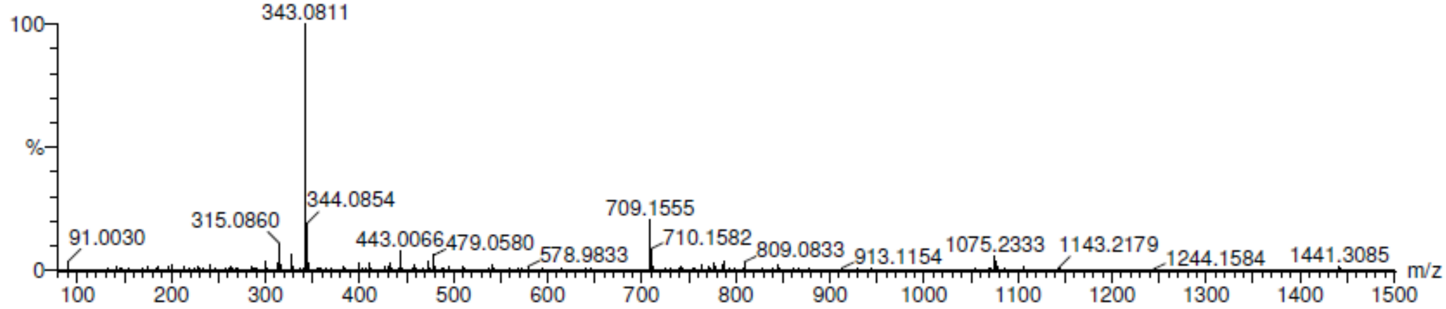

Minimum:				-1.5			
Maximum:		5.0	14.0	100.0			
Mass	Calc. Mass	mDa	PPM	DBE	i-FIT	i-FIT (Norm)	Formula
343.0811	343.0818	-0.7	-2.0	11.5	1111.6	0.0	C18 H15 O7

S25. HRESIMS [M-H]$^-$ of **5**

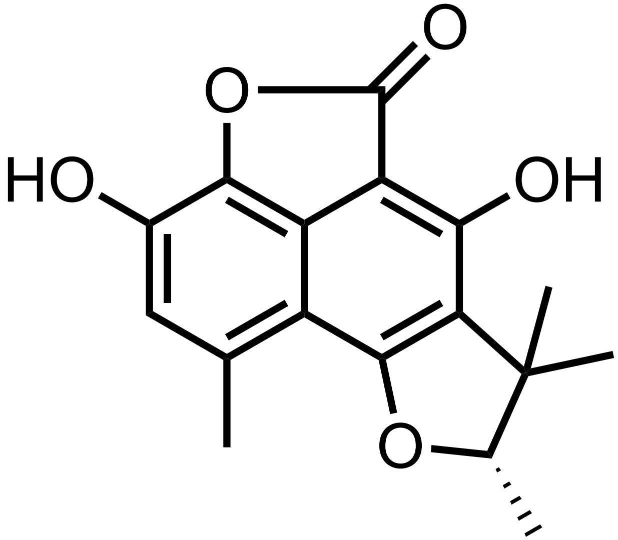

(-)-7,8-Dihydro-3,6-dihydroxy-1,7,7,8-tetramethyl-5H-furo- [2',3':5,6]naphtho[1,8-bc]furan-5-one (**6**)

$\alpha]_D$: -36.80° (c 0.10, MeOH)

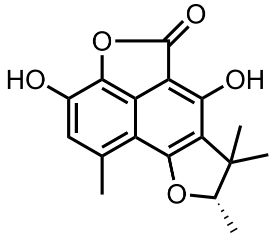

Compound **6**

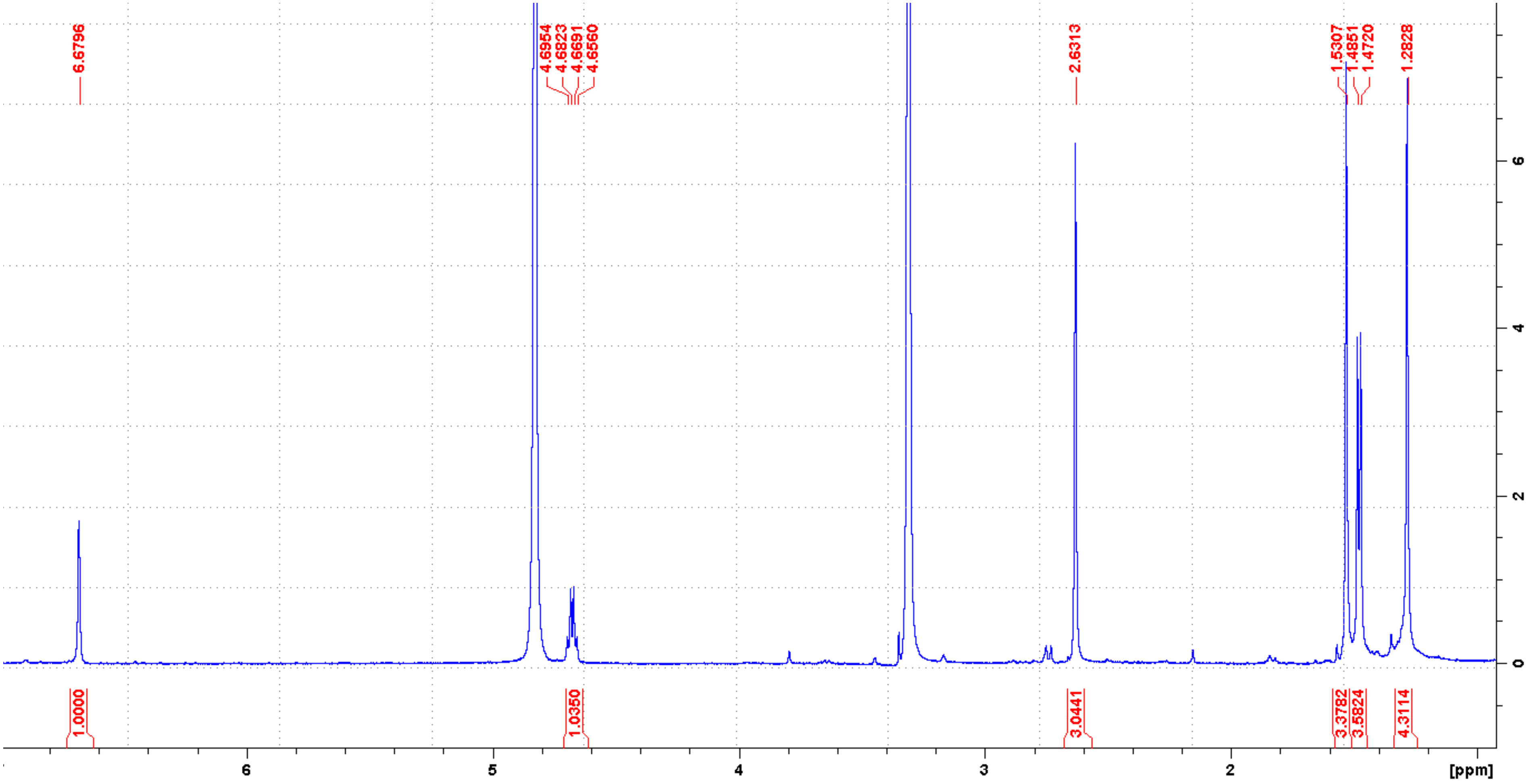

S26. ^{1}H NMR spectrum (500MHz, Acetone-d_6) of **6**

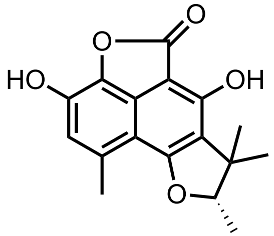

Compound **6**

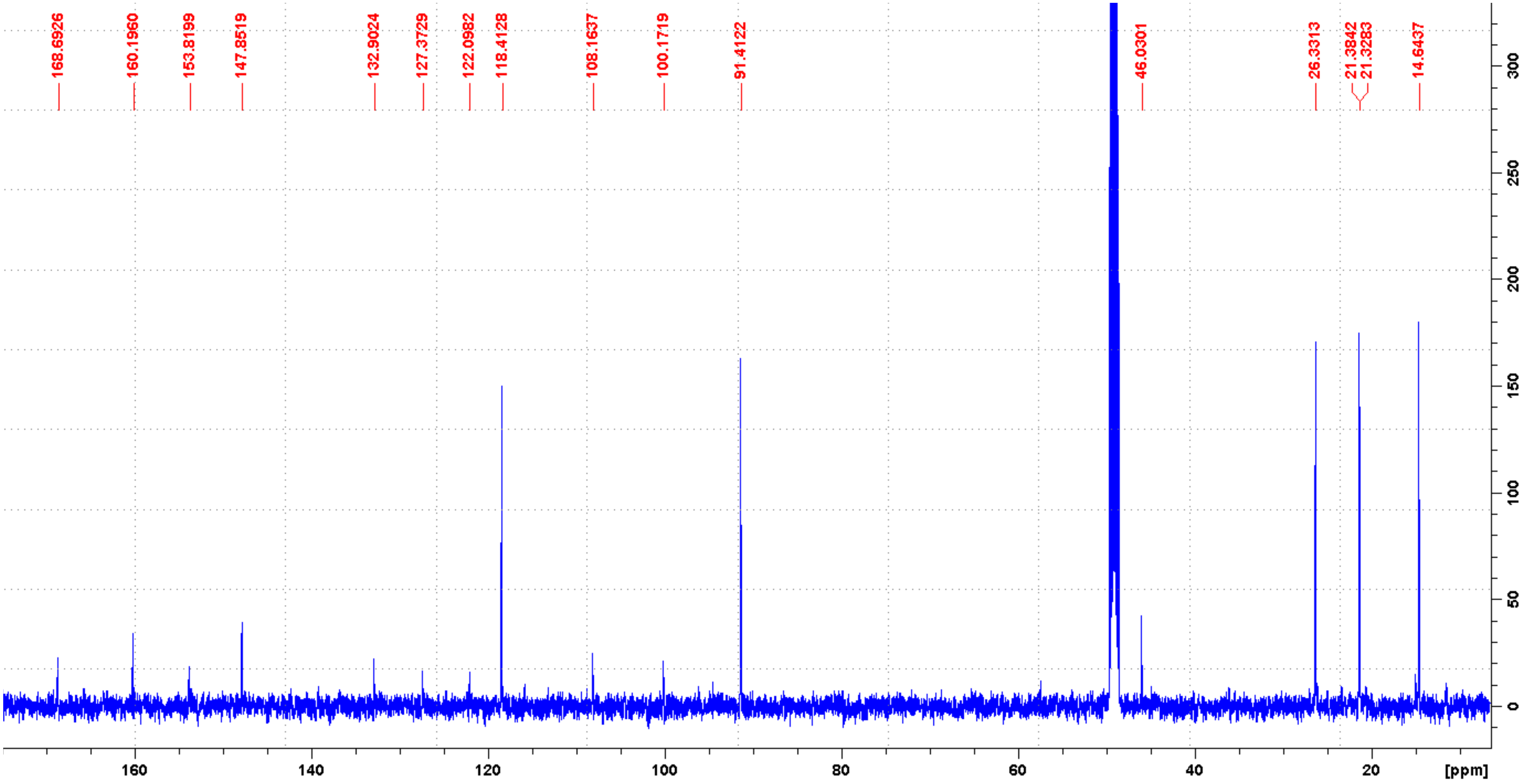

S27. ^{13}C NMR spectrum (125MHz, Acetone-d6) of **6**

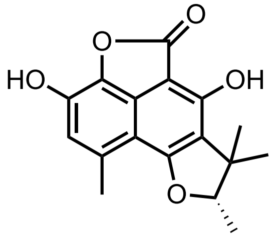

Compound **6**

Elemental Composition Report

Page 1

Single Mass Analysis

Tolerance = 10.0 PPM / DBE: min = -1.5, max = 100.0
Element prediction: Off
Number of isotope peaks used for i-FIT = 9

Monoisotopic Mass, Even Electron Ions
463 formula(e) evaluated with 4 results within limits (all results (up to 1000) for each mass)
Elements Used:
C: 0-50 H: 0-100 N: 0-10 O: 0-20

06-May-2019 16:17:58 LCT Premier OUAZZANI_glegoff108-3 22 (0.589) Cm (18:23-(33:68+3:12)x2.000)
1: TOF MS ES+

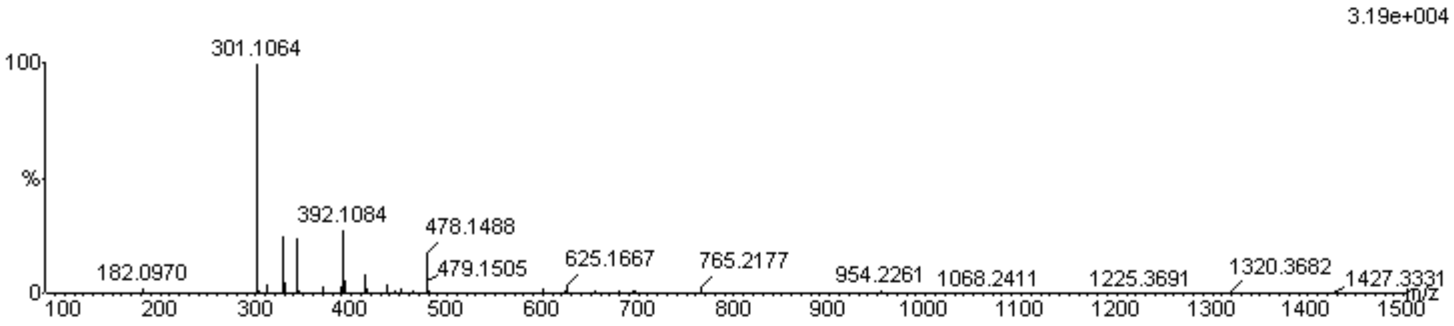

Minimum:				-1.5			
Maximum:		5.0	10.0	100.0			
Mass	Calc. Mass	mDa	PPM	DBE	i-FIT	i-FIT (Norm)	Formula
301.1064	301.1076	-1.2	-4.0	9.5	497.6	6.7	C17 H17 O5
	301.1049	1.5	5.0	10.5	490.9	0.0	C13 H13 N6 O3
	301.1089	-2.5	-8.3	14.5	496.5	5.6	C18 H13 N4 O
	301.1036	2.8	9.3	5.5	495.7	4.8	C12 H17 N2 O7

S28. HRESIMS $[M+H]^+$ of **6**

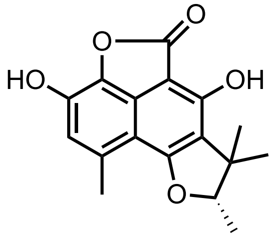

Compound **6**

Elemental Composition Report

Page 1

Single Mass Analysis

Tolerance = 10.0 PPM / DBE: min = -1.5, max = 100.0
Element prediction: Off
Number of isotope peaks used for i-FIT = 9

Monoisotopic Mass, Even Electron Ions
458 formula(e) evaluated with 5 results within limits (all results (up to 1000) for each mass)
Elements Used:
C: 0-50 H: 0-100 N: 0-10 O: 0-20

06-May-2019 16:17:58
LCT Premier OUAZZANI_glegoff108-3 19 (0.525) Cm (17:23-(33:60+3:15)x2.000)
2: TOF MS ES-

1.36e+004

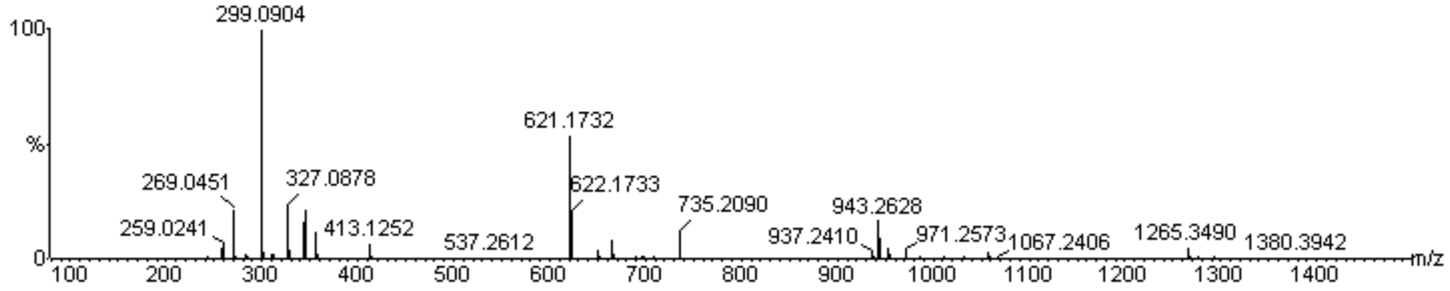

Mass	Calc. Mass	mDa	PPM	DBE	i-FIT	i-FIT (Norm)	Formula
Minimum:				-1.5			
Maximum:		5.0	10.0	100.0			
299.0904	299.0911	-0.7	-2.3	-1.5	192.9	23.4	C H15 N8 O10
	299.0893	1.1	3.7	11.5	179.4	9.9	C13 H11 N6 O3
	299.0919	-1.5	-5.0	10.5	169.6	0.0	C17 H15 O5
	299.0879	2.5	8.4	6.5	182.0	12.4	C12 H15 N2 O7
	299.0933	-2.9	-9.7	15.5	175.1	5.6	C18 H11 N4 O

S29. HRESIMS [M-H]$^-$ of **6**

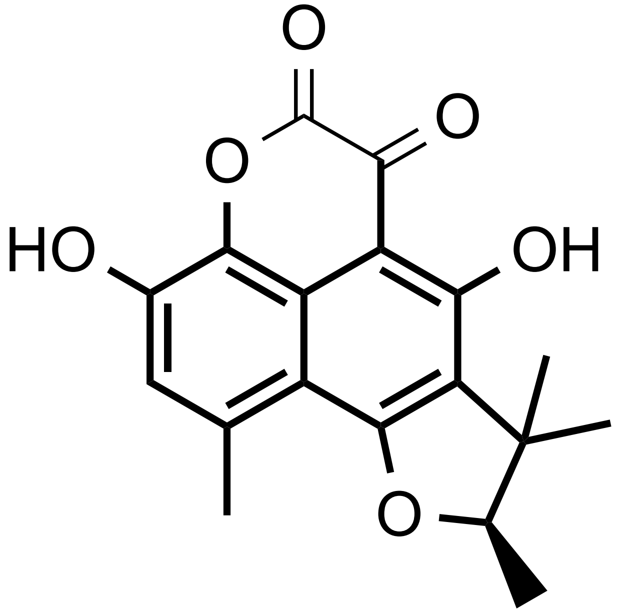

(+)-Sclerodorolide (**7**)

$[\alpha]_D$: +73.0° (c 0.10, MeOD)

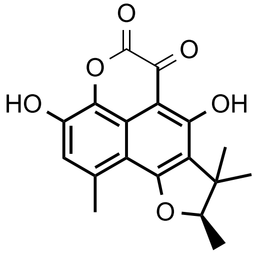

(+)-Sclerodorolide (**7**)

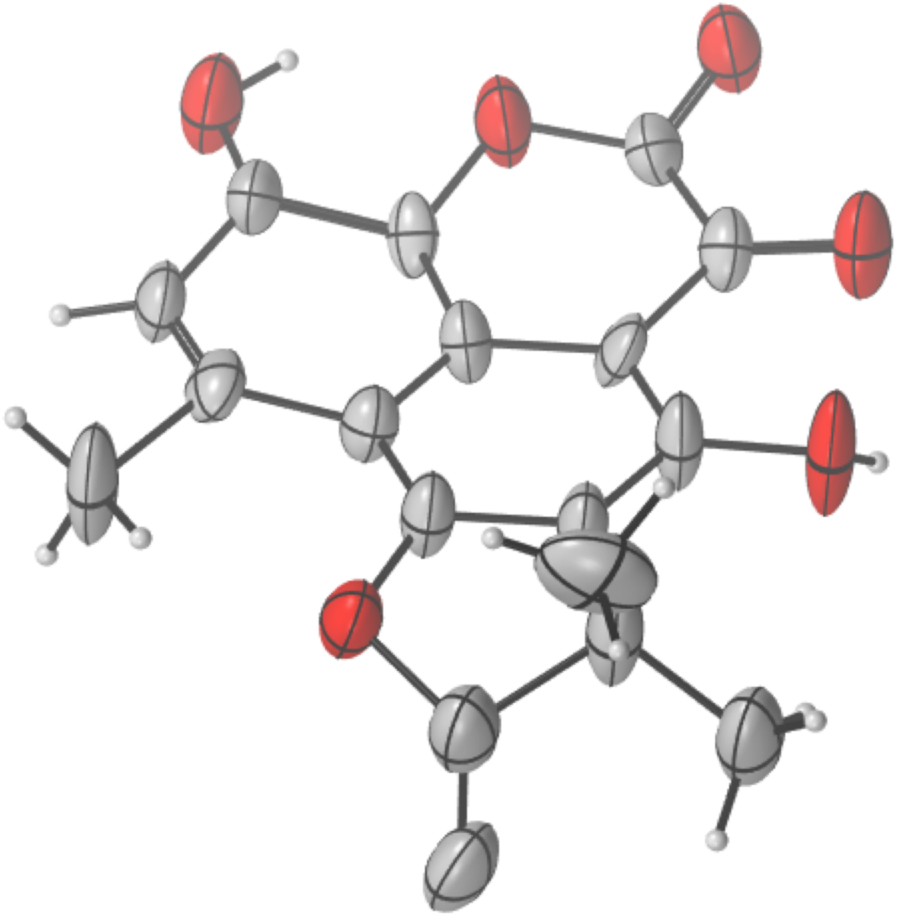

S30. X-ray crystallographic data of compound **7**

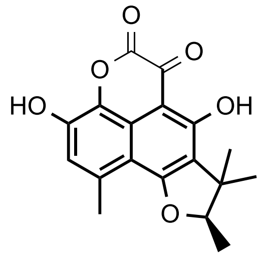

(+)-Scleroderolide (7)

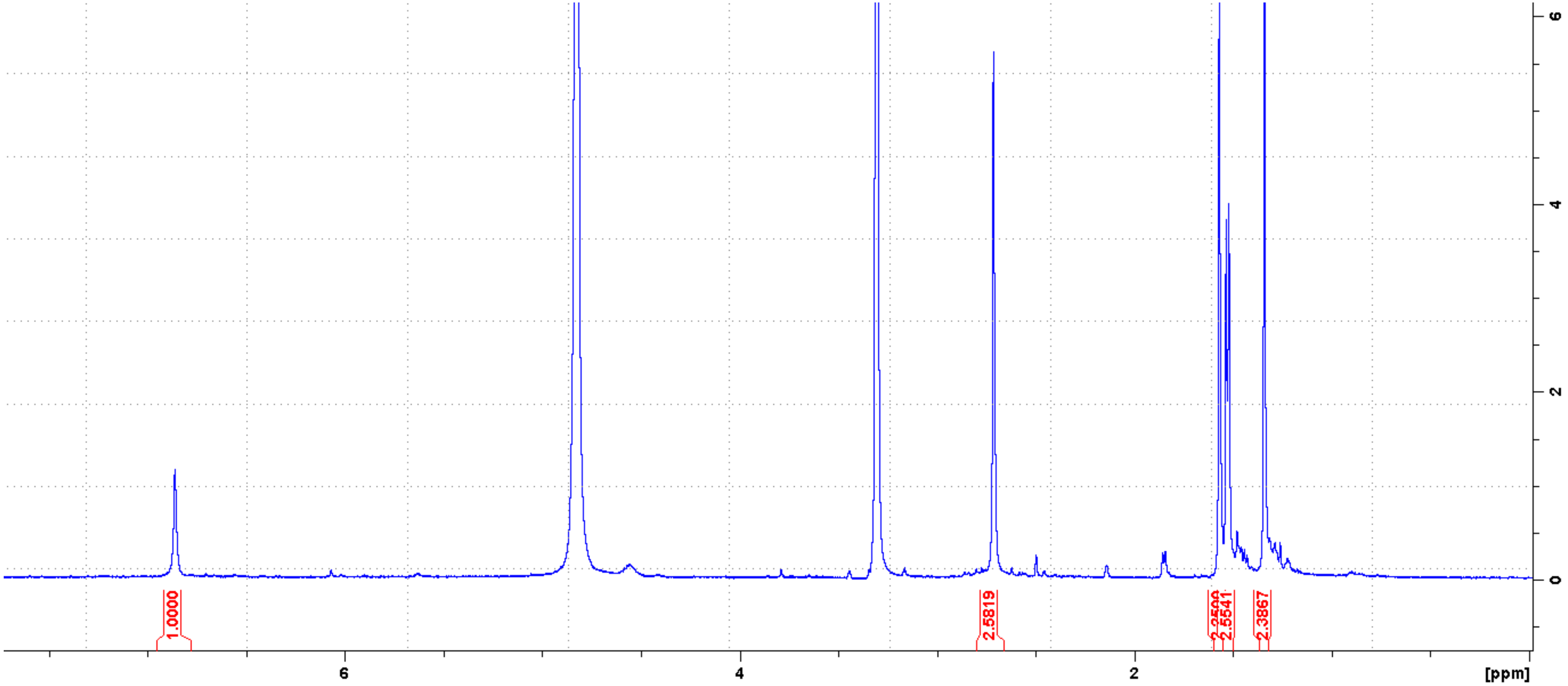

S31. ^{1}H NMR spectrum (500MHz, Acetone-d_6) of **7**

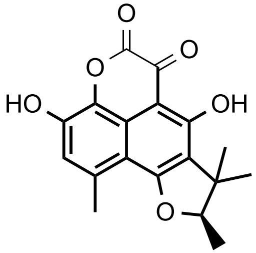

(+)-Scleroderolide (**7**)

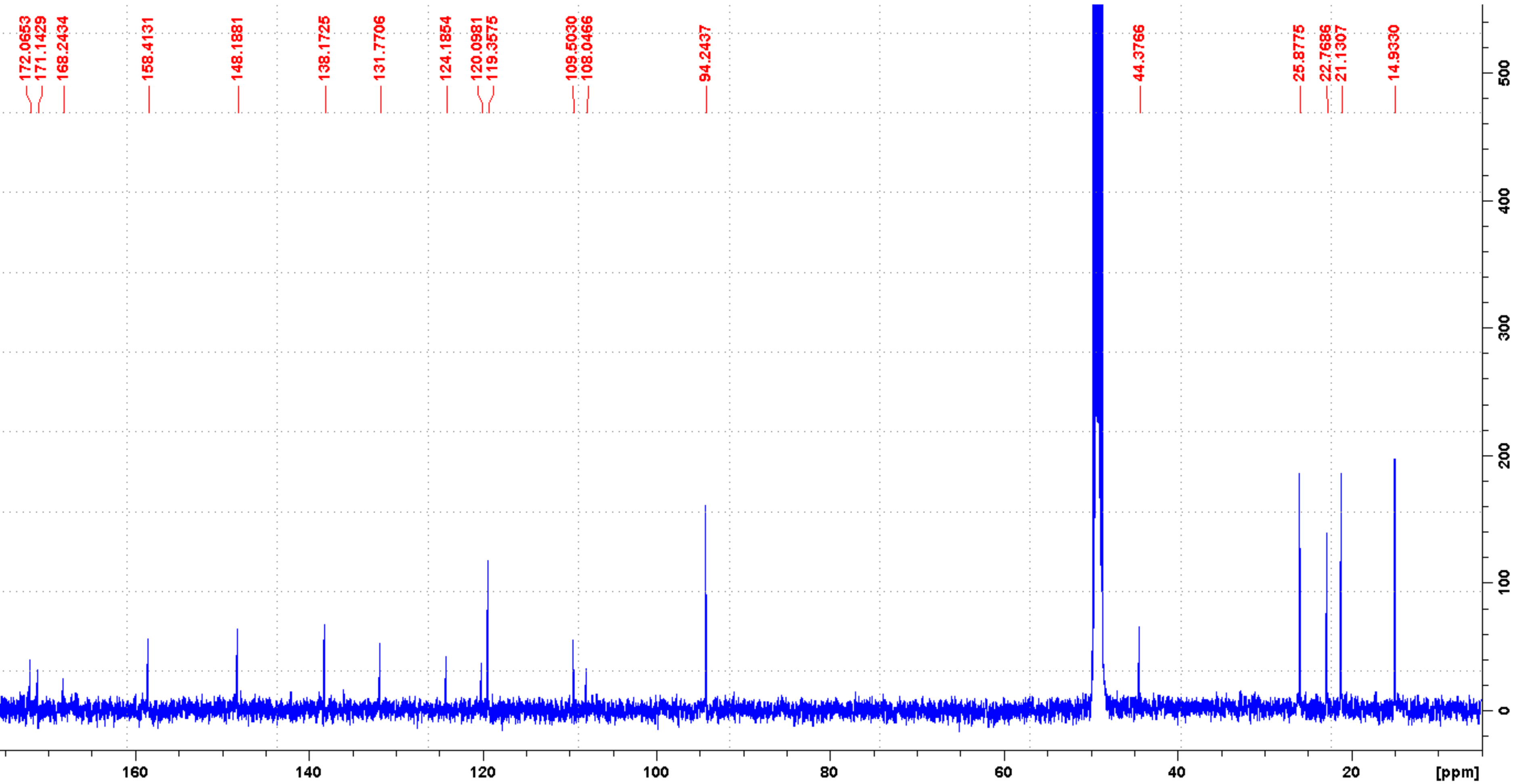

S32. ^{13}C NMR spectrum (125MHz, Acetone-d6) of **7**

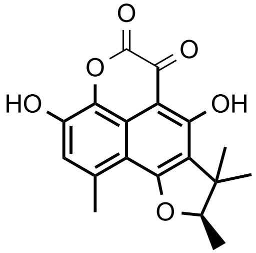

(+)-Scleroderolide (**7**)

Elemental Composition Report

Page 1

Single Mass Analysis

Tolerance = 10.0 PPM / DBE: min = -1.5, max = 100.0
Element prediction: Off
Number of isotope peaks used for i-FIT = 9

Monoisotopic Mass, Even Electron Ions
545 formula(e) evaluated with 5 results within limits (all results (up to 1000) for each mass)
Elements Used:
C: 0-50 H: 0-100 N: 0-10 O: 0-20

26-Apr-2019 15:28:26 LCT Premier OUAZZANI_arcile153-1 22 (0.590) Cm (18:24-(32:67+4:12)x2.000)
1: TOF MS ES+

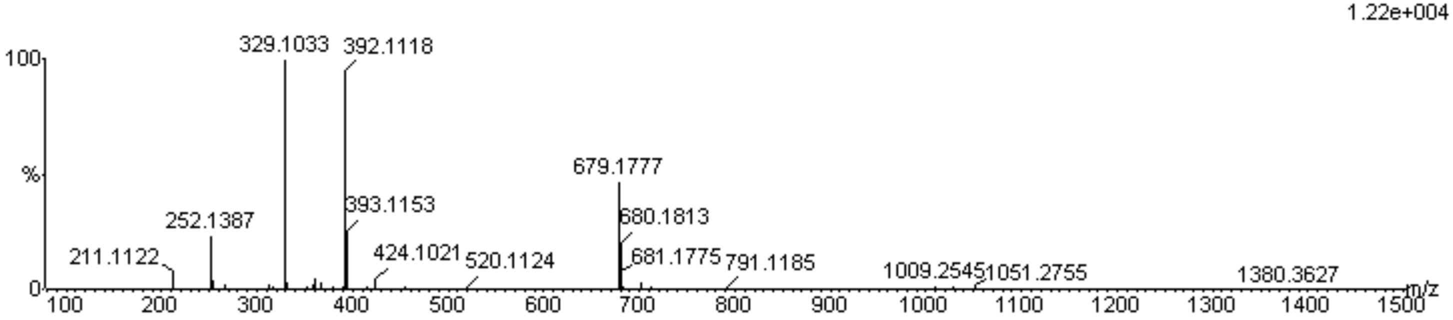

Minimum:				-1.5			
Maximum:		5.0	10.0	100.0			
Mass	Calc. Mass	mDa	PPM	DBE	i-FIT	i-FIT (Norm)	Formula
329.1033	329.1039	-0.6	-1.8	15.5	302.4	3.7	C19 H13 N4 O2
	329.1025	0.8	2.4	10.5	299.6	1.0	C18 H17 O6
	329.1017	1.6	4.9	-1.5	305.4	6.7	C2 H17 N8 O11
	329.1012	2.1	6.4	16.5	300.3	1.7	C15 H9 N10
	329.1057	-2.4	-7.3	2.5	299.4	0.8	C7 H17 N6 O9

S33. HRESIMS $[M+H]^+$ of **7**

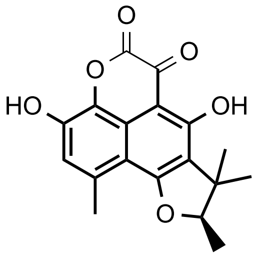

(+)-Scleroderolide (**7**)

Elemental Composition Report

Page 1

Single Mass Analysis

Tolerance = 10.0 PPM / DBE: min = -1.5, max = 100.0
Element prediction: Off
Number of isotope peaks used for i-FIT = 9

Monoisotopic Mass, Even Electron Ions
541 formula(e) evaluated with 7 results within limits (all results (up to 1000) for each mass)
Elements Used:
C: 0-50 H: 0-100 N: 0-10 O: 0-20

26-Apr-2019 15:28:26 LCT Premier OUAZZANI_arcile153-1 20 (0.543) Cm (18:22-(5:11+35:70)x2.000)
2: TOF MS ES-

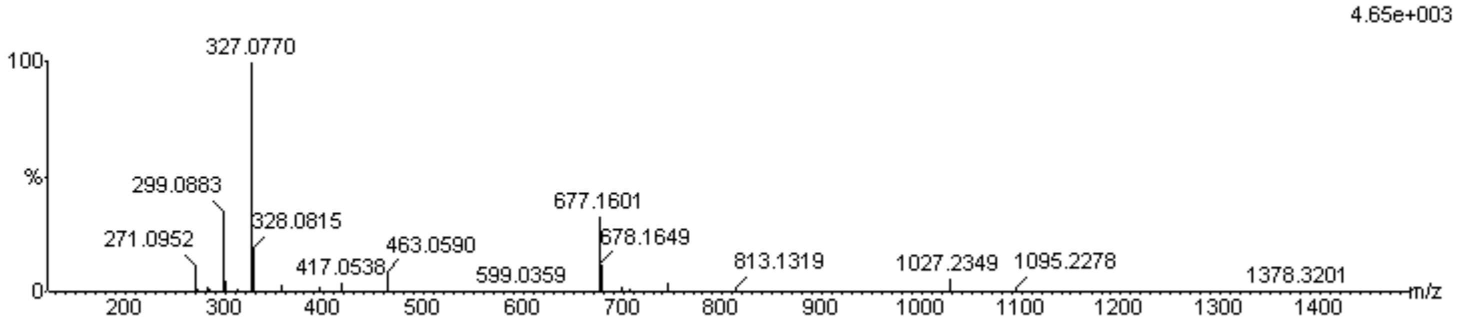

Minimum:				-1.5			
Maximum:		5.0	10.0	100.0			

Mass	Calc. Mass	mDa	PPM	DBE	i-FIT	i-FIT (Norm)	Formula
327.0770	327.0770	0.0	0.0	16.5	52.8	4.1	C20 H11 N2 O3
	327.0775	-0.5	-1.5	-1.5	53.9	5.2	C7 H19 O14
	327.0761	0.9	2.8	4.5	55.1	6.4	C4 H11 N10 O8
	327.0788	-1.8	-5.5	3.5	53.4	4.7	C8 H15 N4 O10
	327.0748	2.2	6.7	-0.5	57.4	8.7	C3 H15 N6 O12
	327.0743	2.7	8.3	17.5	48.8	0.1	C16 H7 N8 O
	327.0802	-3.2	-9.8	8.5	51.2	2.5	C9 H11 N8 O6

S34. HRESIMS $[M-H]^-$ of **7**

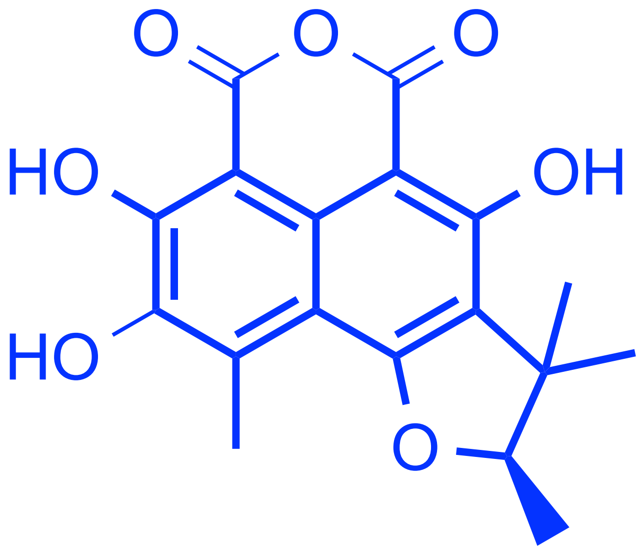

(+)-8-hydroxySclerodin (**8**)

$[\alpha]_D$: +66.01° (c 0.10, MeOH)

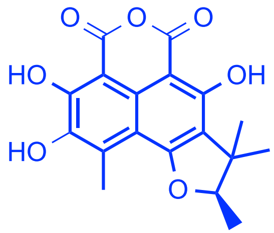

(+)-8-hydroxySclerodin (**8**)

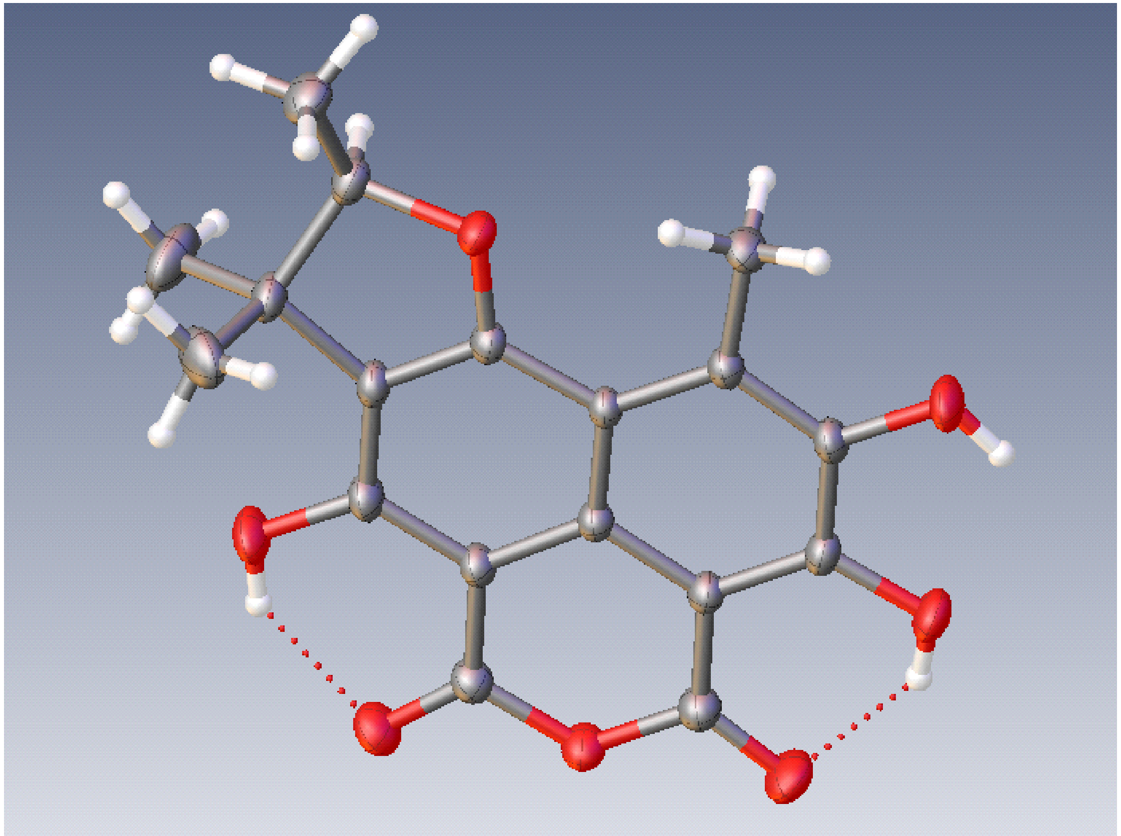

S35. X-ray crystallographic data of compound **8**

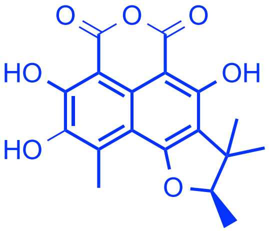

(+)-8-hydroxySclerodin (**8**)

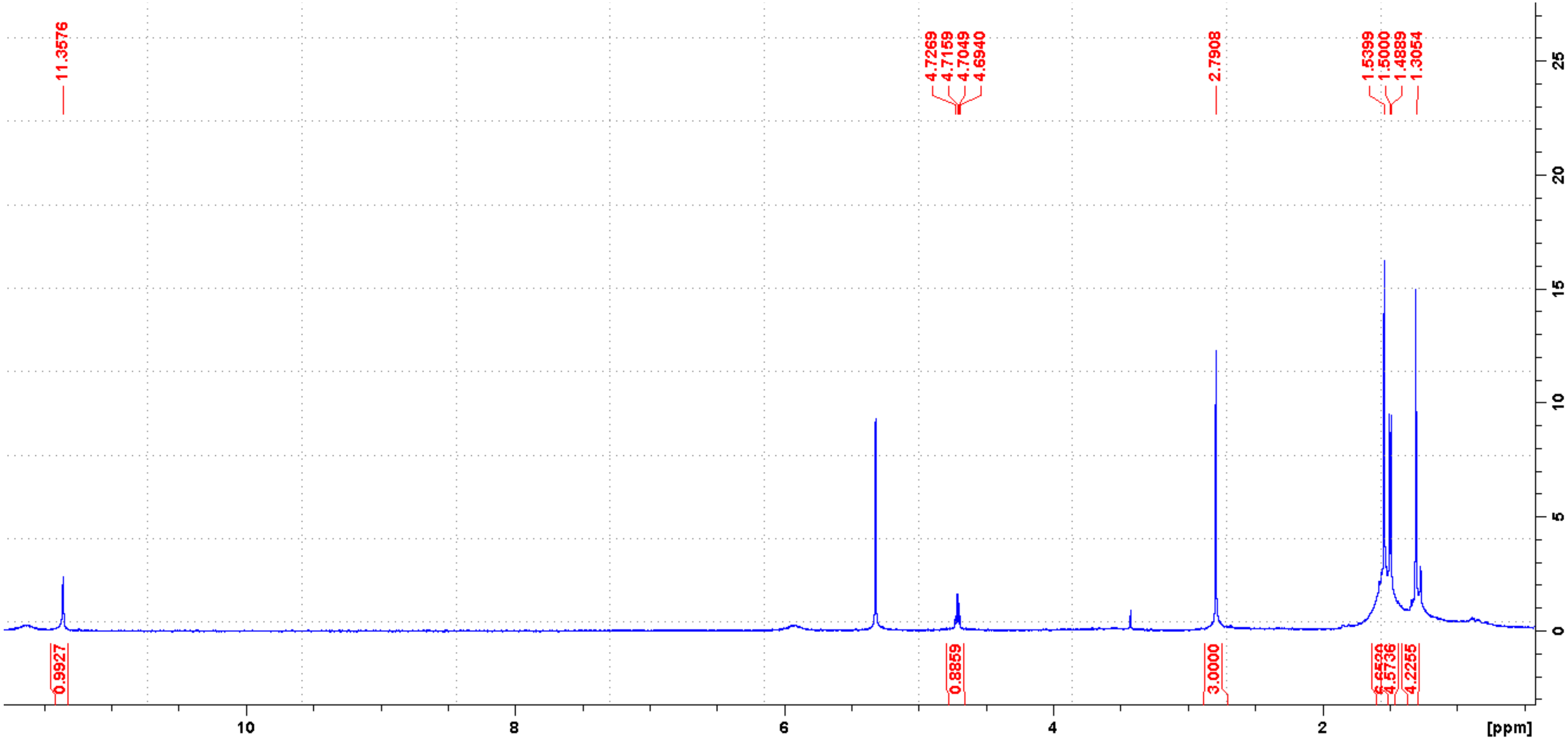

S36. ^{1}H NMR spectrum (500MHz, CD_2Cl_2) of **8**

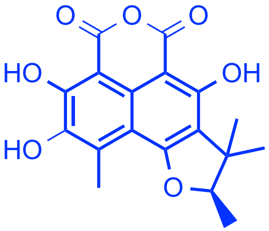

(+)-8-hydroxySclerodin (**8**)

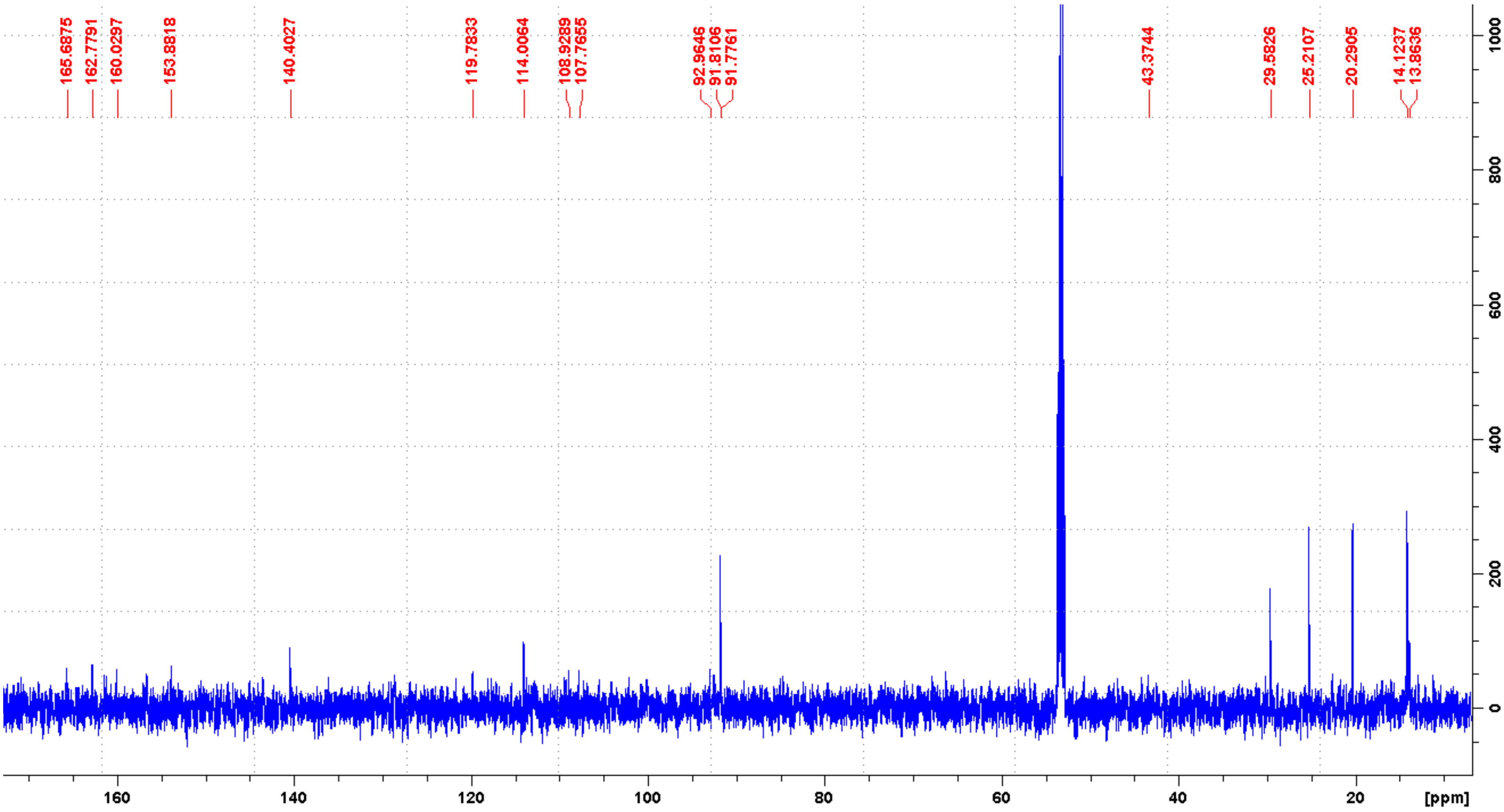

S37. ^{13}C NMR spectrum (125MHz, CD_2Cl_2) of **8**

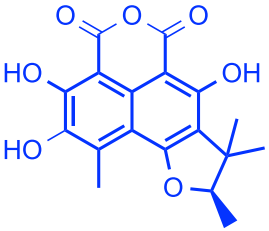

(+)-8-hydroxySclerodin (**8**)

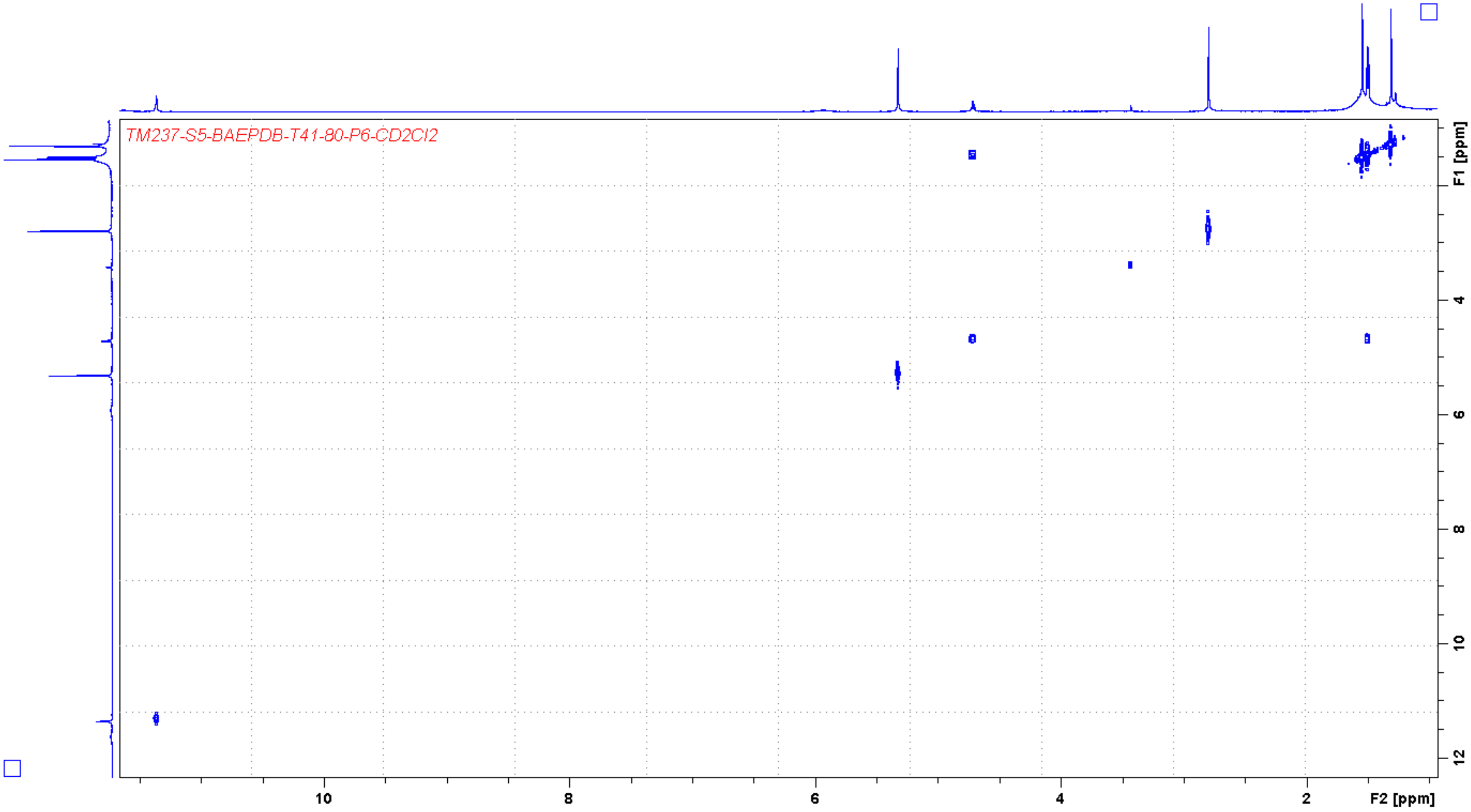

S38. ¹H-¹H COSY NMR spectrum (500MHz, CD_2Cl_2) of **8**

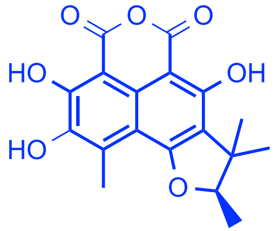

(+)-8-hydroxySclerodin (**8**)

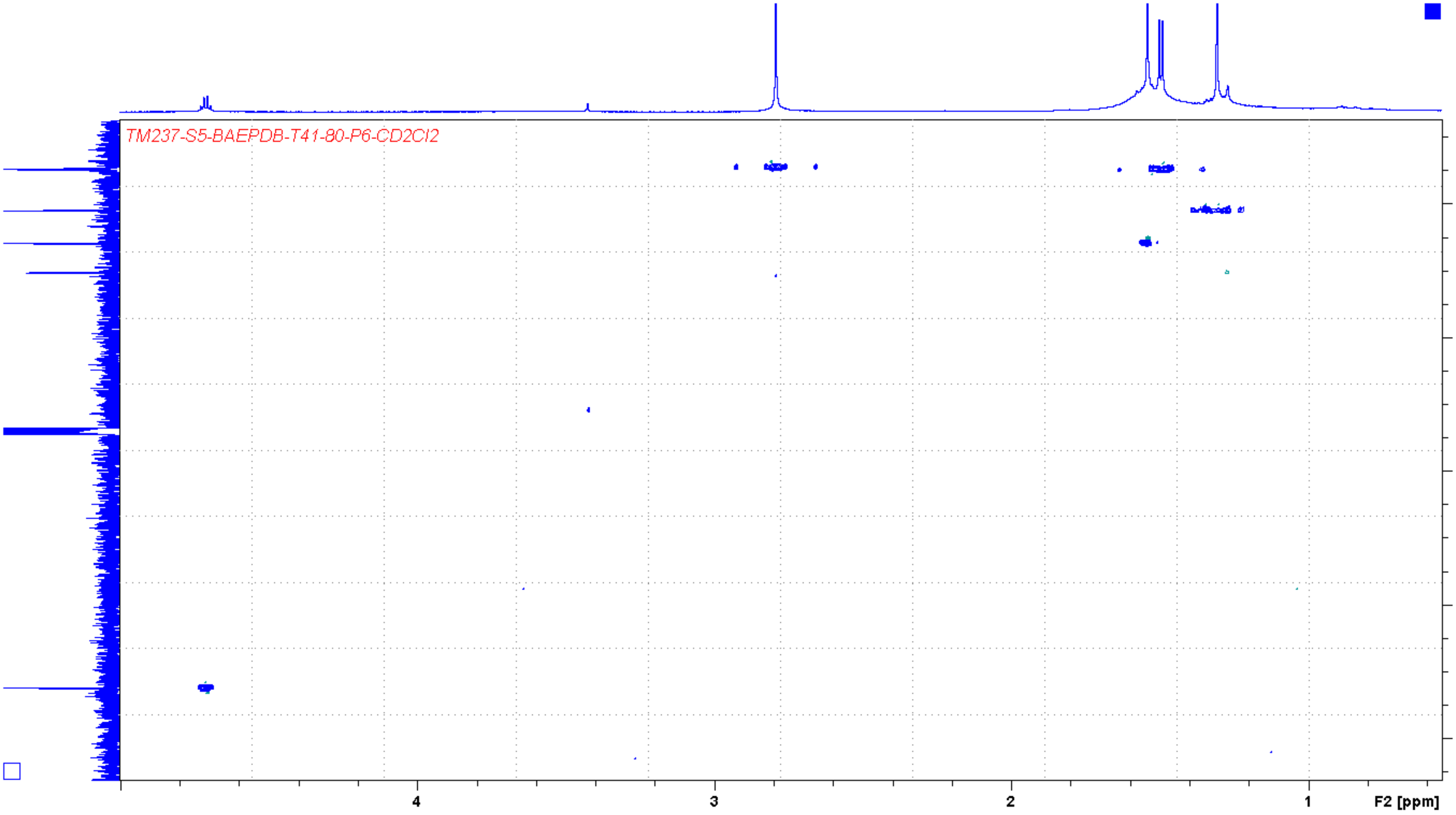

S39. ^{1}H-^{13}C HSQC spectrum (500 MHz, CD_2Cl_2) of **8**

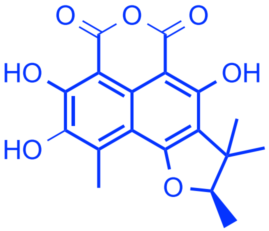

(+)-8-hydroxySclerodin (**8**)

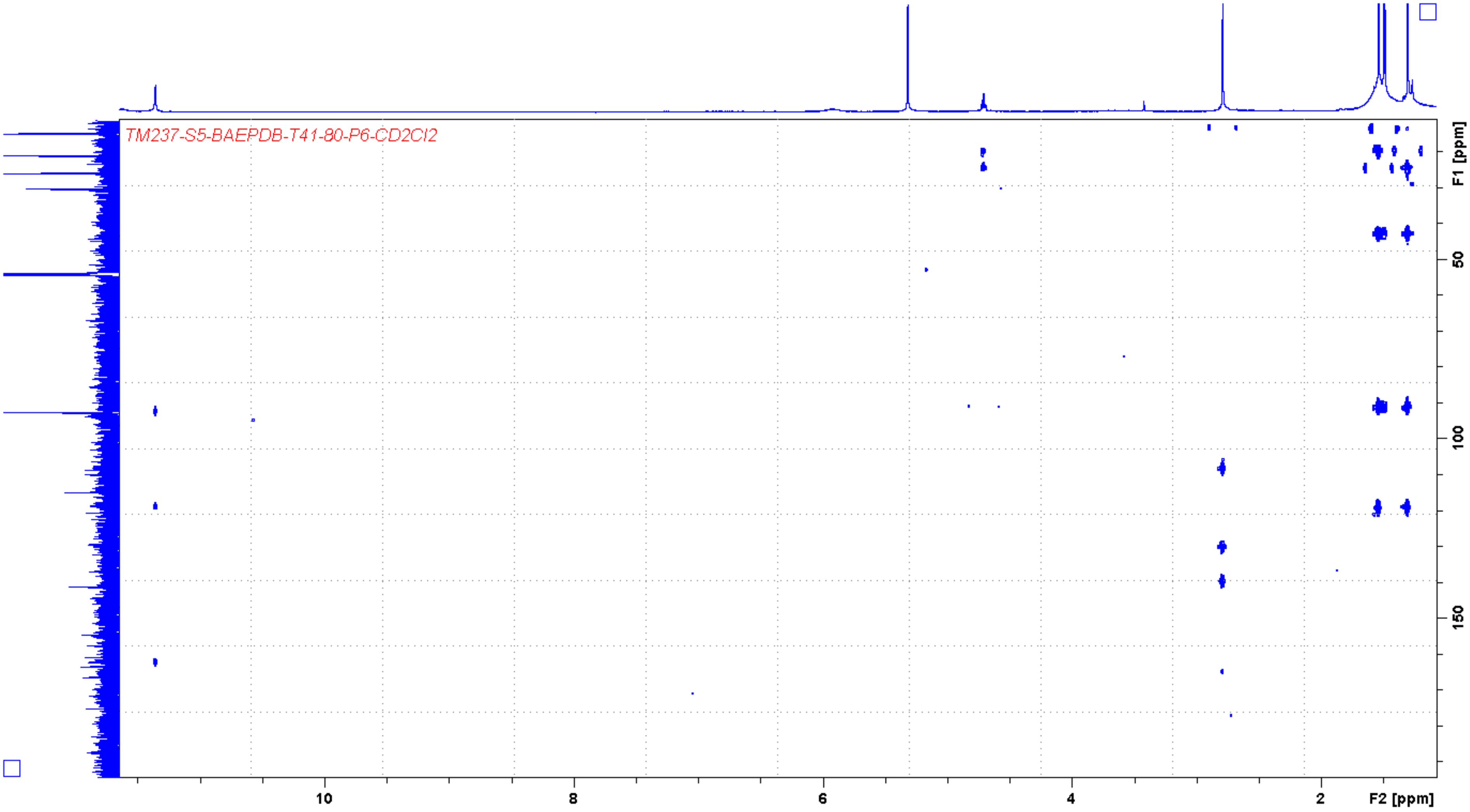

S40. ^{1}H-^{13}C HMBC spectrum (500 MHz, CD_2Cl_2) of **8**

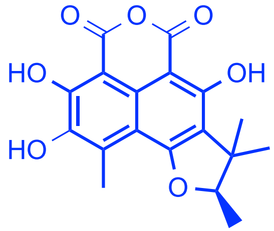

(+)-8-hydroxySclerodin (**8**)

Elemental Composition Report **Page 1**

Single Mass Analysis
Tolerance = 10.0 PPM / DBE: min = -1.5, max = 100.0
Element prediction: Off
Number of isotope peaks used for i-FIT = 9

Monoisotopic Mass, Even Electron Ions
594 formula(e) evaluated with 6 results within limits (all results (up to 1000) for each mass)
Elements Used:
C: 0-50 H: 0-100 N: 0-10 O: 0-20

26-Apr-2019 15:32:21 LCT Premier OUAZZANI_arcile153-2 21 (0.571) Cm (19:22)
1: TOF MS ES+

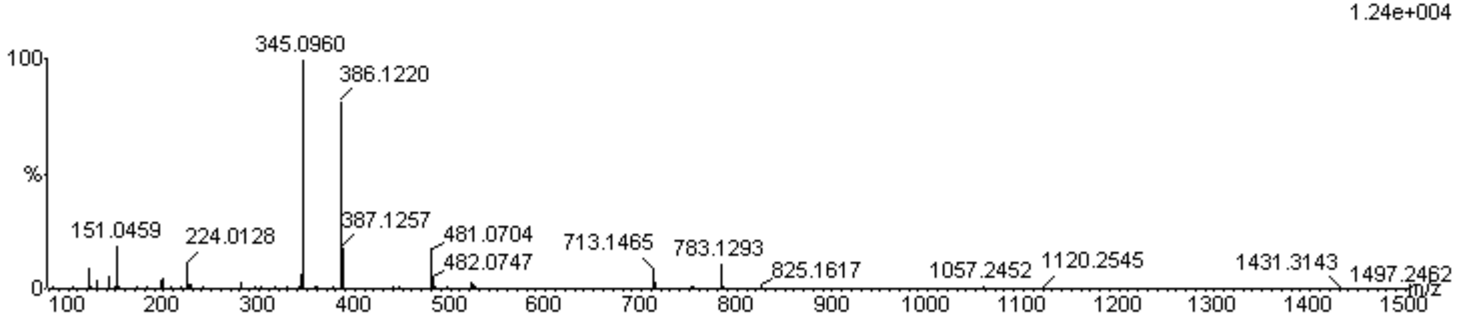

Minimum:				-1.5			
Maximum:		5.0	10.0	100.0			

Mass	Calc. Mass	mDa	PPM	DBE	i-FIT	i-FIT (Norm)	Formula
345.0960	345.0961	-0.1	-0.3	16.5	757.0	5.8	C15 H9 N10 O
	345.0966	-0.6	-1.7	-1.5	768.2	17.0	C2 H17 N8 O12
	345.0947	1.3	3.8	11.5	755.4	4.2	C14 H13 N6 O5
	345.0974	-1.4	-4.1	10.5	751.3	0.1	C18 H17 O7
	345.0934	2.6	7.5	6.5	755.7	4.5	C13 H17 N2 O9
	345.0988	-2.8	-8.1	15.5	754.9	3.7	C19 H13 N4 O3

S41. HRESIMS $[M+H]^+$ of **8**

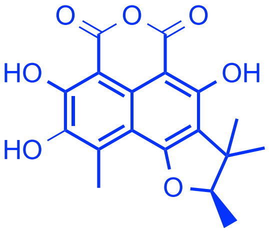

(+)-8-hydroxySclerodin (**8**)

Elemental Composition Report **Page 1**

Single Mass Analysis
Tolerance = 10.0 PPM / DBE: min = -1.5, max = 100.0
Element prediction: Off
Number of isotope peaks used for i-FIT = 9

Monoisotopic Mass, Even Electron Ions
591 formula(e) evaluated with 8 results within limits (all results (up to 1000) for each mass)
Elements Used:
C: 0-50 H: 0-100 N: 0-10 O: 0-20
26-Apr-2019 15:32:21 LCT Premier OUAZZANI_arcile153-2 22 (0.597) Cm (18:24)
2: TOF MS ES-

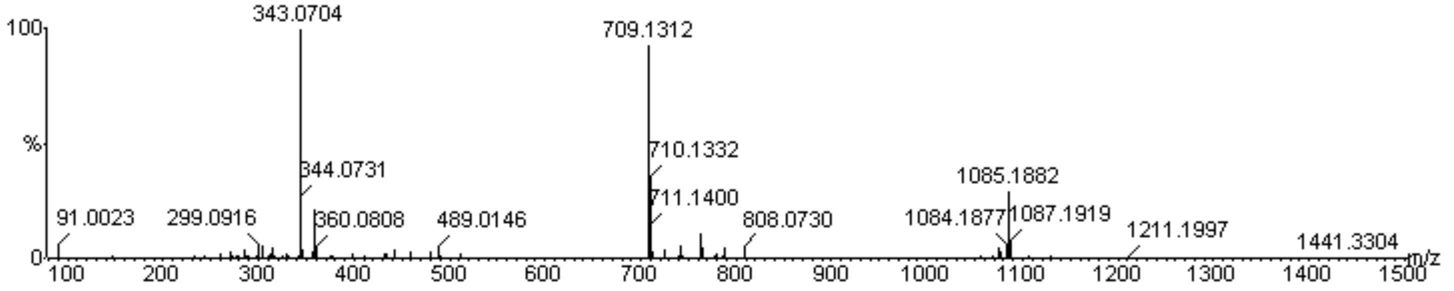

Minimum:				-1.5			
Maximum:		5.0	10.0	100.0			
Mass	Calc. Mass	mDa	PPM	DBE	i-FIT	i-FIT (Norm)	Formula
343.0704	343.0710	-0.6	-1.7	4.5	1022.5	12.2	C4 H11 N10 O9
	343.0697	0.7	2.0	-0.5	1023.2	12.9	C3 H15 N6 O13
	343.0692	1.2	3.5	17.5	1014.1	3.8	C16 H7 N8 O2
	343.0719	-1.5	-4.4	16.5	1011.1	0.8	C20 H11 N2 O4
	343.0724	-2.0	-5.8	-1.5	1021.3	11.0	C7 H19 O15
	343.0679	2.5	7.3	12.5	1015.2	4.8	C15 H11 N4 O6
	343.0732	-2.8	-8.2	21.5	1011.0	0.7	C21 H7 N6
	343.0737	-3.3	-9.6	3.5	1020.4	10.0	C8 H15 N4 O11

S42. HRESIMS [M-H]$^{-}$ of **8**

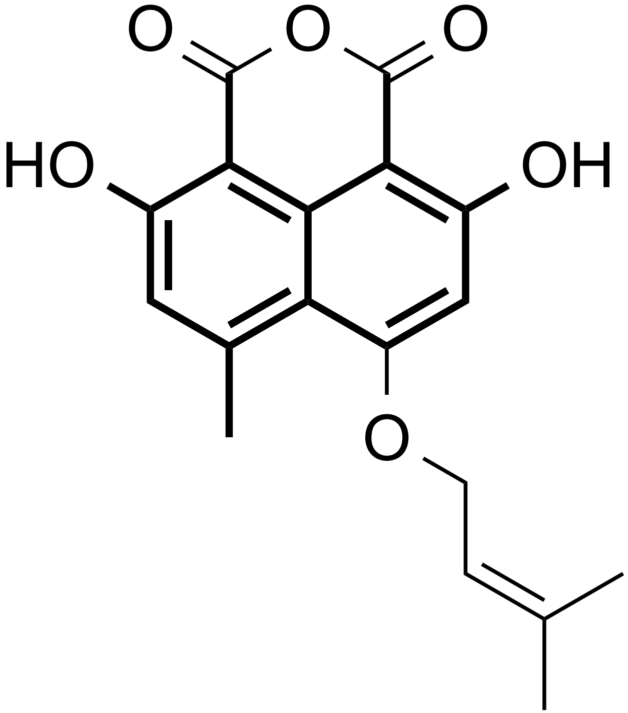

Coniosclerodin (**9**)

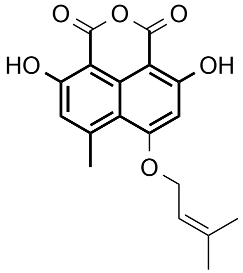

Conioscleroodin (**9**)

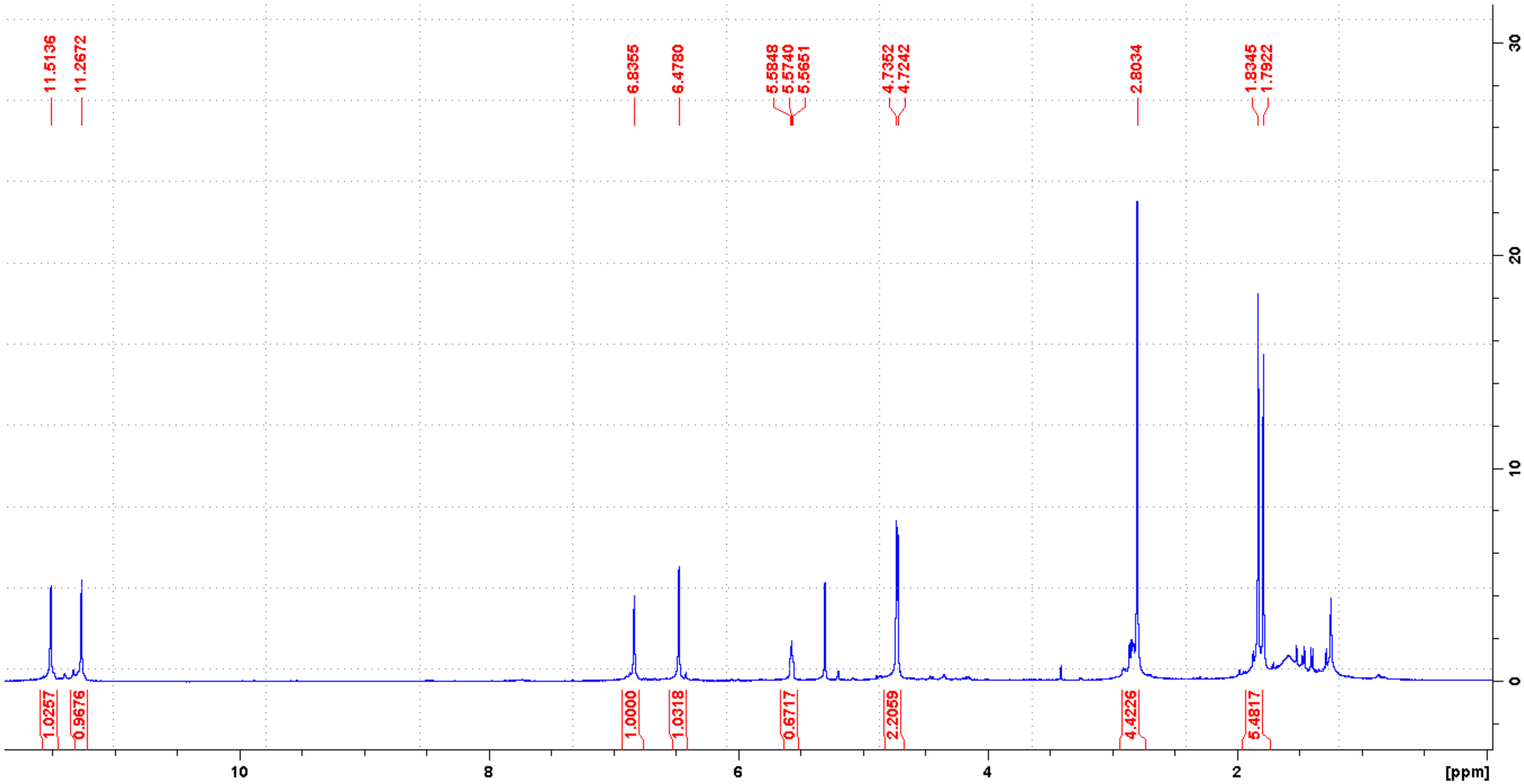

S43. ^{1}H NMR spectrum (500MHz, CD_2Cl_2) of **9**

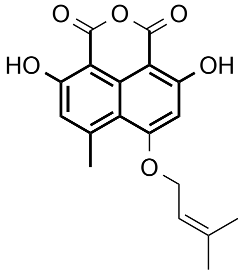

Conioscleródin (**9**)

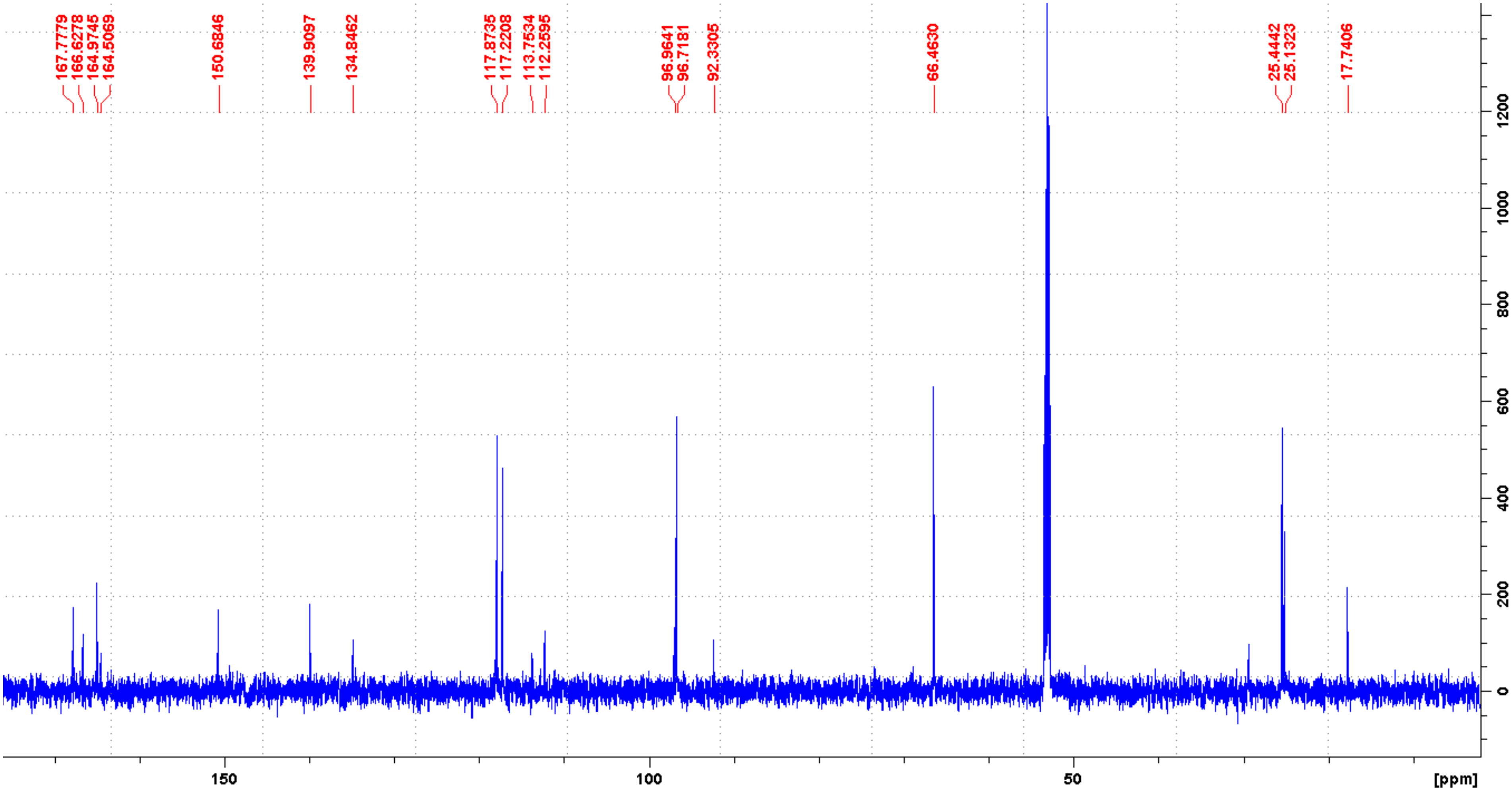

S44. ^{13}C NMR spectrum (125MHz, CD_2Cl_2) of **9**

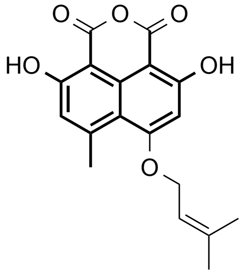

Conioscleródin (**9**)

Elemental Composition Report

Page 1

Single Mass Analysis

Tolerance = 10.0 PPM / DBE: min = -1.5, max = 100.0
Element prediction: Off
Number of isotope peaks used for i-FIT = 9

Monoisotopic Mass, Even Electron Ions
545 formula(e) evaluated with 5 results within limits (all results (up to 1000) for each mass)
Elements Used:
C: 0-50 H: 0-100 N: 0-10 O: 0-20

06-May-2019 16:27:02 LCT Premier OUAZZANI_glegoff109-1 21 (0.572) Cm (19:24-(33:66+3:12)x2.000)
1: TOF MS ES+

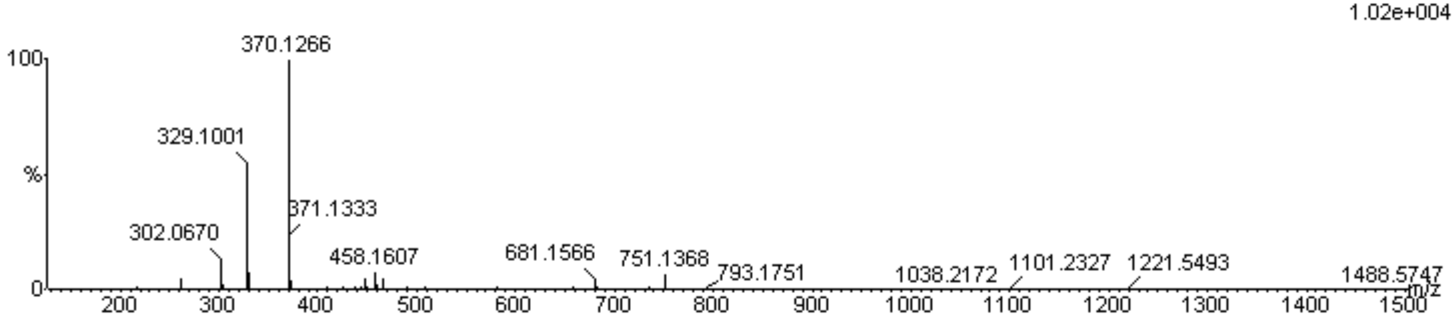

Minimum:				-1.5			
Maximum:		5.0	10.0	100.0			

Mass	Calc. Mass	mDa	PPM	DBE	i-FIT	i-FIT (Norm)	Formula
329.1001	329.0998	0.3	0.9	11.5	216.7	1.4	C14 H13 N6 O4
	329.1012	-1.1	-3.3	16.5	215.8	0.5	C15 H9 N10
	329.0985	1.6	4.9	6.5	218.0	2.6	C13 H17 N2 O8
	329.1017	-1.6	-4.9	-1.5	219.9	4.5	C2 H17 N8 O11
	329.1025	-2.4	-7.3	10.5	219.0	3.7	C18 H17 O6

S45. HRESIMS $[M+H]^+$ of **9**

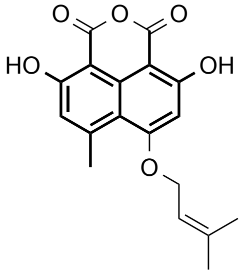

Conioscleroding (**9**)

Elemental Composition Report **Page 1**

Single Mass Analysis
Tolerance = 10.0 PPM / DBE: min = -1.5, max = 100.0
Element prediction: Off
Number of isotope peaks used for i-FIT = 9

Monoisotopic Mass, Even Electron Ions
541 formula(e) evaluated with 7 results within limits (all results (up to 1000) for each mass)
Elements Used:
C: 0-50 H: 0-100 N: 0-10 O: 0-20

06-May-2019 16:27:02 LCT Premier OUAZZANI_glegoff109-1 20 (0.543) Cm (18:22)
2: TOF MS ES-

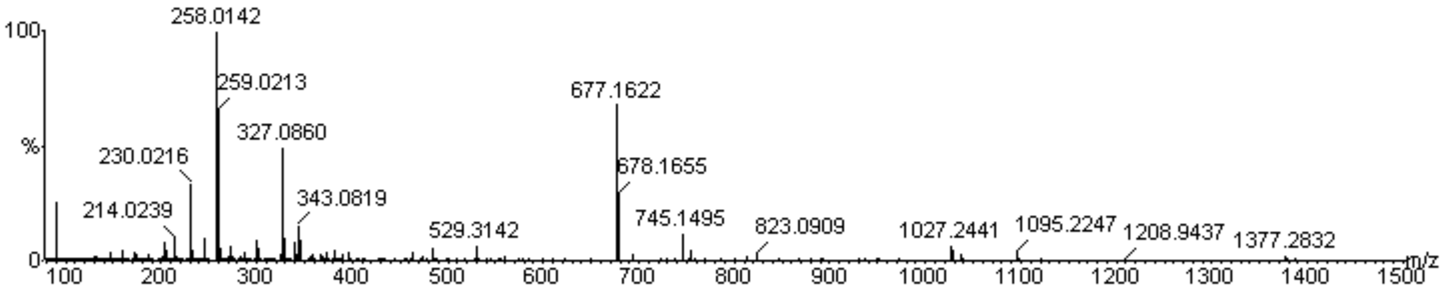

Minimum:				-1.5			
Maximum:		5.0	10.0	100.0			
Mass	Calc. Mass	mDa	PPM	DBE	i-FIT	i-FIT (Norm)	Formula
327.0860	327.0860	0.0	0.0	-0.5	812.4	8.2	C2 H15 N8 O11
	327.0855	0.5	1.5	17.5	807.4	3.3	C15 H7 N10
	327.0869	-0.9	-2.8	11.5	804.7	0.6	C18 H15 O6
	327.0842	1.8	5.5	12.5	807.0	2.8	C14 H11 N6 O4
	327.0882	-2.2	-6.7	16.5	805.4	1.2	C19 H11 N4 O2
	327.0887	-2.7	-8.3	-1.5	810.4	6.2	C6 H19 N2 O13
	327.0828	3.2	9.8	7.5	807.4	3.2	C13 H15 N2 O8

S46. HRESIMS [M-H]$^-$ of **9**

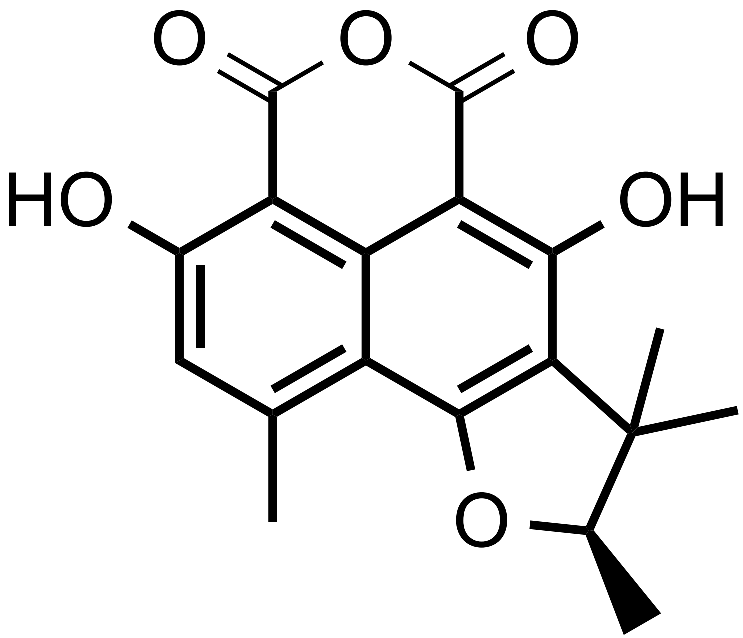

(+)-Sclerodin (**10**)

$[\alpha]_D$: +20.01° (c 0.10, CH_2Cl_2)

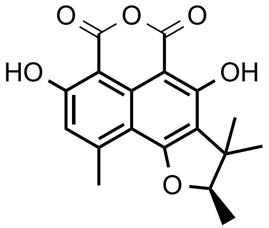

(+)-Sclerodin (**10**)

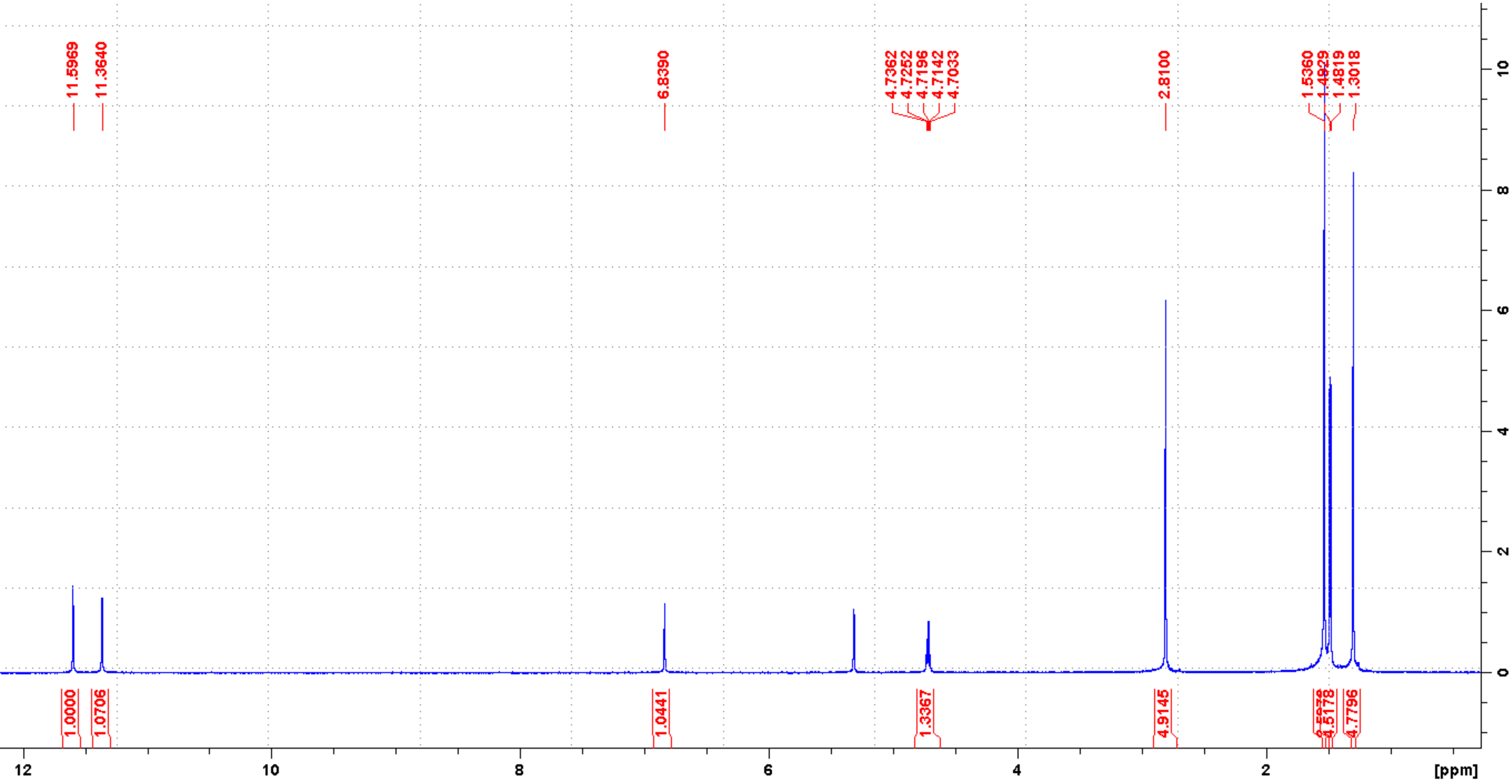

S47. ^{1}H NMR spectrum (500MHz, CD_2Cl_2) of **10**

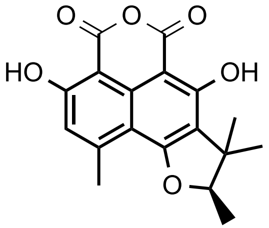

(+)-Sclerodin (**10**)

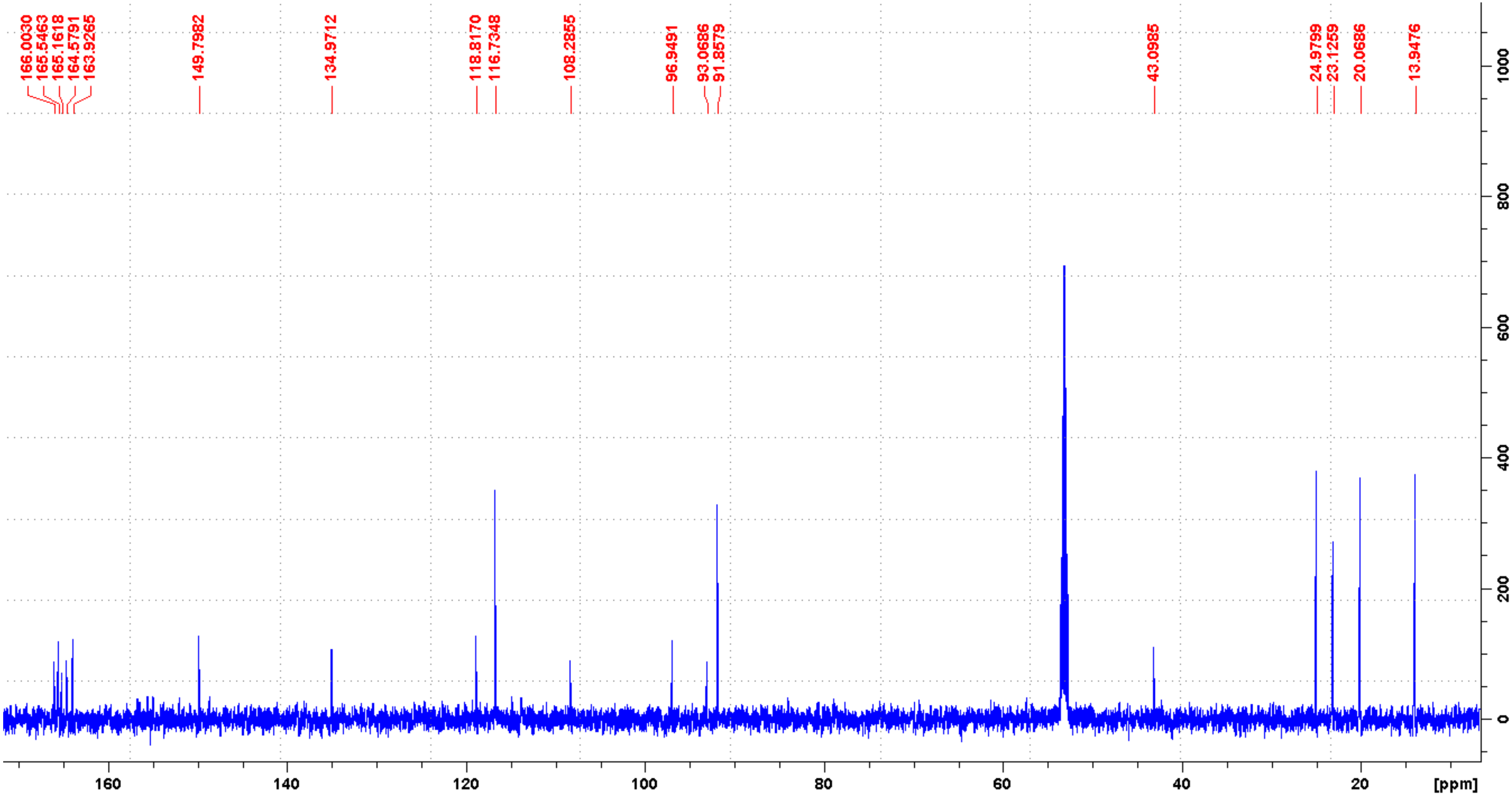

S48. ^{13}C NMR spectrum (125MHz, CD_2Cl_2) of **10**

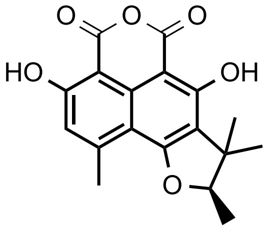

Elemental Composition Report

Page 1

Single Mass Analysis

Tolerance = 10.0 PPM / DBE: min = -1.5, max = 100.0
Element prediction: Off
Number of isotope peaks used for i-FIT = 9

Monoisotopic Mass, Even Electron Ions
545 formula(e) evaluated with 6 results within limits (all results (up to 1000) for each mass)
Elements Used:
C: 0-50 H: 0-100 N: 0-10 O: 0-20

26-Apr-2019 15:35:19 LCT Premier OUAZZANI_arcile153-3 22 (0.590) Cm (20:23-(33:67+3:13)x2.000)
1: TOF MS ES+

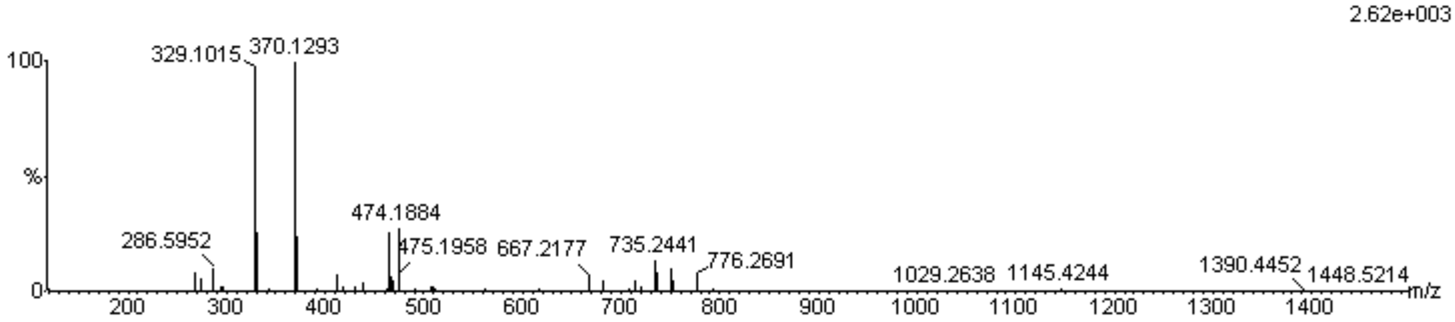

Minimum:				-1.5			
Maximum:		5.0	10.0	100.0			
Mass	Calc. Mass	mDa	PPM	DBE	i-FIT	i-FIT (Norm)	Formula
329.1015	329.1017	-0.2	-0.6	-1.5	65.0	13.4	C2 H17 N8 O11
	329.1012	0.3	0.9	16.5	52.2	0.6	C15 H9 N10
	329.1025	-1.0	-3.0	10.5	55.4	3.8	C18 H17 O6
	329.0998	1.7	5.2	11.5	55.9	4.3	C14 H13 N6 O4
	329.1039	-2.4	-7.3	15.5	52.6	0.9	C19 H13 N4 O2
	329.0985	3.0	9.1	6.5	58.8	7.2	C13 H17 N2 O8

S49. HRESIMS $[M+H]^+$ of **10**

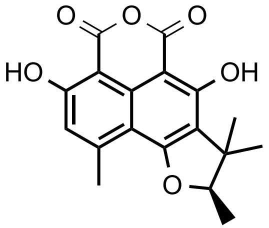

(+) Sclerodin (**10**)

Elemental Composition Report — **Page 1**

Single Mass Analysis

Tolerance = 10.0 PPM / DBE: min = -1.5, max = 100.0
Element prediction: Off
Number of isotope peaks used for i-FIT = 9

Monoisotopic Mass, Even Electron Ions
541 formula(e) evaluated with 6 results within limits (all results (up to 1000) for each mass)
Elements Used:
C: 0-50 H: 0-100 N: 0-10 O: 0-20

26-Apr-2019 15:35:19 LCT Premier OUAZZANI_arcile153-3 21 (0.581) Cm (20:22-30:63x2.000)
2: TOF MS ES-

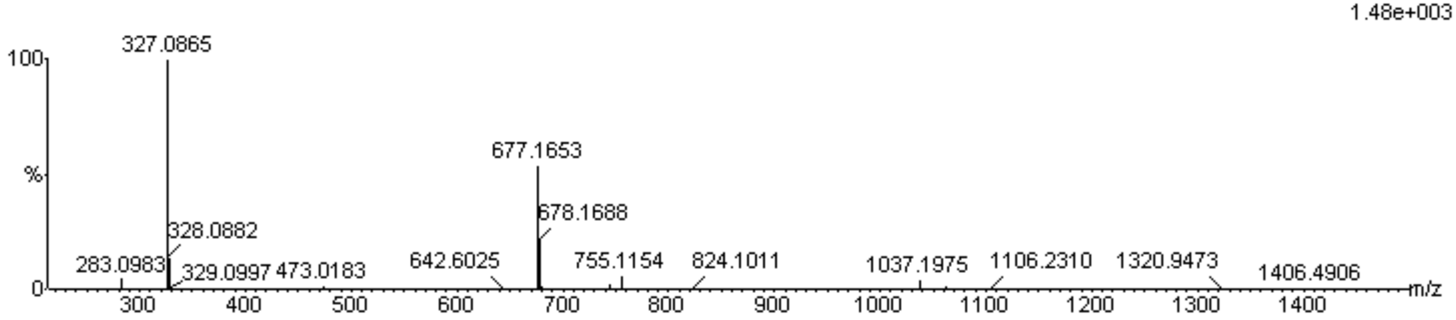

Minimum:				-1.5			
Maximum:		5.0	10.0	100.0			
Mass	Calc. Mass	mDa	PPM	DBE	i-FIT	i-FIT (Norm)	Formula
327.0865	327.0869	-0.4	-1.2	11.5	28.9	4.5	C18 H15 O6
	327.0860	0.5	1.5	-0.5	28.7	4.3	C2 H15 N8 O11
	327.0855	1.0	3.1	17.5	24.7	0.3	C15 H7 N10
	327.0882	-1.7	-5.2	16.5	29.1	4.7	C19 H11 N4 O2
	327.0887	-2.2	-6.7	-1.5	29.8	5.4	C6 H19 N2 O13
	327.0842	2.3	7.0	12.5	25.8	1.5	C14 H11 N6 O4

S50. HRESIMS $[M-H]^-$ of **10**

Identification code	**(+)-hydroxysclerodin**	
Empirical formula	C18 H16 O7	
Formula weight	344.31	
Temperature	293(2) K	
Wavelength	0.71073 Å	
Crystal system	Orthorhombic	
Space group	$P2_12_12_1$	
Unit cell dimensions	a = 6.6249(2) Å	α= 90°.
	b = 10.0615(3) Å	β= 90°.
	c = 22.5473(6) Å	γ = 90°.
Volume	1502.92(8) $Å^3$	
Z	4	
Density (calculated)	1.522 Mg/m^3	
Absorption coefficient	0.118 mm^{-1}	
F(000)	720	
Crystal size	0.15 x 0.15 x 0.1 mm^3	
Theta range for data collection	3.567 to 30.034°.	
Index ranges	$-9 \le h \le 9$, $-14 \le k \le 14$, $-31 \le l \le 31$	
Reflections collected	47173	
Independent reflections	4374 [R(int) = 0.0456]	
Completeness to theta = 25.242°	99.5 %	
Absorption correction	Semi-empirical from equivalents	
Max. and min. transmission	1.00000 and 0.49389	
Refinement method	Full-matrix least-squares on F^2	
Data / restraints / parameters	4370 / 0 / 233	
Goodness-of-fit on F^2	1.072	
Final R indices [$I>2\sigma(I)$]	R1 = 0.0389, wR2 = 0.1064	
R indices (all data)	R1 = 0.0428, wR2 = 0.1093	
Absolute structure Flack parameter	-0.2(2)	
Largest diff. peak and hole	0.414 and -0.245 e.$Å^{-3}$	

S51. Crystal data and structure refinement for Compound **8**

	x	y	z	U(eq)
O(1)	7701(2)	564(1)	8284(1)	35(1)
C(1)	7592(2)	1251(1)	7774(1)	24(1)
O(2)	7586(2)	-859(1)	7289(1)	36(1)
C(2)	7533(2)	498(1)	7248(1)	24(1)
O(3)	7739(3)	2918(1)	8783(1)	41(1)
C(3)	7425(2)	1081(1)	6696(1)	22(1)
O(4)	7593(2)	4744(1)	8250(1)	31(1)
C(4)	7366(2)	2502(1)	6661(1)	20(1)
O(5)	7493(2)	6641(1)	7766(1)	35(1)
C(5)	7436(2)	3277(1)	7187(1)	21(1)
O(6)	7305(2)	6701(1)	6591(1)	37(1)
C(6)	7548(2)	2631(1)	7743(1)	22(1)
O(7)	7134(2)	2718(1)	5580(1)	26(1)
C(7)	7630(3)	3384(1)	8286(1)	27(1)
C(8)	7485(2)	5438(1)	7720(1)	25(1)
C(9)	7397(2)	4690(1)	7176(1)	23(1)
C(10)	7291(3)	5372(1)	6635(1)	25(1)
C(11)	7166(3)	4636(1)	6112(1)	24(1)
C(12)	7216(2)	3264(1)	6128(1)	22(1)
C(13)	6494(2)	3789(1)	5173(1)	27(1)
C(14)	7180(3)	5095(1)	5472(1)	28(1)
C(15)	7260(3)	3482(2)	4560(1)	36(1)
C(16)	5692(4)	6203(2)	5321(1)	50(1)
C(17)	9335(3)	5505(2)	5299(1)	44(1)
C(18)	7363(3)	181(1)	6165(1)	30(1)

S52. Atomic coordinates (x 104) and equivalent isotropic displacement parameters (Å2x 103) for Compound **8** . U(eq) is defined as one third of the trace of the orthogonalized Uij tensor.

O(1)-C(1)	1.3451(15)
C(1)-C(6)	1.3899(18)
C(1)-C(2)	1.4070(18)
O(2)-C(2)	1.3686(15)
C(2)-C(3)	1.3795(17)
O(3)-C(7)	1.2161(18)
C(3)-C(4)	1.4316(16)
C(3)-C(18)	1.5017(17)
O(4)-C(7)	1.3708(17)
O(4)-C(8)	1.3866(16)
C(4)-C(5)	1.4221(16)
C(4)-C(12)	1.4274(16)
O(5)-C(8)	1.2152(17)
C(5)-C(6)	1.4134(16)
C(5)-C(9)	1.4216(17)
O(6)-C(10)	1.3410(15)
C(6)-C(7)	1.4414(18)
O(7)-C(12)	1.3533(15)
O(7)-C(13)	1.4775(16)
C(8)-C(9)	1.4397(17)
C(9)-C(10)	1.4029(17)
C(10)-C(11)	1.3948(18)
C(11)-C(12)	1.3817(17)
C(11)-C(14)	1.5146(18)
C(13)-C(15)	1.506(2)
C(13)-C(14)	1.545(2)
C(14)-C(16)	1.527(2)
C(14)-C(17)	1.536(3)

O(1)-C(1)-C(6)	123.89(12)
O(1)-C(1)-C(2)	116.42(12)
C(6)-C(1)-C(2)	119.70(11)
O(2)-C(2)-C(3)	119.11(11)
O(2)-C(2)-C(1)	118.71(11)
C(3)-C(2)-C(1)	122.17(12)
C(2)-C(3)-C(4)	118.45(11)
C(2)-C(3)-C(18)	117.67(11)
C(4)-C(3)-C(18)	123.88(11)
C(7)-O(4)-C(8)	123.67(11)
C(5)-C(4)-C(12)	114.16(10)
C(5)-C(4)-C(3)	120.06(11)
C(12)-C(4)-C(3)	125.77(11)
C(6)-C(5)-C(9)	118.47(11)
C(6)-C(5)-C(4)	119.27(11)
C(9)-C(5)-C(4)	122.26(11)
C(1)-C(6)-C(5)	120.35(12)
C(1)-C(6)-C(7)	118.78(11)
C(5)-C(6)-C(7)	120.86(12)
C(12)-O(7)-C(13)	106.46(10)
O(3)-C(7)-O(4)	116.09(13)
O(3)-C(7)-C(6)	125.61(13)
O(4)-C(7)-C(6)	118.30(12)
O(5)-C(8)-O(4)	115.40(12)
O(5)-C(8)-C(9)	126.37(13)
O(4)-C(8)-C(9)	118.24(12)
C(10)-C(9)-C(5)	120.32(11)

C(10)-C(9)-C(8)	119.22(12)
C(5)-C(9)-C(8)	120.46(12)
O(6)-C(10)-C(11)	117.87(12)
O(6)-C(10)-C(9)	123.46(12)
C(11)-C(10)-C(9)	118.68(11)
C(12)-C(11)-C(10)	120.36(12)
C(12)-C(11)-C(14)	109.33(11)
C(10)-C(11)-C(14)	130.01(12)
O(7)-C(12)-C(11)	112.27(11)
O(7)-C(12)-C(4)	123.54(11)
C(11)-C(12)-C(4)	124.18(12)
O(7)-C(13)-C(15)	108.93(12)
O(7)-C(13)-C(14)	105.37(11)
C(15)-C(13)-C(14)	118.43(13)
C(11)-C(14)-C(16)	115.53(14)
C(11)-C(14)-C(17)	109.26(15)
C(16)-C(14)-C(17)	110.33(15)
C(11)-C(14)-C(13)	98.84(10)
C(16)-C(14)-C(13)	109.51(15)
C(17)-C(14)-C(13)	113.00(13)

S53. Bond lengths [Å] and angles [°] for Compound **8**

	U^{11}	U^{22}	U^{33}	U^{23}	U^{13}	U^{12}
O(1)	55(1)	26(1)	22(1)	8(1)	-1(1)	1(1)
C(1)	31(1)	20(1)	22(1)	5(1)	0(1)	0(1)
O(2)	62(1)	15(1)	30(1)	5(1)	-1(1)	1(1)
C(2)	32(1)	15(1)	25(1)	2(1)	0(1)	0(1)
O(3)	70(1)	33(1)	20(1)	3(1)	-2(1)	-1(1)
C(3)	27(1)	17(1)	22(1)	1(1)	0(1)	0(1)
O(4)	50(1)	23(1)	21(1)	-2(1)	0(1)	-1(1)
C(4)	25(1)	16(1)	20(1)	2(1)	0(1)	-1(1)
O(5)	52(1)	20(1)	34(1)	-4(1)	1(1)	0(1)
C(5)	26(1)	17(1)	20(1)	1(1)	0(1)	0(1)
O(6)	67(1)	15(1)	30(1)	2(1)	2(1)	1(1)
C(6)	28(1)	19(1)	20(1)	2(1)	1(1)	-1(1)
O(7)	40(1)	20(1)	19(1)	2(1)	-2(1)	1(1)
C(7)	36(1)	23(1)	22(1)	0(1)	1(1)	-1(1)
C(8)	31(1)	20(1)	24(1)	-2(1)	1(1)	0(1)
C(9)	30(1)	16(1)	22(1)	1(1)	1(1)	1(1)
C(10)	35(1)	16(1)	25(1)	2(1)	1(1)	2(1)
C(11)	34(1)	17(1)	23(1)	4(1)	1(1)	1(1)
C(12)	27(1)	19(1)	19(1)	2(1)	1(1)	-1(1)
C(13)	34(1)	25(1)	22(1)	6(1)	-1(1)	2(1)
C(14)	43(1)	20(1)	22(1)	5(1)	2(1)	2(1)
C(15)	50(1)	34(1)	22(1)	3(1)	2(1)	1(1)
C(16)	82(1)	35(1)	32(1)	7(1)	-4(1)	25(1)
C(17)	59(1)	35(1)	39(1)	4(1)	9(1)	-17(1)
C(18)	46(1)	19(1)	25(1)	-2(1)	-2(1)	-1(1)

S54. Anisotropic displacement parameters (Å2x 103) for Compound **8** . The anisotropic displacement factor exponent takes the form: -2p2[h2 a*2U11 + ... + 2 h k a* b* U12]

	x	y	z	U(eq)
H(1)	7664	1081	8565	52
H(2)	7618	-1078	7639	54
H(6)	7412	7028	6923	56
H(13)	5016	3790	5159	32
H(15A)	8699	3371	4571	53
H(15B)	6926	4200	4297	53
H(15C)	6644	2678	4419	53
H(16C)	4369	5962	5458	74
H(16A)	5661	6332	4899	74
H(16B)	6109	7012	5511	74
H(17A)	9742	6257	5532	66
H(17B)	9369	5736	4886	66
H(17C)	10240	4778	5370	66
H(18A)	5985	-17	6067	45
H(18B)	8066	-630	6254	45
H(18C)	7999	613	5834	45

S55. Hydrogen coordinates (x 104) and isotropic displacement parameters (Å2x 10 3) for Compound **8**

O(1)-C(1)-C(2)-O(2)	0.1(2)	O(5)-C(8)-C(9)-C(10)	-0.6(3)
C(6)-C(1)-C(2)-O(2)	179.72(14)	O(4)-C(8)-C(9)-C(10)	-179.88(15)
O(1)-C(1)-C(2)-C(3)	-179.82(15)	O(5)-C(8)-C(9)-C(5)	178.83(17)
C(6)-C(1)-C(2)-C(3)	-0.2(2)	O(4)-C(8)-C(9)-C(5)	-0.5(2)
O(2)-C(2)-C(3)-C(4)	179.86(14)	C(5)-C(9)-C(10)-O(6)	-178.07(16)
C(1)-C(2)-C(3)-C(4)	-0.2(2)	C(8)-C(9)-C(10)-O(6)	1.3(3)
O(2)-C(2)-C(3)-C(18)	0.3(2)	C(5)-C(9)-C(10)-C(11)	1.9(2)
C(1)-C(2)-C(3)-C(18)	-179.81(14)	C(8)-C(9)-C(10)-C(11)	-178.75(15)
C(2)-C(3)-C(4)-C(5)	0.6(2)	O(6)-C(10)-C(11)-C(12)	177.72(16)
C(18)-C(3)-C(4)-C(5)	-179.88(14)	C(9)-C(10)-C(11)-C(12)	-2.2(2)
C(2)-C(3)-C(4)-C(12)	-178.73(14)	O(6)-C(10)-C(11)-C(14)	4.7(3)
C(18)-C(3)-C(4)-C(12)	0.8(2)	C(9)-C(10)-C(11)-C(14)	-175.26(17)
C(12)-C(4)-C(5)-C(6)	178.89(13)	C(13)-O(7)-C(12)-C(11)	-14.26(18)
C(3)-C(4)-C(5)-C(6)	-0.5(2)	C(13)-O(7)-C(12)-C(4)	166.55(14)
C(12)-C(4)-C(5)-C(9)	-1.1(2)	C(10)-C(11)-C(12)-O(7)	-178.30(13)
C(3)-C(4)-C(5)-C(9)	179.52(14)	C(14)-C(11)-C(12)-O(7)	-3.9(2)
O(1)-C(1)-C(6)-C(5)	179.87(14)	C(10)-C(11)-C(12)-C(4)	0.9(3)
C(2)-C(1)-C(6)-C(5)	0.3(2)	C(14)-C(11)-C(12)-C(4)	175.26(14)
O(1)-C(1)-C(6)-C(7)	0.2(2)	C(5)-C(4)-C(12)-O(7)	179.87(13)
C(2)-C(1)-C(6)-C(7)	-179.41(14)	C(3)-C(4)-C(12)-O(7)	-0.8(2)
C(9)-C(5)-C(6)-C(1)	-179.94(14)	C(5)-C(4)-C(12)-C(11)	0.8(2)
C(4)-C(5)-C(6)-C(1)	0.1(2)	C(3)-C(4)-C(12)-C(11)	-179.91(15)
C(9)-C(5)-C(6)-C(7)	-0.3(2)	C(12)-O(7)-C(13)-C(15)	154.23(13)
C(4)-C(5)-C(6)-C(7)	179.74(15)	C(12)-O(7)-C(13)-C(14)	26.18(15)
C(8)-O(4)-C(7)-O(3)	179.57(15)	C(12)-C(11)-C(14)-C(16)	135.64(17)
C(8)-O(4)-C(7)-C(6)	-0.1(3)	C(10)-C(11)-C(14)-C(16)	-50.7(3)
C(1)-C(6)-C(7)-O(3)	0.1(3)	C(12)-C(11)-C(14)-C(17)	-99.27(16)
C(5)-C(6)-C(7)-O(3)	-179.57(17)	C(10)-C(11)-C(14)-C(17)	74.4(2)
C(1)-C(6)-C(7)-O(4)	179.79(14)	C(12)-C(11)-C(14)-C(13)	18.97(18)
C(5)-C(6)-C(7)-O(4)	0.1(2)	C(10)-C(11)-C(14)-C(13)	-167.38(17)
C(7)-O(4)-C(8)-O(5)	-179.06(16)	O(7)-C(13)-C(14)-C(11)	-26.44(15)
C(7)-O(4)-C(8)-C(9)	0.3(2)	C(15)-C(13)-C(14)-C(11)	-148.54(14)
C(6)-C(5)-C(9)-C(10)	179.84(13)	O(7)-C(13)-C(14)-C(16)	-147.63(13)
C(4)-C(5)-C(9)-C(10)	-0.2(2)	C(15)-C(13)-C(14)-C(16)	90.27(18)
C(6)-C(5)-C(9)-C(8)	0.5(2)	O(7)-C(13)-C(14)-C(17)	88.94(15)
C(4)-C(5)-C(9)-C(8)	-179.54(13)	C(15)-C(13)-C(14)-C(17)	-33.16(19)

S56. Torsion angles [°] for Compound **8**

D-H...A	d(D-H)	d(H...A)	d(D...A)	<(DHA)
O(1)-H(1)...O(3)	0.82	1.91	2.6218(16)	144.1
O(2)-H(2)...O(1)	0.82	2.20	2.6629(15)	115.8
O(2)-H(2)...O(5)#1	0.82	2.31	2.7358(15)	112.6
O(6)-H(6)...O(2)#2	0.82	2.28	2.9224(15)	135.0
O(6)-H(6)...O(5)	0.82	1.94	2.6528(16)	144.6
C(15)-H(15A)...O(7)#3	0.96	2.55	3.462(2)	158.9
C(15)-H(15B)...O(4)#4	0.96	2.61	3.4514(17)	146.7

Symmetry transformations used to generate equivalent atoms:

#1 x,y-1,z #2 x,y+1,z #3 x+1/2,-y+1/2,-z+1

#4 -x+3/2,-y+1,z-1/2

S57. Hydrogen bonds for Compound **8** [Å and °].

S58. Tiny crystalline needles of compound (**7**) could be also characterized with that intense copper radiation home source and atomic connectivity was provided with confidence despite medium-resolution data. The absolute configuration C13 R could be only derived by optical measurements.
Compound (**10**) and its absolute configuration C13 R was described elsewhere isolated from *Aspergillus silvaticus* (CSD Refcode ATRVNT). We could confirm its 3D structure based on XRD data collected on our Mo µ-source diffractometer, the chosen enantiomer relying only upon the former work and current optical measurement.

Crystal data for **7**: $C_{18}H_{16}O_6$, H_2O, M = 346.32, Monoclinic, a = 6.6249(2) Å, b = 10.0615(3) Å, c = 22.5473(6) Å, .a = 90°,b = 104.19(2)°, g = 90°, V = 797.7(8) Å^3, T = 213(2) K, space group $P2_1$, Z = 2, μ(Cu Kα) = 0.942 mm^{-1}, 1001 reflections measured, 454 independent reflections (Rint = 0.0928). The final R1 value was 0.0901 (I > 2σ(I)). The final *wR* (*F*2) value was 0.2766 (all data). The goodness of fit on F^2 was 1.365.

Crystal data for **10**: $C_{18}H_{16}O_{16}$, M = 328.31, Orthorhombic, a = 6.8026(11) Å, b = 9.890(2) Å, c = 22.518(4) Å, a = b = g = 90°, V = 1515.0 (5) Å^3, T = 293(2) K, space group $P2_12_12_1$, Z = 4, μ(Mo Kα) = 0.109 mm^{-1}, 8,982 reflections measured, 3079 independent reflections (Rint = 0.0688). The final R1 value was 0.0604 (I > 2σ(I)). The final *wR* (*F*2) value was 0.1821 (all data).

Crystallographic data for the structures (**7** and **10** have been deposited in the Cambridge. Crystallographic Data Centre database (deposition numbers CCDC 1964262-1963851-1964548 respectively). Copies of the data can be obtained free of charge from the CCDC at www.ccdc.cam.ac.uk.

Identification code	**(+)-Sclerodereolide**	
Empirical formula	$C_{18} H_{16} O_6$, $H_2 O$	
Formula weight	346.32	
Temperature	213(2) K	
Wavelength	1.54187 Å	
Crystal system	Monoclinic	
Space group	$P2_1$	
Unit cell dimensions	a = 10.171(6) Å	α= 90°.
	b = 6.905(4) Å	β= 104.19(2)°.
	c = 11.716(8) Å	γ = 90°.
Volume	797.7(8) $Å^3$	
Z	2	
Density (calculated)	1.442 Mg/m^3	
Absorption coefficient	0.942 mm^{-1}	
F(000)	364	
Crystal size	0.24 x 0.02 x 0.01 mm^3	
Theta range for data collection	3.892 to 30.893°.	
Index ranges	$-6 \leq h \leq 6$, $-4 \leq k \leq 3$, $-7 \leq l \leq 7$	
Reflections collected	1001	
Independent reflections	454 [R(int) = 0.0928]	
Completeness to theta = 30.893°	97.2 %	
Absorption correction	Semi-empirical from equivalents	
Max. and min. transmission	1.000 and 0.064	
Refinement method	Full-matrix least-squares on F^2	
Data / restraints / parameters	454 / 325 / 215	
Goodness-of-fit on F^2	1.365	
Final R indices [I>2σ(I)]	R1 = 0.0901, wR2 = 0.2263	
R indices (all data)	R1 = 0.1078 wR2 = 0.2766	
Largest diff. peak and hole	0.264 and -0.236 e.$Å^{-3}$	

S59. Crystal data and structure refinement for Compound **7**.

	x	y	z	U(eq)
O(3)	10290(40)	3030(100)	11780(30)	95(16)
O(1)	12980(30)	2930(80)	12030(30)	96(15)
C(1)	12120(40)	2990(80)	10930(30)	63(19)
C(2)	12690(30)	3060(90)	9970(40)	75(18)
C(3)	11850(50)	3150(100)	8830(30)	74(19)
C(4)	10450(40)	3180(100)	8670(30)	80(20)
C(5)	9890(30)	3120(100)	9640(40)	70(20)
C(6)	10720(40)	3020(90)	10770(30)	49(17)
O(2)	8590(40)	3180(100)	12620(30)	106(16)
O(4)	6700(50)	2990(100)	10490(30)	125(19)
C(10)	7670(70)	2970(120)	8450(60)	69(18)
C(7)	8940(70)	3250(150)	11660(60)	70(20)
C(11)	8030(60)	3230(140)	7400(60)	77(17)
C(8)	7930(60)	3290(140)	10560(40)	80(20)
C(12)	9480(70)	3240(130)	7570(60)	70(20)
C(9)	8480(40)	3180(150)	9570(60)	79(18)
O(5)	6270(40)	3030(100)	8260(30)	119(18)
O(6)	9850(30)	3310(80)	6550(30)	87(16)
C(17)	7200(70)	800(130)	5730(60)	140(30)
C(15)	8550(80)	5920(120)	5390(60)	140(30)
C(14)	7410(80)	2940(120)	6090(60)	110(20)
C(13)	8560(80)	3750(140)	5640(70)	120(30)
C(16)	6080(60)	4050(110)	5680(60)	140(30)
C(18)	12540(40)	2950(150)	7750(40)	110(20)
O(1W)	15620(40)	3400(120)	12430(40)	180(30)

S60. Atomic coordinates (x 104) and equivalent isotropic displacement parameters (Å2x 103) for Compound **7**. U(eq) is defined as one third of the trace of the orthogonalized Uij tensor.

O(3)-C(7)	1.35(6)	C(4)-O(1)-C(2)	113(6)	C(10)-C(9)-C(5)	114(6)
O(3)-C(6)	1.36(4)	O(4)-C(1)-C(6)	129(8)	C(8)-C(9)-C(5)	124(6)
O(1)-C(1)	1.36(3)	O(4)-C(1)-C(2)	113(8)	C(12)-O(6)-C(13)	104(5)
C(1)-C(2)	1.3900	C(6)-C(1)-C(2)	114(8)	C(13)-C(14)-C(11)	98(7)
C(1)-C(6)	1.3900	O(3)-C(2)-O(1)	107(7)	C(13)-C(14)-C(16)	114(6)
C(2)-C(3)	1.3900	O(3)-C(2)-C(1)	127(8)	C(11)-C(14)-C(16)	111(7)
C(3)-C(4)	1.3900	O(1)-C(2)-C(1)	126(8)	C(13)-C(14)-C(17)	109(6)
C(3)-C(18)	1.60(5)	O(1)-C(4)-C(5)	126(6)	C(11)-C(14)-C(17)	114(7)
C(4)-C(5)	1.3900	O(1)-C(4)-C(13)	114(5)	C(16)-C(14)-C(17)	110(8)
C(4)-C(12)	1.42(6)	C(5)-C(4)-C(13)	119(7)	C(14)-C(13)-O(6)	107(6)
C(5)-C(6)	1.3900	C(6)-C(5)-C(10)	120.0	C(14)-C(13)-C(15)	118(8)
C(5)-C(9)	1.42(4)	C(6)-C(5)-C(4)	117(6)	O(6)-C(13)-C(15)	107(7)
O(2)-C(7)	1.26(4)	C(10)-C(5)-C(4)	123(6)		
O(4)-C(8)	1.24(5)	C(1)-C(6)-C(7)	116(6)		
C(10)-C(11)	1.38(4)	C(1)-C(6)-C(5)	123(7)		
C(10)-C(9)	1.38(4)	C(7)-C(6)-C(5)	120.0		
C(10)-O(5)	1.38(6)	O(5)-C(7)-C(8)	117(5)		
C(7)-C(8)	1.44(6)	O(5)-C(7)-C(6)	122(5)		
C(11)-C(12)	1.43(6)	C(8)-C(7)-C(6)	120.0		
C(11)-C(14)	1.52(8)	C(7)-C(8)-C(9)	120.0		
C(8)-C(9)	1.41(6)	C(7)-C(8)-C(16)	130(5)		
C(12)-O(6)	1.34(6)	C(9)-C(8)-C(16)	109(5)		
O(6)-C(13)	1.51(7)	O(6)-C(9)-C(10)	127(5)		
C(17)-C(14)	1.54(5)	O(6)-C(9)-C(8)	113(5)		
C(15)-C(13)	1.52(5)	C(10)-C(9)-C(8)	120.0		
C(14)-C(13)	1.50(10)	C(9)-C(10)-C(5)	120.0		
C(14)-C(16)	1.53(5)	C(9)-C(10)-C(11)	122(5)		

S61. Bond lengths [Å] and angles [°] for Compound **7**.

	U^{11}	U^{22}	U^{33}	U^{23}	U^{13}	U^{12}
O(3)	70(20)	140(40)	90(30)	0(40)	40(20)	30(40)
O(1)	60(30)	110(40)	110(20)	-20(30)	10(19)	0(30)
C(1)	60(20)	60(30)	80(20)	-10(30)	25(17)	0(30)
C(2)	50(30)	80(30)	100(20)	0(30)	32(19)	0(30)
C(3)	80(30)	60(30)	90(20)	-10(30)	28(19)	0(30)
C(4)	80(30)	100(50)	50(30)	-40(50)	18(19)	-10(50)
C(5)	60(20)	70(50)	70(30)	0(50)	25(18)	20(40)
C(6)	50(20)	30(30)	70(20)	-10(30)	25(16)	10(30)
O(2)	80(30)	180(40)	80(20)	0(40)	40(20)	10(40)
O(4)	70(30)	200(50)	110(30)	20(50)	30(20)	0(40)
C(10)	60(20)	70(40)	80(20)	10(30)	21(19)	10(30)
C(7)	60(20)	90(40)	80(20)	0(30)	30(20)	0(30)
C(11)	60(20)	90(40)	80(20)	10(30)	30(20)	0(30)
C(8)	70(30)	80(40)	80(20)	10(30)	20(20)	0(30)
C(12)	70(30)	100(60)	60(30)	-30(50)	30(20)	-20(40)
C(9)	70(20)	90(40)	80(20)	0(30)	16(17)	10(30)
O(5)	60(20)	200(50)	110(30)	-10(50)	30(20)	0(40)
O(6)	90(30)	130(40)	50(30)	-10(40)	30(20)	30(40)
C(17)	150(70)	180(60)	70(60)	20(40)	-30(50)	-20(50)
C(15)	120(50)	130(50)	150(60)	0(40)	10(40)	10(40)
C(14)	90(30)	170(50)	70(30)	20(40)	10(30)	10(40)
C(13)	100(40)	160(50)	90(40)	40(60)	20(30)	20(60)
C(16)	80(40)	200(70)	120(50)	40(40)	20(40)	0(40)
C(18)	70(40)	170(60)	100(30)	-20(50)	30(30)	-10(50)
O(1W)	100(40)	310(60)	130(40)	0(50)	50(30)	0(50)

S62. Anisotropic displacement parameters (Å2x 103) for Compound **7**. The anisotropic displacement factor exponent takes the form: -2p2[h2 a*2U11 + ... + 2 h k a* b* U12]

	x	y	z	U(eq)
H(1O)	12525	2919	12531	144
H(2)	13637	3039	10077	90
H(5O)	6061	3023	8897	179
H(17A)	6464	270	6020	213
H(17B)	6986	700	4877	213
H(17C)	8028	80	6062	213
H(15A)	9338	6252	5105	204
H(15B)	7732	6249	4796	204
H(15C)	8568	6633	6106	204
H(13)	8582	3056	4909	141
H(16A)	5388	3441	6002	204
H(16B)	6204	5377	5947	204
H(16C)	5793	4021	4825	204
H(18A)	13518	2960	8040	168
H(18B)	12255	1747	7340	168
H(18C)	12261	4031	7215	168
H(1W1)	14794	3104	12278	270
H(1W2)	15872	3283	11806	270

S63. Hydrogen coordinates (x 104) and isotropic displacement parameters (Å2x 10 3) for Compound **7**.

O(1)-C(1)-C(2)-C(3)	-179(5)	O(5)-C(10)-C(11)-C(12)	178(7)	C(4)-C(5)-C(9)-C(8)	-174(7)
C(6)-C(1)-C(2)-C(3)	0.0	C(9)-C(10)-C(11)-C(14)	173(10)	C(6)-C(5)-C(9)-C(8)	4(11)
C(1)-C(2)-C(3)-C(4)	0.0	O(5)-C(10)-C(11)-C(14)	-25(15)	C(11)-C(12)-O(6)-C(13)	11(10)
C(1)-C(2)-C(3)-C(18)	-172(5)	O(2)-C(7)-C(8)-O(4)	-10(18)	C(4)-C(12)-O(6)-C(13)	-169(7)
C(2)-C(3)-C(4)-C(5)	0.0	O(3)-C(7)-C(8)-O(4)	160(8)	C(10)-C(11)-C(14)-C(13)	177(11)
C(18)-C(3)-C(4)-C(5)	172(5)	O(2)-C(7)-C(8)-C(9)	-174(7)	C(12)-C(11)-C(14)-C(13)	-26(9)
C(2)-C(3)-C(4)-C(12)	-179(7)	O(3)-C(7)-C(8)-C(9)	-4(17)	C(10)-C(11)-C(14)-C(16)	57(15)
C(18)-C(3)-C(4)-C(12)	-7(8)	C(10)-C(11)-C(12)-O(6)	174(7)	C(12)-C(11)-C(14)-C(16)	-146(7)
C(3)-C(4)-C(5)-C(6)	0.0	C(14)-C(11)-C(12)-O(6)	10(11)	C(10)-C(11)-C(14)-C(17)	-68(13)
C(12)-C(4)-C(5)-C(6)	179(6)	C(10)-C(11)-C(12)-C(4)	-5(13)	C(12)-C(11)-C(14)-C(17)	90(10)
C(3)-C(4)-C(5)-C(9)	178(6)	C(14)-C(11)-C(12)-C(4)	-170(8)	C(11)-C(14)-C(13)-O(6)	32(8)
C(12)-C(4)-C(5)-C(9)	-2(8)	C(3)-C(4)-C(12)-O(6)	-1(11)	C(16)-C(14)-C(13)-O(6)	150(7)
C(7)-O(3)-C(6)-C(5)	-4(9)	C(5)-C(4)-C(12)-O(6)	-180(6)	C(17)-C(14)-C(13)-O(6)	-86(8)
C(7)-O(3)-C(6)-C(1)	174(7)	C(3)-C(4)-C(12)-C(11)	179(6)	C(11)-C(14)-C(13)-C(15)	-89(8)
C(4)-C(5)-C(6)-O(3)	177(5)	C(5)-C(4)-C(12)-C(11)	0(11)	C(16)-C(14)-C(13)-C(15)	29(10)
C(9)-C(5)-C(6)-O(3)	-1(7)	C(11)-C(10)-C(9)-C(8)	166(9)	C(17)-C(14)-C(13)-C(15)	153(7)
C(4)-C(5)-C(6)-C(1)	0.0	O(5)-C(10)-C(9)-C(8)	6(15)	C(12)-O(6)-C(13)-C(14)	-29(9)
C(9)-C(5)-C(6)-C(1)	-179(6)	C(11)-C(10)-C(9)-C(5)	-19(14)	C(12)-O(6)-C(13)-C(15)	99(7)
O(1)-C(1)-C(6)-O(3)	1(5)	O(5)-C(10)-C(9)-C(5)	-179(6)		
C(2)-C(1)-C(6)-O(3)	-178(4)	O(4)-C(8)-C(9)-C(10)	8(16)		
O(1)-C(1)-C(6)-C(5)	179(5)	C(7)-C(8)-C(9)-C(10)	172(9)		
C(2)-C(1)-C(6)-C(5)	0.0	O(4)-C(8)-C(9)-C(5)	-166(8)		
C(6)-O(3)-C(7)-O(2)	178(6)	C(7)-C(8)-C(9)-C(5)	-2(15)		
C(6)-O(3)-C(7)-C(8)	7(15)	C(4)-C(5)-C(9)-C(10)	11(11)		
C(9)-C(10)-C(11)-C(12)	16(14)	C(6)-C(5)-C(9)-C(10)	-171(6)		

S64. Torsion angles [°] for Compound **7**.

D-H...A	d(D-H)	d(H...A)	d(D...A)	<(DHA)
O(1)-H(1O)...O(3)	0.83	2.23	2.68(5)	114.1
O(5)-H(5O)...O(4)	0.83	1.82	2.55(5)	145.3
O(1W)-H(1W1)...O(1)	0.84	1.80	2.63(5)	169.0
O(1W)-H(1W2)...O(4)#1	0.84	1.94	2.77(6)	172.3

Symmetry transformations used to generate equivalent atoms:
#1 x+1,y,z

S65. Hydrogen bonds for Compound **7** [Å and °].

Identification code	**(+)-Sclerodin**	
Empirical formula	$C_{18} H_{16} O_6$	
Formula weight	328.31	
Temperature	293.0 K	
Wavelength	0.71073 Å	
Crystal system	Orthorhombic	
Space group	$P2_12_12_1$	
Unit cell dimensions	a = 6.8026(11) Å	α= 90°.
	b = 9.890(2) Å	β= 90°.
	c = 22.518(4) Å	γ = 90°.
Volume	1515.0(5) $Å^3$	
Z	4	
Density (calculated)	1.439 Mg/m^3	
Absorption coefficient	0.109 mm^{-1}	
F(000)	688	
Crystal size	0.150 x 0.025 x 0.01 mm^3	
Theta range for data collection	4.121 to 26.369°.	
Index ranges	$-8 \leq h \leq 8, -12 \leq k \leq 12, -27 \leq l \leq 28$	
Reflections collected	8982	
Independent reflections	3079 [R(int) = 0.0688]	
Completeness to theta = 25.242°	99.1 %	
Absorption correction	Multi-scan	
Max. and min. transmission	1.000 and 0.796	
Refinement method	Full-matrix least-squares on F^2	
Data / restraints / parameters	3074 / 0 / 224	
Goodness-of-fit on F^2	0.988	
Final R indices [I>2σ(*I*)]	R1 = 0.06084, wR2 = 0.1449	
R indices (all data)	R1 = 0.1276, wR2 = 0.1821	
Largest diff. peak and hole	0.192 and -0.174 e.$Å^{-3}$	

S66. Crystal data and structure refinement for Compound **10**.

	x	y	z	U(eq)
O(1)	2938(9)	-308(4)	4480(2)	91(1)
O(2)	2907(7)	1637(5)	3689(2)	93(1)
O(3)	2917(7)	3769(5)	3974(1)	76(1)
O(4)	2950(9)	5946(4)	4196(2)	94(1)
O(5)	2994(9)	6761(3)	5307(2)	84(1)
O(6)	2952(6)	3524(3)	6776(1)	58(1)
C(1)	2928(9)	690(5)	4884(2)	63(1)
C(2)	2914(10)	311(5)	5476(2)	64(1)
C(3)	2908(9)	1255(5)	5920(2)	53(1)
C(4)	2930(8)	2656(4)	5774(2)	42(1)
C(5)	2914(8)	3046(4)	5169(2)	44(1)
C(6)	2928(8)	2041(5)	4723(2)	52(1)
C(7)	2920(10)	2412(7)	4107(2)	66(1)
C(8)	2935(10)	4805(6)	4392(2)	68(1)
C(9)	2953(9)	4421(4)	5010(2)	51(1)
C(10)	2985(9)	5439(4)	5441(2)	56(1)
C(11)	3003(9)	5072(4)	6038(2)	50(1)
C(12)	2963(8)	3737(4)	6184(2)	47(1)
C(13)	3239(10)	4824(5)	7074(2)	66(2)
C(14)	2981(10)	5941(5)	6595(2)	58(1)
C(15)	1937(16)	4857(7)	7603(3)	113(3)
C(16)	4735(10)	6897(7)	6633(3)	90(2)
C(17)	1073(10)	6727(7)	6642(3)	90(2)
C(18)	2876(12)	768(5)	6555(3)	78(2)

S67. Atomic coordinates (x 104) and equivalent isotropic displacement parameters (Å2x 103) for Compound **10**. U(eq) is defined as one third of the trace of the orthogonalized Uij tensor.

O(1)-C(1)	1.342(6)	C(1)-O(1)-H(1)	109.5	O(6)-C(13)-H(13)	107.8
O(1)-H(1)	0.8200	C(7)-O(3)-C(8)	124.9(4)	C(15)-C(13)-H(13)	107.8
O(2)-C(7)	1.214(6)	C(10)-O(5)-H(5)	109.5	C(14)-C(13)-H(13)	107.8
O(3)-C(7)	1.375(7)	C(12)-O(6)-C(13)	108.3(3)	C(17)-C(14)-C(11)	110.9(5)
O(3)-C(8)	1.390(6)	O(1)-C(1)-C(6)	122.2(5)	C(17)-C(14)-C(16)	110.4(4)
O(4)-C(8)	1.211(6)	O(1)-C(1)-C(2)	117.0(5)	C(11)-C(14)-C(16)	112.9(5)
O(5)-C(10)	1.342(5)	C(6)-C(1)-C(2)	120.8(4)	C(17)-C(14)-C(13)	114.3(5)
O(5)-H(5)	0.8200	C(3)-C(2)-C(1)	121.2(4)	C(11)-C(14)-C(13)	99.7(3)
O(6)-C(12)	1.350(5)	C(3)-C(2)-H(2)	119.4	C(16)-C(14)-C(13)	108.2(5)
O(6)-C(13)	1.464(5)	C(1)-C(2)-H(2)	119.4	C(13)-C(15)-H(15A)	109.5
C(1)-C(6)	1.385(7)	C(2)-C(3)-C(4)	119.7(4)	C(13)-C(15)-H(15B)	109.5
C(1)-C(2)	1.385(8)	C(2)-C(3)-C(18)	118.3(4)	H(15A)-C(15)-H(15B)	109.5
C(2)-C(3)	1.368(7)	C(4)-C(3)-C(18)	122.0(4)	C(13)-C(15)-H(15C)	109.5
C(2)-H(2)	0.9300	C(12)-C(4)-C(5)	115.0(3)	H(15A)-C(15)-H(15C)	109.5
C(3)-C(4)	1.424(6)	C(12)-C(4)-C(3)	125.8(4)	H(15B)-C(15)-H(15C)	109.5
C(3)-C(18)	1.509(7)	C(5)-C(4)-C(3)	119.1(4)	C(14)-C(16)-H(16A)	109.5
C(4)-C(12)	1.413(6)	C(9)-C(5)-C(6)	120.0(4)	C(14)-C(16)-H(16B)	109.5
C(4)-C(5)	1.416(6)	C(9)-C(5)-C(4)	120.5(4)	H(16A)-C(16)-H(16B)	109.5
C(5)-C(9)	1.407(6)	C(6)-C(5)-C(4)	119.5(4)	C(14)-C(16)-H(16C)	109.5
C(5)-C(6)	1.412(6)	C(1)-C(6)-C(5)	119.6(4)	H(16A)-C(16)-H(16C)	109.5
C(6)-C(7)	1.436(7)	C(1)-C(6)-C(7)	119.9(5)	H(16B)-C(16)-H(16C)	109.5
C(8)-C(9)	1.444(7)	C(5)-C(6)-C(7)	120.5(5)	C(14)-C(17)-H(17A)	109.5
C(9)-C(10)	1.398(7)	O(2)-C(7)-O(3)	116.6(5)	C(14)-C(17)-H(17B)	109.5
C(10)-C(11)	1.393(6)	O(2)-C(7)-C(6)	126.0(6)	H(17A)-C(17)-H(17B)	109.5
C(11)-C(12)	1.361(6)	O(3)-C(7)-C(6)	117.4(5)	C(14)-C(17)-H(17C)	109.5
C(11)-C(14)	1.520(6)	O(4)-C(8)-O(3)	116.2(5)	H(17A)-C(17)-H(17C)	109.5
C(13)-C(15)	1.485(9)	O(4)-C(8)-C(9)	126.5(5)	H(17B)-C(17)-H(17C)	109.5
C(13)-C(14)	1.555(7)	O(3)-C(8)-C(9)	117.3(5)	C(3)-C(18)-H(18A)	109.5
C(13)-H(13)	0.9800	C(10)-C(9)-C(5)	121.3(4)	C(3)-C(18)-H(18B)	109.5
C(14)-C(17)	1.516(8)	C(10)-C(9)-C(8)	118.7(4)	H(18A)-C(18)-H(18B)	109.5
C(14)-C(16)	1.524(8)	C(5)-C(9)-C(8)	120.0(5)	C(3)-C(18)-H(18C)	109.5
C(15)-H(15A)	0.9600	O(5)-C(10)-C(11)	118.1(4)	H(18A)-C(18)-H(18C)	109.5
C(15)-H(15B)	0.9600	O(5)-C(10)-C(9)	123.1(4)	H(18B)-C(18)-H(18C)	109.5
C(15)-H(15C)	0.9600	C(11)-C(10)-C(9)	118.8(4)		
C(16)-H(16A)	0.9600	C(12)-C(11)-C(10)	119.1(4)		
C(16)-H(16B)	0.9600	C(12)-C(11)-C(14)	110.4(4)		
C(16)-H(16C)	0.9600	C(10)-C(11)-C(14)	130.4(4)		
C(17)-H(17A)	0.9600	O(6)-C(12)-C(11)	113.0(4)		
C(17)-H(17B)	0.9600	O(6)-C(12)-C(4)	121.8(4)		
C(17)-H(17C)	0.9600	C(11)-C(12)-C(4)	125.2(4)		
C(18)-H(18A)	0.9600	O(6)-C(13)-C(15)	107.9(5)		
C(18)-H(18B)	0.9600	O(6)-C(13)-C(14)	106.9(4)		
C(18)-H(18C)	0.9600	C(15)-C(13)-C(14)	118.3(5)		

S68. Bond lengths [Å] and angles [°] for Compound **10**.

	U^{11}	U^{22}	U^{33}	U^{23}	U^{13}	U^{12}
O(1)	80(3)	103(3)	90(3)	-42(2)	1(3)	0(3)
O(2)	72(2)	151(4)	56(2)	-35(2)	-3(3)	0(4)
O(3)	59(2)	127(3)	41(2)	4(2)	-3(2)	-1(3)
O(4)	106(3)	113(3)	65(2)	40(2)	-1(3)	3(3)
O(5)	114(3)	65(2)	73(2)	20(2)	-6(4)	0(3)
O(6)	70(2)	62(2)	42(2)	1(1)	2(2)	3(2)
C(1)	39(3)	76(3)	75(4)	-30(3)	-3(4)	3(3)
C(2)	57(3)	57(3)	79(4)	-7(3)	0(4)	2(3)
C(3)	42(3)	62(3)	55(3)	-2(2)	2(3)	1(3)
C(4)	30(2)	50(2)	45(2)	2(2)	0(3)	1(3)
C(5)	24(2)	64(3)	45(2)	-3(2)	-2(3)	2(3)
C(6)	25(2)	86(3)	45(3)	-12(2)	-2(3)	1(3)
C(7)	36(3)	104(4)	59(3)	-7(3)	-2(3)	0(4)
C(8)	52(3)	103(4)	49(3)	16(3)	-4(3)	3(4)
C(9)	39(2)	68(3)	47(3)	7(2)	-1(3)	-3(3)
C(10)	54(3)	53(3)	60(3)	13(2)	-2(3)	3(3)
C(11)	46(3)	54(2)	48(3)	2(2)	-2(3)	-2(3)
C(12)	36(2)	59(2)	45(2)	2(2)	1(3)	5(3)
C(13)	74(4)	66(3)	57(3)	-9(3)	-9(3)	6(3)
C(14)	59(3)	57(3)	58(3)	-7(2)	1(3)	11(3)
C(15)	183(8)	101(4)	54(3)	-7(3)	42(5)	28(6)
C(16)	95(5)	87(4)	89(4)	-11(4)	1(4)	-29(4)
C(17)	94(5)	98(5)	79(4)	-12(4)	0(4)	30(4)
C(18)	100(5)	59(3)	74(4)	11(3)	3(4)	2(4)

S69. Anisotropic displacement parameters (Å2x 103) for Compound **10**. The anisotropic displacement factor exponent takes the form: -2p2[h2 a*2U11 + ... + 2 h k a* b* U12]

	x	y	z	U(eq)
H(1)	2987	18	4146	136
H(5)	2945	6855	4946	126
H(2)	2909	-603	5574	77
H(13)	4602	4859	7214	79
H(15A)	2226	4099	7855	169
H(15B)	589	4814	7478	169
H(15C)	2154	5681	7819	169
H(16A)	4652	7555	6321	136
H(16B)	5930	6389	6592	136
H(16C)	4726	7348	7010	136
H(17A)	1129	7317	6980	135
H(17B)	-3	6107	6687	135
H(17C)	886	7253	6288	135
H(18A)	4083	1017	6747	116
H(18B)	2735	-197	6562	116
H(18C)	1790	1176	6760	116

S70. Hydrogen coordinates (x 104) and isotropic displacement parameters (Å2x 10 3) for Compound **10**.

O(1)-C(1)-C(2)-C(3)	179.8(6)	O(4)-C(8)-C(9)-C(5)	179.4(7)
C(6)-C(1)-C(2)-C(3)	-0.3(11)	O(3)-C(8)-C(9)-C(5)	-1.2(10)
C(1)-C(2)-C(3)-C(4)	-0.4(11)	C(5)-C(9)-C(10)-O(5)	-179.0(6)
C(1)-C(2)-C(3)-C(18)	179.5(6)	C(8)-C(9)-C(10)-O(5)	0.2(11)
C(2)-C(3)-C(4)-C(12)	-178.8(5)	C(5)-C(9)-C(10)-C(11)	0.8(10)
C(18)-C(3)-C(4)-C(12)	1.3(11)	C(8)-C(9)-C(10)-C(11)	-180.0(6)
C(2)-C(3)-C(4)-C(5)	1.4(10)	O(5)-C(10)-C(11)-C(12)	179.0(6)
C(18)-C(3)-C(4)-C(5)	-178.6(6)	C(9)-C(10)-C(11)-C(12)	-0.9(9)
C(12)-C(4)-C(5)-C(9)	0.7(8)	O(5)-C(10)-C(11)-C(14)	1.7(11)
C(3)-C(4)-C(5)-C(9)	-179.4(6)	C(9)-C(10)-C(11)-C(14)	-178.1(6)
C(12)-C(4)-C(5)-C(6)	178.5(5)	C(13)-O(6)-C(12)-C(11)	-7.1(7)
C(3)-C(4)-C(5)-C(6)	-1.6(9)	C(13)-O(6)-C(12)-C(4)	173.1(5)
O(1)-C(1)-C(6)-C(5)	179.9(5)	C(10)-C(11)-C(12)-O(6)	-178.9(5)
C(2)-C(1)-C(6)-C(5)	0.0(10)	C(14)-C(11)-C(12)-O(6)	-1.2(8)
O(1)-C(1)-C(6)-C(7)	0.6(10)	C(10)-C(11)-C(12)-C(4)	0.9(10)
C(2)-C(1)-C(6)-C(7)	-179.3(6)	C(14)-C(11)-C(12)-C(4)	178.7(5)
C(9)-C(5)-C(6)-C(1)	178.7(6)	C(5)-C(4)-C(12)-O(6)	179.0(5)
C(4)-C(5)-C(6)-C(1)	0.9(9)	C(3)-C(4)-C(12)-O(6)	-0.8(10)
C(9)-C(5)-C(6)-C(7)	-2.0(8)	C(5)-C(4)-C(12)-C(11)	-0.8(9)
C(4)-C(5)-C(6)-C(7)	-179.7(5)	C(3)-C(4)-C(12)-C(11)	179.3(6)
C(8)-O(3)-C(7)-O(2)	-179.8(5)	C(12)-O(6)-C(13)-C(15)	140.3(6)
C(8)-O(3)-C(7)-C(6)	0.5(10)	C(12)-O(6)-C(13)-C(14)	12.1(6)
C(1)-C(6)-C(7)-O(2)	0.2(11)	C(12)-C(11)-C(14)-C(17)	-112.7(5)
C(5)-C(6)-C(7)-O(2)	-179.1(6)	C(10)-C(11)-C(14)-C(17)	64.7(9)
C(1)-C(6)-C(7)-O(3)	179.9(6)	C(12)-C(11)-C(14)-C(16)	122.8(6)
C(5)-C(6)-C(7)-O(3)	0.6(9)	C(10)-C(11)-C(14)-C(16)	-59.8(9)
C(7)-O(3)-C(8)-O(4)	179.2(7)	C(12)-C(11)-C(14)-C(13)	8.1(7)
C(7)-O(3)-C(8)-C(9)	-0.2(10)	C(10)-C(11)-C(14)-C(13)	-174.5(6)
C(6)-C(5)-C(9)-C(10)	-178.5(5)	O(6)-C(13)-C(14)-C(17)	106.5(5)
C(4)-C(5)-C(9)-C(10)	-0.8(9)	C(15)-C(13)-C(14)-C(17)	-15.4(8)
C(6)-C(5)-C(9)-C(8)	2.3(9)	O(6)-C(13)-C(14)-C(11)	-11.8(6)
C(4)-C(5)-C(9)-C(8)	-180.0(6)	C(15)-C(13)-C(14)-C(11)	-133.7(6)
O(4)-C(8)-C(9)-C(10)	0.2(12)	O(6)-C(13)-C(14)-C(16)	-130.0(5)
O(3)-C(8)-C(9)-C(10)	179.6(5)	C(15)-C(13)-C(14)-C(16)	108.1(7)

S71. Torsion angles [°] for Compound**10**

D-H...A	d(D-H)	d(H...A)	d(D...A)	<(DHA)
O(1)-H(1)...O(2)	0.82	1.90	2.622(6)	145.7
O(5)-H(5)...O(4)	0.82	1.91	2.627(5)	145.4
C(15)-H(15C)...O(3)#1	0.96	2.66	3.374(7)	131.8

Symmetry transformations used to generate equivalent atoms:

#1 -x+1/2,-y+1,z+1/2

S72. Hydrogen bonds for Compound **10** [Å and °].
